# Oral and Posters, June 7

**DOI:** 10.3402/ejpt.v4i0.21098

**Published:** 2013-06-04

## XIII ESTSS CONFERENCE: “Trauma and its clinical pathways: PTSD and beyond”, Bologna, June 2013 ORAL, JUNE 7 A. PLENARY HALL

## Morning
*Keynote Address*

### Hormonal changes and the treatment of PTSD 8:45–9:45

#### M. Olff Academic Medical Center, Department of Psychiatry, University of Amsterdam, Amsterdam, The Netherlands

Post-traumatic stress disorder (PTSD) has been associated with several biological changes, most notably with dysregulation of the neuroendocrine system. Most is known about the HPA axis in trauma and PTSD, but neuropeptides like oxytocin-associated stress and social behavior-gain more interest. This presentation will focus on the role of stress-related hormones and neuropeptides in the acute response to trauma, in the development of PTSD following trauma and it will address exciting developments on the role of biology in the treatment of PTSD.

## Effects of trauma on families and children
*Symposium: Transgenerational transmission of trauma in diverse cultural and political settings*

### Transgenerational effects of traumatic experiences in families of former war children of World War II 10:00–10:15

#### M. Boettche^1^, E. Bernsen^2^ and C. Knaevelsrud^1^

^1^Center for Torture Victims, Freie University Berlin, Berlin, Germany; ^2^Center for Torture Victims, Julius-Maximilians-University, Wrzburg, Germany

*Background:* Empirical findings on transgenerational effects of parental traumatic events on the second generation remain ambiguous. This study examined transgenerational effects of early life-time traumatization in a sample of aging former children of the World War II and their children. *Method:* In a cross-sectional study, 51 parent-child pairs were assessed using self-rated questionnaires. Assessment included mental health (posttraumatic stress disorder [PTSD, PDS]; anxiety, depression, somatization [BSI]) as well as parenting style [FEE], and communication about their war experiences within the family. Based on the presence of parental PTSD, the sample was divided into two groups (PTSD group, *N*=34 parent-child pairs; non-PTSD group, *N*=17 parent-child pairs). *Results:* There were no significant differences between the groups concerning the children's psychopathology. Children's self-rated somatization and depression were significantly related to parent's somatization and depression (*r*=0.31, *p*<0.05, and *r*=0.35, *p*<0.05, respectively) in the PTSD group; however, not in the non-PTSD group. Concerning the perceived parenting style, children of the PTSD group experienced their parents as more negative and punitive (*U*=193.50, *z*=−1.96, *p*<.05, *r*=−0.27) as well as more controlling and overprotecting (*U*=120.00, *z*=−3.40, *p*<0.001, *r*=−0.48) compared to the non-PTSD group. Both groups of children did not differ in their perception of the emotional warmth of their parents. Furthermore, children of the PTSD group perceived their parents as more emotional during the communication about war experiences compared to the non-PTSD group (*U*=192.50, *z*=−2.01, *p*=0.02, *r*=−0.28). *Conclusion:* This study also did not identify a direct transmission of psychopathological stress. However, the results underpin the assumptions of indirect transgenerational processes concerning the parental education and communication.

### Violence following the Rwandan genocide: the role of childhood maltreatment in the transmission of trauma from parent to child 10:15–10:30

#### H. Rieder and T. Elbert University of Konstanz, Konstanz, Germany

Researchers still debate whether mental disorders from the trauma spectrum are transferred from one generation to another and-if so-discuss which mechanisms might be relevant in understanding distress in the second generation (Kellerman, 2001; Yehuda, 2001). The objective of this study is to examine these phenomena in post-genocide Rwanda, while further focusing on family violence as a potential factor. In a cross-sectional survey, 173 parent-child pairs were randomly selected from four sectors in Muhanga, Southern Province of Rwanda and interviewed by local psychologists. All respondents completed an event scale (Schaal & Elbert, 2006), the PTSD Symptom Scale-Interview (PSS-I, Foa & Tolin 2000) and the childhood trauma questionnaire (CTQ, Bernstein et al. 1994). In addition, descendants completed the Hopkins Symptom Checklist (HSCL-25, Derogatis 1974). Ordinal regression analyses showed that the parents' exposure to childhood maltreatment as well as their level of PTSD contributed to the variance of childhood maltreatment reported by descendants (*R* ^2^=0.13, χ^2^=21.0, *p*<0.0001). Also, exposure to war and genocide, exposure to childhood maltreatment and maternal PTSD symptoms, but not paternal symptoms, explained the amount of depressive and anxious symptoms in descendants (*R* ^2^=0.40, χ^2^=76.4, *p*<0.0001). In those descendants who fulfilled the A criterion of the DSM-IV diagnosis for PTSD, the CTQ sum score in descendants was positively correlated with the PTSD sum score (*r*=0.24, *p*<0.05). And, descendants growing up with a parent suffering from PTSD reported more physical abuse throughout childhood than those without (*U*=2229.5, *p*<0.001). These findings add evidence to the existing literature postulating that childhood maltreatment might be an important agent in the transmission of psychopathology from parent to child. Issues of a “cycle of violence” and the specificity of maternal PTSD in Rwanda are discussed.

References

Kellerman, N. P. (2001). Psychopathology in children of Holocaust survivors: A review of the research literature. Israel Journal of *Psychiatry and Related Sciences, 38*, 36–46.

Yehuda, R., Halligan, S. L., & Grossman, R. (2001). Childhood trauma and riskfor PTSD: relationship to intergenerational effects of trauma, parental PTSD, and cortisol excretion. *Development and Psychopathology, 13*, 733–753.

Schaal, S., & Elbert, T. (2006). Ten years after the genocide: Trauma confrontation and post-traumatic stress in Rwandan adolescents. *Journal of Traumatic Stress, 19*, 1–11.

Foa, E. B., & Tolin, D. F. (2000). Comparison of the PTSD Symptom Scale–InterviewVersion and the Clinician-Administered PTSD Scale. *Journal of Traumatic Stress, 13*, 181–191.

Bernstein, D. P., Fink, L., Handelsman, L., Foote, J., Lovejoy, M., Wenzel, K., Sapereto, E., & Ruggiero, J. (1994). Initial reliability and validity of a new retrospective measure of child abuse and neglect. *American Journal of Psychiatry, 151*, 1132–1136.

Derogatis, L. R., Lipman, R. S., Rickels, K., Uhlenhuth, E. H., & Covi, L. (1974). The Hopkins Symptom Checklist (HSCL): A self-report symptom inventory. *Behavioral Science, 19*, 1–15.

### Mental health and subjective distress due to parental political imprisonment in adult offspring of former political prisoners of the GDR 10:30–10:45

#### M. Boehm^1^, G. Klinitzke^2^, E. Braehler^3^ and G. Weissflog^4^

^1^Department of Mental Health, Medical Psychology and Medical Sociology/LIFE Research Centre for Lifestyle Disease, University of Leipzig, Leipzig, Germany; ^2^Department of Mental Health, Clinic and Policlinic of Psychosomatic Medicine and Psychotherapy, University of Leipzig, Leipzig, Germany; ^3^Department of Mental Health, Medical Psychology and Medical Sociology, University of Leipzig, Leipzig, Germany; ^4^Department of Mental Health, Medical Psychology and Medical Sociology, Division of Psychooncology, University of Leipzig, Leipzig, Germany

*Background:* Studies on transgenerational traumatization suggest a multitude of relevant factors in the process of trauma transmission. We were interested in the association of different conditions with mental health and subjective perception of the distress resulting from parental imprisonment in a group of adult children of former political prisoners of the GDR. *Methods:* We compared different subsets of a sample of adult offspring of former political prisoners (*n=*64) to identify effects of primary vs. secondary traumatization related to parental imprisonment, maternal vs. paternal imprisonment and parental PTSD. Measures of mental health (PTSD symptoms: *PDS*, screening for mental health problems: *SCL-27*) and the subjective estimation of distress due to parental imprisonment (*visual analog scales*) were dependent variables. *Results:* With regard to mental health, no differences emerged comparing those who had witnessed the parental imprisonment with those who had not and comparing those who had both parents, only mother and only father imprisoned. Subjective psychological distress differed significantly, with witnessing the imprisonment leading to higher subjective psychological distress and significantly higher strain on family relationships and with imprisonment of father only leading to lowest subjective psychological distress, followed by imprisonment of mother only. The highest distress was reported by subjects who reported imprisonment of both parents. Subjects who had at least one parent with current PTSD reported significantly higher psychological symptom severity, but not PTSD symptom severity. Also, there were no significant differences between the groups regarding subjective distress. *Conclusions:* Contrary to our expectations, neither experiencing the parental imprisonment nor having both parents imprisoned led to higher psychological impairment in this sample. In line with other research, parental PTSD seems to be more relevant for their children's mental health. Altogether, the results point to a complex process between appraisal of aspects of the parental experience and mental health in the second generation.

### Transgenerational transfer of traumatic experiences and the role of rearing behavior in survivors of the Khmer Rouge regime and their children 10:45–11:00

#### N. Stammel^1^, S. Burchert^1^, K. Antonietti^2^ and C. Knaevelsrud^1^

^1^Center for Torture Victims, Free University Berlin, Berlin, Germany; ^2^Free University Berlin, Berlin, Germany

*Background:* The transgenerational transmission of traumatic experiences and their psychological consequences are increasingly being discussed in trauma research. Even though at present there is no conclusive evidence for the existence of a direct transmission of trauma, empirical data suggest that there might be an indirect transfer of traumatic experiences mediated by parental rearing behavior. The aim of this study is to examine the relationship between parental traumatization and the children's psychopathology by closely inspecting the potential mediation effects of parental rearing behavior in survivors of the Khmer Rouge regime and their offspring in Cambodia. *Method: N*=378 mother-child pairs were interviewed in a randomized cross-sectional study in four provinces of Cambodia. We assessed symptoms of posttraumatic stress disorder (PTSD), anxiety, and depression and different aspects of perceived parental rearing behavior in structured interviews. *Results:* Preliminary results did not show a significant relationship between maternal traumatic experiences and children's PTSD and no differences in children's PTSD between mothers with and without PTSD. However, there was a gender-specific moderating effect: the daughter's own traumatic exposure had a stronger effect on their PTSD symptoms the higher their mother's traumatic exposure was (*β*=0.18, *p*<0.05). Maternal PTSD did not significantly correlate with abusive (*r=*0.05; *p*=0.36), rejecting (*r*=0.06; *p*=0.24) or overprotective (*r*=0.00; *p*=0.95) rearing behavior. No indirect effects from maternal PTSD on children's PTSD children's anxiety and children's depression mediated through parenting behavior could be found via path analysis. *Discussion:* This study does not provide evidence for the existence of a direct transmission of trauma. However, there seems to be a gender-specific latent vulnerability for PTSD. There was no support for an indirect intergenerational effect mediated by parenting behavior.

## Invited Symposium: Intergenerational transmission of trauma and abuse

### First, second, and third generation effects of the Holocaust 11:45–12:00

#### M. J. Bakermans-Kranenburg^1^, M. H. van IJzendoorn^1^ and A. Sagi-Schwartz^2^

^1^Centre for Child and Family Studies, Leiden University, Leiden, The Netherlands; ^2^Center for the Study of Child Development, University of Haifa, Haifa, Israel

Of special interest to the study of intergenerational transmission of trauma and abuse is the case of the Holocaust. We conducted a series of both primary and meta-analytic studies on Holcaust survivors, including first, second, and third generations of survivors. With regard to the first generation, Holocaust survivors were meta-analytically compared with their counterparts (with no Holocaust background) on a range of outcomes, including psychological wellbeing, post-traumatic stress symptoms, psychopathological symptomatology, cognitive functioning, and stress-related physiology. Holocaust survivors showed substantially more post-traumatic stress symptoms, but showed remarkable resilience in several other domains of functioning (physical health, stress-related physical measures, and cognitive functioning). Concerning the second and third generation, no secondary traumatization effects were found. Secondary traumatization emerged only in studies with clinical participants, who were stressed for other reasons. Based on our own empirical work, we suggest that dissociation in the first generation may moderate the intergenerational transmission of dysregulated HPA-axis functioning. We examined the effects of the Holocaust on diurnal cortisol secretion in survivors and their adult offspring. Israeli female Holocaust survivors and matched comparisons formed a case-control study design with two generations: 32 Holocaust survivors and 33 comparisons, along with their offspring (total *n*=144). Holocaust survivors showed higher levels of daily cortisol versus comparisons. Their offspring showed lower cortisol levels when surviving parents displayed more dissociation.

### Neurobiology and attachment: a further perspective on trauma 12:00–12:15

#### M. Ammaniti and A. Speranza Sapienza Rome University, Italy

The recent contribution of neurobiological research in the area of attachment allows a wider understanding of the dynamics of traumatic experiences. A recent research has explored how different attachments (secure and dismissing) influence brain responses in areas related to empathy and emotions. It has been evidenced that dismissing subjects, who had infantile experiences of refusal toward emotional needs, activated motor, mirror, and limbic areas to a significantly greater extent, but deactivated the medial orbito frontal cortex (mOFC) and the perigenual anterior cingulated cortex (pACC). This brain hyperactivation may reflect emotional dysregulation connected to infantile experiences of rejection and lack of protection where as increased deactivation of fronto-medial areas may be the expression of the inhibition of attachment behaviors, which is a typical aspect of dismissing attachment. As regards other traumatic experiences, another study has explored neurobiological correlates of traumatic dissociation from an attachment perspective. Attachment disorganization related to early traumatic experiences is known as a powerful precursor of dissociative psychopathology in adulthood. Cortical connectivity modifications in EEG coherence were studied in subjects with dissociative disorders after attachment memories retrieval. Compared to healthy controls, who showed a significant increase in EEG connectivity, particularly in high-frequency EEG bands (beta and gamma) after retrieval of personal attachment-related autobiographical memories through the Adult Attachment Interview (AAI), patients showed a lack of modifications of EEG connectivity at each frequency band explored. These results shed light on the neurological bases of the dis-integrative effect of attachment disorganization in dissociative patients.

### Enhanced amygdala reactivity to emotional faces in adults reporting childhood emotional maltreatment 12:15--12:30

#### A. Van Harmelen^1^, M. Van Tol^2^, L. R. Demenescu^3^, N. J. A. Van Der Wee^4^, D. J. Veltman^5^, A. Aleman^6^, M. A. Van Buchem^7^, P. Spinhoven^8^, Brenda W. J. H. Penninx^4^ and B. M. Elzinga^8^

^1^Leiden University, Leiden, The Netherlands; ^2^BCN NeuroImaging Center, University of Groningen, Groningen, The Netherlands; ^3^Department of Psychiatry, Psychotherapy and Psychosomatics, RWTH AachenAachen, Germany; ^4^Department of Psychiatry, Leiden University Medical Center, The Netherlands; ^5^Department of Psychiatry, Graduate School for Neurosciences Amsterdam, Research Institute Neurosciences, Vrije Universiteit, Amsterdam, The Netherlands; ^6^Behavioural and Cognitive Neuroscience Neuroimaging Center, University Medical Center Groningen, Groningen, The Netherlands; ^7^Department of Radiology, Leiden University Medical Center, Leiden, The Netherlands; ^8^Institute of Psychology, Leiden University, Leiden, The Netherlands

In the context of chronic childhood emotional maltreatment (CEM; emotional abuse and/or neglect), adequately responding to facial expressions is an important skill. Over time, however, this adaptive response may lead to a persistent vigilance for emotional facial expressions. The amygdala plays a key role in face processing, however, until now, the neurobiological correlates of face processing in adults reporting CEM were unknown. Here, we found that healthy controls and unmedicated patients with depression and/or anxiety disorders reporting CEM before the age of 16 (*n*=60), showed enhanced bilateral amygdala reactivity to emotional (angry, fearful, sad, happy, and neutral) versus scrambled facial expressions when compared to controls and patients who report no childhood abuse (*n*=75). CEM related enhanced amygdala response to emotional faces in general was independent of individuals' psychiatric status. These findings may be key in understanding increased emotional sensitivity and interpersonal difficulties, that has been reported in individuals with a history of CEM.

### Probing social exchanges—a computational neuroscience approach to understand Borderline and Anti-Social Personality Disorder and the impact of attachment-related trauma 12:30--12:45

#### T. Nolte^1,2^, B. King-Casas^3,4^, J. Feigenbaum^5^, R. Montague^4,6^ and P. Fonagy^1^

^1^Research Department of Clinical, Educational and Health Psychology, University College London, London, UK; ^2^Developmental Neuroscience Unit, Anna Freud Centre, University College London, London, UK; ^3^Department of Neuroscience & Computational Psychiatry Unit, Baylor College of Medicine, Houston, USA; ^4^Menninger Department of Psychiatry and Behavioral Sciences Baylor College of Medicine, Houston, USA; ^5^Research Department of Clinical, Educational, and Health Psychology, University College London, UK; ^6^Human Neuroimaging Laboratory, Department of Neuroscience, Baylor College of Medicine, Houston, USA

Borderline Personality Disorder (BPD) and Anti-Social Personality (ASPD) disorder represent a common but often extremely debilitating form of severe psychopathology, often characterized by attachment disorganisation and early adversity. Functional neuroimaging research has shed light on the neural circuitry involved in complex mental processes such as affect regulation and social cognition or empathy, many of which are thought to be impaired in patients with BPD and ASPD. In our computational psychiatry framework, we used a number of two person social exchange paradigm as critical approximations to the interpersonal difficulties experienced by both BPD and ASPD patients to investigate shared and distinct computational processes and their underlying neural correlates. Preliminary behavioural and neurobiological data from 50 patients in this large scale study will be presented and linked with indices of patients' development such as attachment representations and relational trauma.

## Afternoon
*Keynote Address*

### A public health approach to understanding and preventing violent radicalization 14:00–15:00

#### K. Bhui Centre for Psychiatry, Wolfson Institute of Preventive Medicine, Barts & The London School of Medicine & Dentistry, London, UK

Very recent acts of terrorism in the UK were perpetrated by “homegrown”, well-educated young people, rather than by foreign Islamist groups; consequently, a process of violent radicalization was proposed to explain how ordinary people were recruited and persuaded to sacrifice their lives. Counterterrorism approaches grounded in the criminal justice system have not prevented violent radicalization. Indeed, there is some evidence that these approaches may have encouraged membership of radical groups by not recognizing Muslim communities as allies, citizens, victims of terrorism, and victims of discrimination, but only as suspect communities who were then further alienated. Informed by public health research and practice, a new approach is proposed to target populations vulnerable to recruitment, rather than rely only on research of well-known terrorist groups and individual perpetrators of terrorist acts. This lecture proposes public health research and practice to guard against violent radicalization.

## Invited Symposium: Cultural differences in the assessment of trauma II

### Religion, spirituality and coping with trauma 15:15–15:30

#### S. Dein Mental Health Sciences Unit, Faculty of Brain Sciences, UCL, London, UK

There is emerging literature demonstrating positive relationships between religiosity and mental health. More specifically, studies indicate that among religious individuals, positive religious coping has positive mental health benefits, whereas negative religious coping may adversely impact upon mental health. This lecture examines the religious response to trauma, both social and individual, how it facilitates coping, and the psychological response. I include natural disasters such as the tsunami and human-made disasters such as 9/11. I discuss how individuals use spiritual resources in dealing with such events. Finally, I propose how religious frameworks can be incorporated into therapy when working with those affected.

### Homicide and culture 15:30–15:45

#### A. Ajaz Fellow in Medical Education and Honorary Clinical Lecturer & Specialist Registrar in Forensic Psychiatry, London UK

According to the *Global Burden of Armed Violence* report (2nd edition, 2011), an estimated 526,000 people die violently every year, but only 55,000 of them lose their lives in conflict or as a result of terrorism. More specifically 396,000 people (including 66,000 women) are victims of intentional homicide (murder), 54,000 die as a result of so called ‘unintentional’ homicides (manslaughter), and 21,000 violent deaths occur during law enforcement actions. Homicide rates in the general population in England and Wales have been steadily increasing over the past three decades. The findings from the National Confidential Inquiry into Suicide and Homicide by People with Mental Illness (NCI) has shown that the number of homicides by people experiencing symptoms of mental illness has also increased. From 1997 to 2006, 5884 general population homicide convictionswere notified to the NCI. There was a significant rise in homicides in the general population; 2% per year, during this period. There was anoverall increase in the numbers of those with schizophrenia and those with psychotic symptoms at the time of the offence which was 4% per year over the study period. This rise was in part was associated with a rise in recent immigration in the psychotic cases, however it could not account sufficiently for the overall increase, which was more likely due to increased rates of co-morbid substance misuse. In this session a case will be presented looking at the relationship between homicide, mental illness and culture and there sultant challenges in assessment and diagnosis. The UK Criminal Justice System is reducing the number of successful partial defencesto homicide (i.e. diminished responsibility) because of a mental disorder, even though the number of mentally ill perpetrators of homicide has increased. This worrying trend suggests that considerations related to mental disorder are increasingly being overlooked and this is even more likely when it comes to considering important cultural factors.

### Acculturation stress in indigenous immigrants in Guadalajara, Mexico 15:45–16:00

#### S. J. Villaseor-Bayardo and M. P. Aceves Pulido Universidad de Guadalajara, Mexico

Diversity in Mexico can be appreciated in the number of indigenous languages spoken in the country. This diversity carries with it problems derived from coexistence and different worldviews. Such problems require an analysis from the perspective of those who face a process of integration as a minority into the dominant culture. The city in particular is a context where the phenomenon of acculturation stress is experienced by indigenous migrants every day. The adaptation strategy most commonly used by the migrant population is known as acculturation, a term adopted to account for the subject's interaction with his/her context by assimilating, separating, or excluding themselves from it. The term “acculturative stress” is used to highlight the phenomenon of migration and its psychological consequences. Inserting the idea of subjectivity and culture into the realm of health implies understanding the way in which its conception and imbalance relate to the discourse produced by culture and a system of ideas. Thus, it becomes necessary to understand the system of beliefs, values, and lifestyles that produce and reproduce that reality, and that have been subjectively assimilated by the indigenous population. This article aims to understand their experience with a qualitative approach. Their life stories allowed us to have access to the circumstances that led to their acculturative stress.

### Culture, post-natal depression, and trauma 16:00–16:15

#### A. Persaud^1,2^, K. Bhui^1,2^ and M. Younger^1^

^1^The Centre for Applied Research and Evaluation-International Foundation(Careif) Centre for Psychiatry, Barts and the London, Barts & The London School of Medicine & Dentistry, London, UK; ^2^World Association of Cultural Psychiatry (WACP), Rome, Italy

The lives of women, their children and their families around the important and eventful time of childbirth can be improved. Some non-western cultures have elaborate postpartum rituals that give status and importance to the new mother. Such rituals can increase self-esteem, decrease marital stress, and clarify social status. These special attentions in western cultures end quite abruptly after childbirth, with the focus of attention invariably transferred to the baby. The effects and consequences of perinatal mental illness are widespread, affecting the sufferers, their children, families and all who care for them. Their experiences vary depending on their personal circumstances, ability to access help, lifestyle, single-parent status, economic position, ability to work or access to transport particularly in rural areas, race and cultural disposition, racism, communication barriers and isolation. They cite feelings of not being accepted by the indigenous majority population, racism, and indifference from statutory services. Maternal mental health problems therefore pose a huge human, social, and economic burden and constitute a major public health challenge. Although the overall prevalence of mental disorders is similar in men and women, women's mental health requires special considerations in view of women's greater likelihood of suffering from depression and anxiety disorders and the impact of mental health problems on childbearing and childrearing.

References

Albrighl, A. (1993). Postpanum depression: an overview. *Journal of Counseling & Development, 71*, 316–320.

Persaud, A., Vellerman, R., & Templeton, L. (2000). Postnatal depression in women from the Black and Ethnic Minority communities in a Rural area. *Journal of Primary Care Mental Health, 3*, 13–15.

## Invited Symposium: Cultural differences in the assessment of trauma II

### Psychological distress following traumatic facial injury in an East London population 16:45–17:05

#### E. Rahtz Centre for Psychiatry, Barts and The London, Queen Mary's School of Medicine and Dentistry, London, UK

Patients who suffer facial trauma, whether from accidents, road traffic collisions, or interpersonal violence, face a range of psychosocial issues as a result of their injuries. Though standards of surgical treatment to restore function and appearance continue to improve, psychological problems may often be overlooked in busy wards and clinics. While distress is to be expected in the immediate aftermath of traumatic events, some patients continue to experience psychological distress. As well as high levels of acute stress disorder, facial trauma patients are also at risk of depression and, in the longer term, may develop post-traumatic stress disorder (PTSD) (Glynn et al., 2007). Changes in appearance can exacerbate the problems by constantly reminding patients of traumatic events. Depression and PTSD are frequently comorbid in trauma patients, and rates of PTSD can be higher among ethnic minority groups (Shih, & Schell, 2010). However, manifestations of PTSD can be culturally specific, and distressed patients sometimes refuse to participate in research because of fears about revisiting the experience. These psychological conditions are treatable, however, and the ability to identify those at risk would enable healthcare practitioners to provide important interventions. This study looks at the culturally diverse population attending an East London hospital.

References

Glynn, S. M., Shetty, V., Elliot-Brown, K., Leathers, R., Belin, T. R., & Wang, J. (2007). Chronic posttraumatic stress disorder after facial injury: a 1-year prospective cohort study. *The Journal of Trauma, 62*(2), 410–418, discussion 418.

Shih, R., & Schell, T. (2010). Prevalence of PTSD and major depression following trauma-center hospitalization. *The Journal of Trauma, 69*(6), 1560–1566.

### Pre- and post-migration risk factors for psychological problems among Somali immigrants. Prevalence and predictors of psychological problems among Somali refugees 17:05–17:25

#### N. Warfa Centre for Psychiatry, Barts and The London, Queen Mary's School of Medicine and Dentistry, London, UK

This literature on refugee studies highlights the mental health needs of people who are exposed to traumatic life events. Compared with other populations, refugee groups have higher rates of mental disorders including PTSD and depression. This presentation focuses on the prevalence and predictors of psychological problems among Somali refugees. Remarkably, after 20 years of civil war, the rate of PTSD among Somali immigrants living in the West is low, mostly less than 14%. This presentation explores the specific sociocultural factors that might have kept the overall PTSD level low among this group.

### Cancer and PTSD-prevalence of post-traumatic stress symptoms in head and neck cancer patients 17:25–17:45

#### F. Shiraz Centre for Psychiatry, Barts and The London, Queen Mary's School of Medicine and Dentistry, London, UK

There is increasing evidence that a proportion of head and neck cancer patients may develop psychological problems including PTSD, depression, anxiety, and poor quality of life (So, Chan, & Chan, 2012). These problems are often not recognized or treated and have a detrimental impact on patient survival and well-being (Oskam et al., 2010). Cancer differs markedly from other known PTSD stressors in that it is not a discrete, short-lived event. The cancer experience consists, instead, of a series of events beginning with cancer detection and diagnosis, then active medical and surgical treatment, and concluding with recovery and follow-up. Head and neck cancer is a particularly complex and distressing disease with 5-year survival rate of around 56%. Often, the treatment is extensive and affects basic everyday functioning, such as eating, breathing, and speaking (Weymuller et al., 2003). Many patients are distressed by visible permanent disfigurement and disruptions to their physical, social, and occupational functioning (Vartanian et al., 2004). We measured levels of (1) acute stress, (2) anxiety and depressive symptoms and (3) quality of life in a sample of 124 head and neck cancer patients in follow-up (i.e., post-diagnosis). Participants were administered the acute stress disorder (Bryant, Moulds, & Guthrie, 2000) questionnaire to assess ASD, hospital anxiety and depression scale to assess anxiety and depression symptoms (Zigmond & Snaith, 1983) and World Health Organisation Quality of Life Questionnaire, (Skevington & Lotfy, 2004, WHOQOL BREF) to measure the overall quality of life. The results showed elevated levels of psychological distress in head and neck cancer patients with a significant proportion experiencing high acute stress symptoms. In conclusion, our results show the importance of measuring psychological distress throughout the cancer experience as some patients will continue to experience high levels of emotional distress and unmet psychological needs.

References

Bryant, R. A., Moulds, M. L., & Guthrie, R. M. (2000). Acute stress disorder scale: A self-report measure of acute stress disorder. *Psychological Assessment, 12*(1), 61–68.

Oskam, I. M., Verdonck-de Leeuw, I. M., Aaronson, N. K., et al. (2010). Quality of life as predictor of survival: a prospective study on patients treated with combined surgery and radiotherapy for advanced oral and oropharyngeal cancer. *Radiotherapy and oncology: Journal of the European Society for Therapeutic Radiology and Oncology, 97*(2), 258–262.

Skevington, S. M., & Lotfy, M. O. K. (2004). The World Health Organization's WHOQOL-BREF quality of life assessment: Psychometric properties and results of the international field trial. A report from the WHOQOL group. *Quality of Life Research, 13*, 299–310.

So, W. K. W., Chan, R. J., Chan, D. N. S., et al. (2012) Quality-of-life among head and neck cancer survivors at one year after treatment—A systematic review. *European Journal of Cancer (Oxford, England: 1990), 48*(15), 2391–2408.

Vartanian, J. G., Carvalho, A. L., Yueh, B., et al. (2004). Long-term quality-of-life evaluation after head and neck cancer treatment in a developing country. *Archives of Otolaryngology Head and Neck Surgery, 130*(10), 1209–1213.

Weymuller, E. A., Yueh, B., Deleyiannis, F. W., et al. (2003). Quality of life in head and neck cancer. *The Laryngoscope, 110*(1), 5–7.

Zigmond, A. S., & Snaith, R. (1983). The hospital anxiety and depression scale. *Acta Psychiatrica Scandinavica, 67*(6), 361–370.

## ORAL, JUNE 7 B. PLENARY HALL

## Morning
*Panel: Clinical utility of proposed ICD-11 categories for disorders specifically associated with stress*

### Introduction to ICD-11 proposals

#### A. Maercker University of Zurich, Zurich, Switzerland

This 2-part panel addresses the proposals for the diagnoses of stress-related disorders put forward for inclusion in the 11th edition of the World Health Organisation's International Classification of Diseases (ICD-11). Among potentially far-reaching changes are a new distinction between PTSD and Complex PTSD, the introduction of a prolonged grief disorder, and changes to the conceptualisation of acute stress reaction and adjustment disorder. Members of the WHO committee will present the scientific basis for the proposals and discuss how they differ from DSM-5 as well as their practical implications for clinicians. Disorders specifically associated with stress (such as PTSD, prolonged grief disorder) must be differentiated from other mental disorders and from normal, self-limited stress responses. WHO is aware of concern about an overuse of certain stress-related diagnoses, especially among populations that have been exposed to a natural or human-made disaster. A tendency to focus on stress-associated diagnoses may be related to the appeal of the simple, external explanation for symptoms, which is suggested by names such as PTSD. ICD-11 proposals distinguish PTSD from complex PTSD with the latter following prolonged or multiple traumatization and exhibiting a particular symptom pattern. The American Psychiatric Association has decided against the inclusion of a separate diagnosis in DSM-5 but instead has expanded the conceptualization of PTSD to include additional aspects of disturbed emotionality and behavior. There is also significant controversy in the field about some existing or proposed categories that are seen as “milder”, such as adjustment disorder or prolonged grief disorder. Some have challenged the validity and utility of these categories. In general, to help countries to reduce disease burden associated with mental disorders, the classification system must be usable and useful for health care workers around the world. With ICD-11, there appears to be a unique opportunity to produce such a system. Part 1 will focus on PTSD, complex PTSD, and adjustment disorder. Part 2 will focus on prolonged grief disorder and acute stress reaction.

**Panellists Part 1:** Andreas Maercker, Chris Brewin, Marylene Cloitre.

**Panellists Part 2:** Richard Bryant, Lynne Jones, Simon Wessely, Mark van Ommeren, and Andreas Maercker.

### Diagnosing PTSD from three core elements 8:45–9:10

#### C. Brewin University College London, London, UK

The proposed definition of PTSD for ICD-11 diverges markedly from the direction proposed for DSM-V. In ICD-10, the PTSD diagnosis already differs from DSM-IV in not having a formal stressor criterion and in placing greater weight on re-experiencing in flashbacks and nightmares. The new proposal retains these elements while making the diagnosis simpler and more systematic. PTSD is defined as consisting of three core elements: (1) re-experiencing: vivid intrusive memories, flashbacks, or nightmares that involve re-experiencing in the present, accompanied by fear or horror; (2) avoidance: marked internal avoidance of thoughts and memories or external avoidance of activities or situations reminiscent of the traumatic event(s); (3) hyperarousal: a state of perceived current threat in the form of hypervigilance or an enhanced startle reaction. The symptoms must also last for several weeks and interfere with normal functioning. In DSM-V, there will be over 8,000 different combinations of symptoms that can yield a diagnosis of PTSD: in ICD-11 there will be only 27 possible combinations of symptoms.

### The clinical utility of the ICD-11 complex PTSD diagnosis 9:10–9:35

#### M. Cloitre National Center for PTSD Palo Alto VA Health Care System, Palo Alto, CA, USA

This presentation will describe the rationale for and clinical utility of two related diagnoses, PTSD and complex PTSD within the spectrum of trauma and stress-related disorders. Pilot data will be presented regarding the validity of these two distinct classes of PTSD patients: differences in association with type of stressor and differences in severity of impairment. A latent profile analysis (LPA) conducted on 302 treatment-seeking individuals revealed three classes of patients: (1) complex PTSD patients who were high on PTSD symptoms as well as on disturbances in three domains of self-organization: affective dysregulation, negative self-concept, and interpersonal difficulties; (2) PTSD patients who were high on PTSD symptoms but low on the three self-organization symptom domains; and (3) a group of patients who were low on all symptoms. Chronic trauma was more strongly predictive of the complex PTSD than PTSD while conversely single-event trauma (9/11) was more strongly predictive of PTSD. In addition, complex PTSD was associated with greater impairment where significant contributions were made by the affective, self-concept, and interpersonal disturbance over and above PTSD symptoms. Individuals with borderline personality disorder (BPD) were eliminated from all analyses, suggesting that the complex PTSD class represents an identifiable group of individuals distinct from those with BPD. Treatment implications are discussed.

### Prolonged grief disorder 10:00–10:25

#### R. Bryant School of Psychology, University of New South Wales, Sydney, Australia

There is increasing evidence that approximately 10% of the bereaved people experience persistent grief reactions that are characterized by yearning for the deceased, which is accompanied by marked emotional pain. Across cultures, many studies have noted that severe grief reactions that persist beyond 6 months after the loss are predictive of mental health impairment, suicidality, poor health behaviors (e.g., smoking, alcohol abuse), and cardiovascular and immunological disease. ICD-11 is introducing for the first time a diagnosis to recognize this condition: prolonged grief disorder. It is defined as severe grief reactions that persist beyond 6 months after the death, and can be accompanied by disbelief, bitterness, a sense of emptiness, and loss of identity. A major motivation for this development is the hope that it will minimize inappropriate treatment of grief reactions and facilitate identification of people who can benefit from treatments that have been shown to be specifically useful for prolonged grief responses.

### Acute stress reaction: a new approach 10:25–10:50

#### L. Jones Center for Health and Human Rights, Harvard School of Public Health, Harvard University, Cambridge, MA, USA

Acute stress reaction (ASR) as it is currently defined in ICD 10 is ambiguous. The description of normative and *transient* emotional, cognitive, and behavioral reactions that subside within days following exposure to traumatic events is contradicted by its position in the F codes which labels it as pathology. The confusion is compounded by the parallel existence of acute stress disorder in DSM-IV. In practice, ASR often appears to be used interchangeably with adjustment disorders (Isserlin, Zerach, & Solomon, 2008). This presentation will (1) clarify the new definition, which places less emphasis on particular symptoms such as fugue states; (2) explain the importance of a temporal distinction from other stress disorders such as adjustment disorder and PTSD; and (3) give the rationale for moving ASR to the ICD-11 chapter containing categories that represent reasons for clinical encounters that are not themselves disorders or diseases (the “Z” chapter in ICD-10). For example in conflict and disaster situations, the new categorization will allow humanitarian and other agencies to provide immediate social and psychological assistance to those in need without unnecessarily pathologizing their experiences. Second, in the aftermath of acute traumatic events, it will allow access to short-term support from health systems that require a diagnostic code, again without pathologizing the reaction itself (World Health Organization, 2011).

References

Isserlin, L., Zerach, G., Solomon, Z. (2008). Acute stress responses: A review and synthesis of ASD, ASR, and CSR. *American Journal of Orthopsychiatry*, *78*, 423–429.

World Health Organization. (2011). *Psychological first aid: Guide for field workers*. Geneva: WHO.

## The spectrum of trauma-related disorders
*Symposium: Complicated grief: Progress in research on epidemiology and treatment*

### Complicated grief and PTSD in family-members after witnessing assisted suicide in Switzerland: social acknowledgement and forensic investigations as predictors 11:45–12:00

#### B. Wagner^1^, V. Boucsein^2^ and A. Maercker^2^

^1^Medical University, Leipzig, Leipzig, Germany; ^2^University of Zurich, Zurich, Switzerland

*Background:* Assisted suicide is permitted in only a few countries worldwide. However, few studies have examined the impact that witnessing assisted suicide has on the mental health of family-members or close friends and related risk factors. *Method:* A cross-sectional survey of 85 family-members or close friends who were present at an assisted suicide was conducted in December 2007. Full or partial posttraumatic distress disorder (PTSD) (impact of event scale-revised), depression and anxiety symptoms (brief symptom inventory), and complicated grief (CG) (inventory of complicated grief) were assessed at 14-24-month post-loss. *Results:* Of the 85 participants, 13% met the criteria for full PTSD (cut-off ≥35), 6.5% met the criteria for sub-threshold PTSD (cut-off ≥25), and 4.9% met the criteria for CG. The prevalence of depression was 16%; and the prevalence of anxiety was 6%. The diagnosis of PTSD disorder is significantly related to having experienced the forensic investigation as emotionally difficult. Further, social acknowledgement as a survivor was related to PTSD symptoms and CG. In particular, perceived general disapproval was strongly correlated with all outcome measures. *Conclusion:* A higher prevalence of PTSD and depression was found in the present sample than has been reported for the Swiss population, in general. However, the prevalence of CG in the sample was comparable to that reported for the general Swiss population. It is recommended that a protocol be developed establishing a standardized response to cases of assisted suicide and that specific training be provided for the legal professionals involved.

### Rates and risks for complicated grief in an orphaned sample 15 years after the Rwandan genocide 12:00–12:15

#### J. Unterhitzenberger and R. Rosner Catholic University Eichstaett-Ingolstadt, Eichsttt, Germany

Complicated Grief (CG) became a well-researched syndrome in adults in western countries during the last years. Still, only few studies report its prevalence in adolescent samples or third world countries. We present findings about the mental health in adolescents 15 years after the Rwandan genocide, which left around 300,000 children and adolescents orphaned. Adolescents (*N=*69) aged between 14 and 18 years (*M=*16.3, *SD=*1.17) living in rural Rwanda were given a newly developed self-report questionnaire on CG (grief questionnaire for children and adolescents, GQ-CA) and a questionnaire on major depression (MD) (Mini International Neuropsychiatric Interview, MINI). All participants (48% female) were bereaved by at least one parent and recruited through an orphanage and a secondary school. The results show that 76.8% of parents died due to genocide in 1994 with 50.7% of participants being double orphans. The high majority of deceased (72.6%) was murdered. Totally, 49.3% of adolescents (*N*=34) were screened positive for CG in our sample. Predictors for higher risk of CG were living with relatives, loss of both parents and meeting criteria for MD. Comorbidity with MD was high with 76.5%. Nevertheless, 23.5% of adolescents assessed met criteria for CG without meeting those for depression. The prevalence of CG in Rwanda remains high even with losses dating back more than 14 years and it is higher than reported in previous studies in the country. Risk factors for CG were identified. Even though we found high comorbidity rates with MD, the study indicates the distinctiveness of CG by means of participants with CG diagnosis only. Limiting factors-such as the sample size, the first use of the GQ-CA, and the sample's selectivity-are to be discussed.

### Posttraumatic growth and therapeutic alliance in a controlled clinical trial for the treatment of complicated grief 12:15–12:30

#### R. Rosner^1^, G. Pfoh^2^ and M. Kotoucova^2^

^1^KU Eichstaett-Ingolstadt, Eichsttt, Germany; ^2^LMU, Munich, Germany

*Aims:* The aim of this presentation is to look at posttraumatic growth and therapeutic alliance in a trial on cognitive behavioral treatment for patients diagnosed with comorbid complicated grief (CBT-CG). *Method:* Fifty-one patients were randomized to either a waiting list control group or CBT-CG. Assessment included the prolonged grief disorder interview (PG-13), the computer version of a structured interview for DSM-IV (DIA-X), the symptom checklist (SCL-90-R), the helping alliance questionnaire (HAQ), and the posttraumatic growth inventory (PTGI). *Results:* Posttraumatic growth improved only minimally after therapy. A mediating effect for posttraumatic growth was not found. CBT-CG reduced CG symptoms and this change influenced posttraumatic growth. Results concerning the therapeutic alliance were ambiguous and showed varying results, depending on when in therapy and whether the patient's or the therapist's perspective was used. Correlations between therapeutic alliance and symptom reduction were larger at the end of treatment. *Discussion:* Given the lack of comparable results regarding posttraumatic growth as treatment outcome, it may be premature to judge posttraumatic growth as a non-suitable construct to measure treatment outcome in trauma-related disorders. Maybe other forms of treatment than CBT-for example, more humanistic approaches-would find different results. Results concerning the therapeutic alliance are in line with studies on other disorders than CG.

### How do bereaved suffering from prolonged grief benefit from grief group participation? 12:30–12:45

#### K. Dyregrov^1^, I. Johnsen^2^ and A. Dyregrov^2^

^1^Center for Crisis Psychology/Norwegian Institute of Public Health, Oslo, Norway; ^2^Center for Crisis Psychology, Oslo, Norway

Although most people recover quickly and naturally after the loss of a loved one, research clearly demonstrates that a sizeable number of bereaved are at risk of developing more complex grief reactions and struggle to adapt. Many such bereaved are referred for assistance through grief groups. This paper will report from part of a study conducted to explore grief group participation after traumatic deaths in Norway. Participants who fulfilled the criteria of prolonged grief disorder (PGD) were compared with participants who did not in order to explore whether they differed on satisfaction and experiences with participation. To allow for comparison, a subsample of 22 participants who fulfilled the criteria of PGD was drawn from the total of 262 participants. Demographic and loss-related variables were analyzed to explore factors associated with PGD. Fulfillment of PGD was then analyzed to explore the effect on life quality and overall satisfaction. The main finding was that participants who fulfill the criteria of prolonged grief were in general less satisfied with the groups and reported less positive effect on life quality. These findings highlight the need for certain qualifications of group leaders and possibly pregroup screening of potential grief group participants.

## Panel: Mental health policy and trauma-informed services

### Mental health policy and trauma-informed services 14:00–15:15

#### V. Ardino PSSRU Unit, London School of Economics and Political Science, UK

Mental health has often been described as a Cinderella service. Yet, it is clear that even more remains to be done for trauma-informed services including a priority status attached to the recognition of trauma burden in statements of policies on health and social care. There remain more gaps in provision and shortfalls in the quality of care compared with other disorders. There also remain concerns about the “economic case” for trauma-informed services, particularly relating an efficient allocation of resources between different groups of traumatized individuals and between different forms of service provision. The panelists will reflect interactively with the audience on the challenges for trauma-informed services to create a dynamic balance between improving the mental well-being of traumatized individuals, preventing and treating post-traumatic consequences and to promote a meaningful continuum between these goals and effective policy strategies. Furthermore, they will bring up discussion on quality assurance to reflect the way in which the voice of victims of trauma has been co-opted in the development of effective services. In particular, patient safety and quality improvement (im-plementation, change management, indicators, multidisciplinary processes, redesign processes) will be discussed.

**Panelists:** Chris Freeman, Ruth Lanius, Erica Van der Schrieck-de Loos, and Vittoria Ardino

## Afternoon Responding to disasters
*Invited Symposium: European initiative on mass disasters*

### Psychosocial interventions in the aftermath of disasters: views of EFPA Standing Committee for crises, trauma, and disasters 15:15–15:35

#### N. Karanci Middle East Technical University, Turkey

EFPA established first a working party and then a Standing Committee on disaster, crisis, and trauma psychology to work on how psychology can contribute to preparing for and responding to emergencies and disasters. The Committee has been evaluating evidence and views from across Europe, has published a lessons learned document to reflect the experiences from various European disasters, worked closely with the European Commission, and embarked on further training for members from recently acceded countries. Training needs and quality standards for various professionals involved in psychosocial support is also an important area of our work. The Committee has also evaluated guidelines for the delivery of psychosocial support and target groups of trainees that are likely to be involved in psychosocial support services. Another important area is the process of collaboration in the case of cross-border disasters and incidents. Due to the extensive experiences of the members of our standing committee, we have made contributions in congresses and have worked in facilitating the formation of trauma and disaster psychology units in member countries. The presentation will provide an overview of the work of the standing committee and related recommendations.

### Protecting children in emergency contexts 15:35–15:55

#### V. Neri Save the Children, Italy

In the past few years, Italy has been affected by two major earthquakes that challenged psychosocial interventions for child victims of such natural disasters. The aim of this presentation is to provide an overview of the response of *Save the Children* Italy to the 2009 earthquake in Abruzzo and the strategic reasoning used following this to improve *Save the Children*'s ability to respond to national emergencies and the 2012 earthquake in Emilia Romagna. The presentation will discuss advocacy strategies for supporting children in mass-emergencies. *Save the Children* has a long tradition of program development for child protection in emergency contexts, both internationally and nationally. In particular, the creation of Child Friendly Spaces-safe areas where children can play, socialize, and begin to recover during emergencies-is an effective tool to support children according to the principles and approaches contained in the UN Convention on the Rights of the Child and to apply the five priorities for protection as defined in the document “Child Protection in Emergencies: Priorities, Principles and Practices”. In Emilia Romagna, *Save the Children*'s emergency staff immediately reached affected areas to assess children and adolescent needs and implement an intervention plan. This further demonstrated the importance of supporting children with psychosocial and educational interventions through a variety of activities and programs. *Save the Children* Italy is now coordinating a group of experts to define a set of guidelines to protect children in emergencies in Italy and is advocating for this at a governmental level.

References

Save the Children International. (2009). Reducing risks, saving lives.

Save the Children Italia Onlus. (2009). Decalogo per il supportopsicologico ai bambini, from www.savethechildren.it

Save the Children Italia Onlus. (2012). Come essere vicini ai nostri figli durante e dopo un'emergenza, from www.savethechildren.it

### The European Network for Traumatic Stress (TENTS) 15:55–16:15

#### J. Bisson Cardiff University, Wales, UK

The European Union (EU) funded the European Network for Traumatic Stress (TENTS) project between 2007 and 2009. TENTS established a community-wide network of expertise on posttraumatic stress treatment for victims of natural and other disasters, examined which interventions are effective in the aftermath of disaster and whether these are available throughout Europe. TENTS produced guidelines and an evidence-based model of care along with dissemination materials. The European Network for Traumatic Stress-Training & Practice (TENTS-TP), funded by the EU between 2009 and 2011, expanded and developed the network that currently includes 36 European countries. It also connected other important European initiatives in the field of psychosocial care after trauma. The TENTS model of care was combined with guidance produced by NATO to develop a curriculum and teaching materials to disseminate and implement evidence-based care to those affected by traumatic events throughout Europe. Professionals responsible for teaching and training in this field were identified, provided with an evidence-based teaching package, and equipped to implement this in a sustainable manner. This has resulted in levels of knowledge and expertise of mental health and social service professionals being raised, which should result in improved services to those affected by traumatic events. During this presentation, the development of the TENTS guidelines and the TENTS-TP Train the Trainers package will be described along with the ongoing work of TENTS since 2011. Please visit www.tentsproject.eu and http://www.healthplanning.co.uk/portfolio for more information.

## Evidence-based practice on trauma
*Symposium: Continuing to implement evidence-based trauma treatments: Lessons learned over the long-term*

### Community application and evaluation of alternatives for families: a cognitive-behavioral therapy 16:45–17:00

#### D. Kolko Western Psychiatric Institute and Clinic, Pittsburgh, Pennsylvania, PA, USA

This presentation provides an overview of the community application and evaluation of Alternatives for Families: A Cognitive-Behavioral Therapy (AF-CBT; www.afcbt.org), a treatment approach for family conflict, physical coercion/abuse, and child behavior problems that is administered in three phases (engagement, individual skills-building, and family applications). AF-CBT has been implemented in diverse settings (e.g., outpatient, in-home, foster care), by different practitioners (BA-level caseworkers and MA-level clinicians) and with a variety of families having child welfare or mental health involvement. Training approaches recently used to teach practitioners the model will be described, notably, learning collaboratives and learning communities, to highlight the different levels or domains that are targeted prior to, during, and after a staff training program. We will then review data collected from various training programs to identify and address key implementation issues and challenges, including delivery of treatment with fidelity, family engagement, and sustainability. We will specifically review lessons learned about the sequencing of content and participants (child, caregiver, and joint sessions), use of brief treatment modules, and the need to apply creative clinical strategies designed to address and overcome negative reactions in the therapy (e.g., hostility and callousness, abuse minimization, challenging clinician authority, dismissiveness, aggressive gestures/threats). Lessons learned from supervisory and senior leadership (management) training will also be discussed, including the need to address enrollment, confidentiality, vicarious trauma, safety policies, and ongoing quality assurance methods. Finally, we will identify potential solutions to overcoming barriers to conducting AF-CBT to help practitioners incorporate this approach, such as specialized engagement methods, rapid assessment tools to identify clinical targets, psychoeducation and motivational enhancement routines, and safety monitoring strategies, agency metrics, and homework practice assignments, motivation for change, engagement, and active participation in treatment.

### Sustainment of EBP in low-resource public mental health 17:00–17:15

#### L. Berliner University of Washington, Seattle, WA, USA

Implementation science has taught a number of lessons for implementation and sustainment of EBP. However, many of the methods for high-quality implementation and sustainment activities require external funding or significant infusion of additional resources. Many “brand name” EBTs require adopting organizations to purchase their support and monitoring programs. However, most brand name EBTs target a single outcome that can create organizational complications and costs for managing all of the sustainment activities across treatments. The harborview EBP initiative encompasses general or non-brand controlled interventions for the primary mental health conditions for which children present for care (e.g., posttraumatic stress, depression, anxiety, behavior problems). The initiative is designed for public mental health agencies that serve multi-problem children and families in complex psychosocial circumstances (e.g., foster care). It has government support for some of the key initial implementation activities (initial organizational consultation, training, case consultation, and ongoing consultation to clinical supervisors). It does not provide support for internal organizational sustainment activities. This presentation will describe a variety of practical organizational strategies that promote sustainment including approaches to incorporating routine standardized assessment to identify a clinical target and promote treatment engagement, establishing ongoing evidence-based internal supervision and access to internal training for new staff. Fidelity to the EBP model has been identified as critical for achieving outcomes, yet monitoring fidelity is a high-cost activity. This presentation will outline a number of quality assurance approaches that can be undertaken within the existing resources and that can applied across EBTs.

### Implementing an evidence-based method for treating traumatized youth in regular clinics: experiences from Norway 17:15–17:30

#### T. Jensen Norwegian Centre for Violence and Traumatic Stress Studies, Oslo, Norway

After the July 22, 2011 terror attack in Norway, the Heath Directorate initiated a nationwide plan for implementing trauma-focused cognitive behavioral therapy in child mental health clinics throughout Norway. Implementing evidence-supported interventions poses several challenges at the professional and organizational levels. Often mentioned obstacles are related to transferring models from a controlled, academic environment into ordinary clinics. Children referred to regular clinical care settings may be different from children treated in specialized university clinics, including severity of symptoms, family support, and motivation for change. We have limited knowledge as to how this affects the delivery of treatment. Moreover, many traumatized children and families referred for treatment in community settings are not seeking treatment for trauma-specific symptoms. Even in cases where the trauma history is acknowledged, children often are referred for other problems, such as depression or externalizing behavior. Implementation may include introduction of new assessment procedures at the clinics. The working conditions of therapists may also differ. In regular clinics, therapists have to treat a broad variety of disorders and few are specially trained in trauma treatment methods. Learning a new model while seeing other patients or having other demanding tasks may influence how therapists learn and deliver an intervention. Therapist turnover requires systems of implementation that ensures sustainability of the model. In this presentation, experiences from an implementation model will be presented based on the following seven core components: staff recruitment, preservice training, ongoing consultation, staff performance evaluation, decision support data systems, administration support, and system intervention. Focus will be on challenges that were encountered at the profesional and organizational levels. These challenges and solutions will be analyzed in light of data from an effectiveness study conducted in Norway.

### Six years of EBT implementation: lessons learned from Project BEST 17:30–17:45

#### B. Saunders Medical University of South Carolina, Charleston, SC, USA

Project BEST is a statewide effort to implement TF-CBT using a community-based learning collaborative approach throughout the state of South Carolina (US) to ensure that every abused and traumatized child who needs it will receive effective, evidence-based treatment. This presentation will include empirical data concerning the impact of Project BEST and qualitative and anecdotal descriptions of key lessons learned as this project has matured. Now in its 6th year, more than 500 broker and clinical professionals have been trained and currently are using TF-CBT in 39 or the 46 counties. Prepost treatment effect sizes for PTSD symptoms for child clinical training cases averaged *d*=1.16, larger than prepost effects found in the recent clinical trials of TF-CBT. Overall outcome matrices (73.1% improved by >1/2 SD) and diagnostic results (36–11% meeting PTSD criteria) also showed significant improvements. Similar results were found for measures of depression. These results indicate that community service providers can achieve excellent results using an evidence-based trauma treatment. However, implementation efforts have encountered frequent obstacles at the community, organization, and personal levels. Solutions have been tried and some been found to be successful. Lessons learned at each of these levels will be described. These include the importance of: (1) local, committed leadership; (2) brokers to the community implementation of a clinical treatment; (3) training brokers in their roles and responsibilities for treatment outcomes; (4) a sense of shared community responsibility and collective, community problem solving; (5) interorganizational relationships; (6) measuring clinical outcomes; (7) measuring implementation markers; and (8) use of these metrics by service organizations to facilitate service delivery. Implications for future implementation projects and research will be discussed.

## ORAL, JUNE 7 HALL AUDREY GRACE

## Morning
*Debate: Collective traumas: how to remember, how to heal*

### Collective traumas: how to remember, how to heal 10:00–11:00

#### P. Violi^1^, J. Halpern^2^, C. Demaria^1^ and D. Salerno^1^

^1^TRAME: Centre for the Interdisciplinary Study of Cultural Memory and Traumas, University of Bologna, Bologna, Italy; ^2^Institute for Disaster Mental Health, State University of New York, USA

The panel aims to open an interdisciplinary dialogue between psychological and cultural approaches to the theme of collective trauma. Collective traumas are more than the simple sum of many individual traumatic events; a collective trauma affects the entire life of a society, leaving often indelible traces in the physical environment, destroying houses and cities, and even more importantly, the ways people remember their experiences and attribute meaning to them. Memory lies at the core of any reflection on collective trauma: how can a society live with the memory of a terrible past? How can people deal with their traumatic past, especially in the case of conflicts, where memories involved are always conflicting and in opposition to one another? These and similar issues need to be examined through a range of disciplinary approaches. One panelist will describe a project where US trauma specialists worked in partnership with Middle East practitioners to develop a series of psycho-educational materials designed to expand awareness of the effects of disasters and chronic violence for children, caregivers, helpers, and the general public. Other participants in the panel, all working within a framework of cultural semiotics and memory studies, will discuss different aspects of the relationship between cultural trauma and memory.

**Chairs:** P. Violi and J. Halpern

**Panellists:** P. Violi, J. Halpern, C. Demaria, and D. Salerno

## Evidence-based practice on trauma
*Symposium: Trauma-focused cognitive-behavioral therapy (TF-CBT): research, training, and dissemination updates*

### Research on Trauma-Focused Cognitive Behavioral Therapy for Children and Families 11:45–12:00

#### J. Cohen Allegheny General Hospital, Pittsburgh, PA, USA

Trauma-Focused Cognitive Behavioral Therapy (TF-CBT) is a components- and phase-based treatment for traumatized children and adolescents and their parents or other caretaking adults (Cohen JA, et al. 2006; Cohen JA, et al. 2012). More than a dozen randomized controlled treatment trials (RCT) have evaluated the effectiveness of TF-CBT for traumatized children ages 3-17 years old related to diverse index traumas including sexual abuse, domestic violence, multiple/complex traumas, disaster and war (Cohen JA, et al. 2010). These studies have documented that TF-CBT consistently improves children's Posttraumatic Stress Disorder (PTSD) symptoms significantly more than comparison or control conditions. They also document that a variety of other child symptoms improve significantly more with TF-CBT than with comparison or control treatments, including depressive, anxiety, and behavioral symptoms, shame, and trauma-related maladaptive cognitions. TF-CBT is also significantly more effective than comparison conditions for improving parental emotional distress, parental support, effective parenting strategies and parental PTSD symptoms. TF-CBT has been evaluated cross-culturally and found to be effective in diverse populations. For example, RCTs respectively for sexually abused children in Australia; for multiply traumatized children across eight Norwegian community mental health clinics, and for sexually exploited war-exposed girls in the Democratic Republic of Congo, have documented that TF-CBT was significantly more effective in improving children's PTSD and other mental health symptoms than comparison or control conditions for these children. Additional ongoing RCT studies are currently ongoing in several other countries.

References

Cohen, J. A., Mannarino, A. P. & Deblinger, E. (2006). *Treating trauma and traumatic grief in children and adolescents:* New York: Guilford Press.

Cohen, J. A., Mannarino, A. P. & Deblinger, E. (Editors) (2012). *Trauma-focused CBT for children and adolescents: Treatment applications*. New York: Guilford Press.

Cohen, J. A., Mannarino, A. P. & Deblinger, E. (2010). Trauma-focused cognitive behavioral therapy for traumatized children; In Weisz, J. R. & Kazdin, A. E., Eds. *Evidence-based psychotherapies for children and adolescents, 2nd Edition*. New York: Guilford Press, pp. 295-311.

### Online resources for TF-CBT training and implementation 12:00–12:15

#### B. Saunders Medical University of South Carolina, Charleston, SC, USA

Implementation of evidence-based trauma treatments has reached the scale-up stage in many countries. Service systems are attempting to train thousands of service providers effectively and implement and sustain new practices with reasonable levels of fidelity in hundreds of service organizations. Many organizations are now struggling with how to train new therapists that replaced previously trained ones. It is unlikely that traditional approaches to professional continuing education can meet this demand, making the use of technology necessary. This presentation will describe several online resources for mental health professionals who are learning TF-CBT and how they can be used to help meet these significant training and implementation challenges. TF-CBTWeb is a free, multi-media 10-hour online training course that teaches the fundamental components and techniques of TF-CBT. TF-CBTWeb has over 144,000 registered learners worldwide, over 73,000 (51%) of whom have completed the full course. Median time of completion is 11 days and 80% of learners complete it within 6 weeks. Most learners (74%) hold masters degrees, and nearly two-thirds have less than 5 years of experience. Clinical social workers and professional counselor comprise 68% of all learners. Evaluation data indicate significant pre to post knowledge gains and high learner satisfaction with the course. CTGWeb is a 6-hour follow-up online course that teaches therapists how to apply TF-CBT to cases of child traumatic grief. TF-CBTConsult is an automated, multimedia online clinical consultation website that provides answers to the most common questions asked by new therapists learning TF-CBT. TF-CBTConsult currently has over 60 answer pages for common problems. Each of these resources will be described and demonstrated. How best to use these resources in training and implementation projects on TF-CBT will be described.

### TTF-CBT Train-the-Trainer Programs 12:15–12:30

#### A. Mannarino Allegheny General Hospital, Pittsburgh, PA, USA

Trauma-Focused Cognitive-Behavioral Therapy (TF-CBT) has been studied extensively over the past 20 years and has the most empirical support of any treatment for children and families exposed to traumatic life events. The demand from child mental health organizations for TF-CBT training has increased dramatically as county and state governments and other jurisdictions have begun to mandate that evidence-based treatments (EBTs) be provided. In order to meet this demand, the TF-CBT developers have developed a national Train-the-Trainer Program. This TTT Program and its parameters will be discussed in this presentation. To date, there have been three cohorts of TF-CBT trainers and there are now over 50 approved trainers. This has resulted in over 400 TF-CBT trainings and over 15,000 therapists being trained in the years 2009–2011. Additionally, there has been increasing demand for TF-CBT training in Europe. Building on existing collaborations, the TF-CBT developers have created a TF-CBT International Train-the-Trainer Program which includes Norway, Sweden, The Netherlands, and Germany. The International TTT Program will be described in this presentation as well as extensive training efforts in other countries such as Japan.

### Dissemination and sustainment of TF-CBT in community mental health 12:30–12:45

#### A. Mannarino Allegheny General Hospital, Pittsburgh, PA, USA

Trauma-Focused Cognitive-Behavioral Therapy (TF-CBT) has been studied extensively over the past 20 years and has the most empirical support of any treatment for children and families exposed to traumatic life events. The demand from child mental health organizations for TF-CBT training has increased dramatically as county and state governments and other jurisdictions have begun to mandate that evidence-based treatments (EBTs) be provided. In order to meet this demand, the TF-CBT developers have developed a national Train-the-Trainer Program. This TTT Program and its parameters will be discussed in this presentation. To date, there have been three cohorts of TF-CBT trainers and there are now over 50 approved trainers. This has resulted in over 400 TF-CBT trainings and over 15,000 therapists being trained in the years 2009–2011. Additionally, there has been increasing demand for TF-CBT training in Europe. Building on existing collaborations, the TF-CBT developers have created a TF-CBT International Train-the-Trainer Program which includes Norway, Sweden, The Netherlands, and Germany. The International TTT Program will be described in this presentation as well as extensive training efforts in other countries such as Japan.

## Afternoon
*Symposium: Complex trauma and psychopathology SISST-SIPGES*

### Memories of attachment hampers cortical connectivity in dissociative patients 15:15–15:35

#### B. Farina^1,2^
Centro dipartimentale di formazione integrale, Universitá Europea di Roma, Rome, Italy; ^2^Centro Clinico de Sanctis, Rome, Italy

The presentation will show the results of an experiment where we evaluated cortical connectivity modifications by EEG coherence analysis in subjects with dissociative disorders after attachment memories retrieval. According to many scholars, memories related to a traumatic attachment may trigger dissociative processes when the attachment motivational system is activated in the adult life by hampering the integrative mental functions. But, in our knowledge, no any neuroscientific evidence supported this hypothesis. We aimed to track the status of high order integrative mental functions in dissociative patients compared to controls by means of EEG cortical coherence after the Adult Attachment Interview, that it is supposed to be an optimal trigger of attachment memories. Results of the experiment and their possible outcome for the clinical work will be discussed.

### Alexithymia and addiction behaviours: does trauma play a role? 15:55–16:15

#### V. Caretti Dipartimento di Psicologia, Universitá di Palermo, Palermo, Italy

This paper overviews the role of trauma in addiction behaviours and the intersection with alexithymia. There will be a summary of many studies conducted by the group leaded by V. Caretti on the topic of substance misuse and trauma with interesting data on dissociation, alexithymia. Furthermore the work underscores the importance of assessment of trauma in such populations.

## ORAL, JUNE 7 HALL DIAMANTE

## Morning
*Open Papers: Evidence-based practice I*

### Traumatic incident reduction: novel evidence-based resolution techniques for psychological trauma 10:00–10:15

#### J. Durkin Institute of Mental Health, Nottingham, UK

Traumatic Incident Reduction (TIR) is a recent addition to the evidence-based approaches to trauma listed by the Substance Abuse and Mental Health Services Administration in the USA. Built on established theoretical foundations and taking a broadly person-centered perspective, its techniques predict rapid and positive changes in a relatively short amount of therapeutic time. Early empirical data will be presented for evidence of trauma resolution and posttraumatic growth, some in a single session, in community samples from the USA, Canada, and UK. As TIR can be delivered by lay practitioners, the potential for its use in communities unable to access psychologic therapies will be discussed.

### Trauma-focused treatment for PTSD during the asylum process: application of the phased model 10:15–10:30

#### E. Walsh Traumatic Stress Clinic, Camden and Islington NHS Mental Health Foundation Trust, London, UK

The recommended model of treatment for PTSD (NICE, 2005) for refugees and asylum seekers following chronic trauma is based on Herman's (1992) phased model of treatment. Herman outlines three phases of treatment, namely 1) stabilization/building a sense of safety and trust, 2) trauma-focused treatment/remembrance, and 3) re-integration. It is recommended that the second phase, trauma-focused treatment, is not approached until a client feels safe and secure in their current environment. It is well-documented that there have been marked delays in the UK asylum process in recent years. Many people seeking asylum have had to wait over 5 years for their case to be heard, while having to manage fears in relation to possible deportation and practical difficulties of living within the asylum system. This raises a dilemma for therapists attempting to ensure appropriate clinical intervention for clients with PTSD, and not wishing to delay treatment that can offer symptomatic relief. Trauma-focused CBT is a recommended intervention for PTSD. Recent studies have focused on using trauma-focused CBT for refugees (Grey and Young, 2008). This presentation will use clinical case material to discuss trauma-focused treatment with clients with PTSD to human rights abuses in their country of origin, the country to which they would be deported should their claim be refused. It will also consider safety and symptom management during the wait for resolution of asylum claims, including negotiating the requirements of the immigration authorities.

References

Grey, N., & Young, K. (2008). Cognitive behaviour therapy for refugees and asylum seekers experiencing traumatic stress symptoms. *Behavioural and Cognitive Psychotherapy*, *36*, 3–19.

Herman, J. (1992). Trauma and recovery. Basic Books, New York.

National Institute for Clinical Excellence (NICE). (2005). The Management of PTSD in Primary and Secondary Care. Full Guideline. *National Collaborating Centre for Mental Health*.

### Trauma-focused cognitive-behavioral therapy (TF-CBT) for youth with complex trauma 10:30–10:45

#### A. Mannarino and J. Cohen Allegheny General Hospital, Pittsburgh, PA, USA

Trauma-Focused Cognitive-Behavioral Therapy (TF-CBT) has been studied extensively over the past two decades, including in at least a dozen randomized clinical trials. Research has demonstrated that TF-CBT is an effective treatment intervention for youth aged 3-16 who have been exposed to sexual abuse, domestic violence, traumatic loss, and multiple traumas. These studies have shown that TF-CBT helps to remediate PTSD symptoms, depression, anxiety, shame, behavior problems, and parental distress in youth with trauma exposure. Over the past five years, the authors have acquired extensive experience in implementing TF-CBT with youth with complex trauma presentations and in consulting with clinicians both nationally and internationally who are treating youth with chronic trauma histories. This presentation will describe the implementation of TF-CBT with youth with complex trauma. There will be a discussion of TF-CBT as a “phase-based” treatment, including 1) an initial focus on safety and trust given these youngsters' backgrounds; 2) dedicating proportionally more of the model to the TF-CBT coping skills phase; 3) titrating gradual exposure more slowly as needed by individual youth; and 4) incorporating unifying trauma themes (e.g., feeling damaged; shame; sense of responsibility, etc.) throughout treatment. A composite clinical case will be included in the presentation to illustrate how TF-CBT can be implemented with youth with complex trauma backgrounds.

### Transcending trauma: effectiveness of transcendental meditation practice for combating PTSD 10:45–11:00

#### F. Travis Maharishi University of Management, Fairfield, IA, USA

PTSD results from a natural response to an unnatural situation. The natural response is amygdala activation during highly emotional experiences to ensure deeper processing. The unnatural situation is extreme trauma. Traumatic experiences turn on amygdala functioning and keep it elevated-you want to be sure to avoid that situation again. This presentation reports the effect of transcending trauma through meditation practices on PTSD symptoms. The focus will be on effects of Transcendental Meditation (TM) practice, a meditation in the Automatic Self-Transcending Category. Results of three studies will be reported. First, a random assignment study of 18 Vietnam veterans reporting that TM was more effective than psychotherapy in reducing anxiety, depression, insomnia, alcohol abuse, PTS symptoms, and stress reactivity (Brooks & Scarano, 1985). A second single-group study of veterans from Iraq and Afghanistan reported significant reductions in anxiety, depression, and PTS symptoms after three-months practice (Rosenthal et al., 2011). A third matched study of 40 Congolese refugees with PTSD reported reduction in PTSD symptoms in the TM group after 30-days TM practice, which remained low at 135-days. PTSD symptoms in the control group trended upward (Rees et al., in press). This presentation will explore possible mechanism underlying significant reductions in PTSD symptoms through TM practice. It will include a model of how stress and transcending effect brain processing.

References

Brooks, J. S., & Scarano, T. (1985). Transcendental meditation in the treatment of post-Vietnam adjustment. *Journal of Counseling and Development, 64*, 212–215.

Rees, B., Travis, F., Shapiro, D., & Chant, R. (in press). Reduction in posttraumatic stress symptoms in Congolese refugees practicing transcendental meditation. *Journal of Traumatic Stress*.

Rosenthal, J. Z., Grosswald, S., Ross, R., & Rosenthal, N. (2011). Effects of transcendental meditation in veterans of operation enduring freedom and operation Iraqi freedom with posttraumatic stress disorder: a pilot study. *Military Medicine, 176*(6), 626–630.

### The SAMIFO Center to care torture victims 11:00–11:15

#### G. Santone^1^, F. Gnolfo^1^ and M. R. Silvestri^2^

^1^ASL ROMA A—ROMA, Italy; ^2^ASL ROMA—A, Italy

Forced migrants, escaped from their countries because of politic, ethnic, religious, or gender problems, are not comparable to economic migrants in terms of health. In fact, forced migrants are highly exposed to psychic, social and physical hazard in their destination countries. The healthcare dedicated to asylum seekers and refugees must be conceived through a systemic approach multidisciplinary and multidimensional at the same time. The local health net GRIS Lazio permitted Public Health Care and Private Social Assistance to confront and share their ideas of good practice in order to create common resources, developing common ways of reflection on critical areas in the matter of migrants health. The agreement protocol (decisions ASL Roma A N°260–March 31, 2006 and N°1001 July 27, 2010) between ASL RM A and Centro Astalli association officially founded SAMIFO health center: a multidisciplinary integrated system between Public Health Care (ASL Roma A) and Private Social Assistance (Italian Jesuit Refugees Service).

**Aims**

1) To promote and facilitate the fruition to public health care

2) To inform patients about their rights and about the related information sources

3) To educate healthcare professionals about migration medicine topics

4) To ensure cultural-linguistic mediation to overcome the barrier of language and intercultural communication

5) To promote systemic approach to multidimensional trauma.

**Scientific committee**

1) Goals and actions planning

2) Promotion of information research and education activities

3) Raise the awareness of social and institutional actors

4) Promotion of the circulation of knowledge and results

5) Definition of a list of evaluation indicators

In our centre, in 2012, there have been more than 1,200 new outpatients and main health visits have been more than 5,039 general medicine, 1,002 psychiatric, 930 gynecologic, 263 forensic medicine, 532 psychologic. Total visits 7,766.

## Open Papers: Evidence-based practice II

### Morbidity following extreme trauma exposure 11:45–12:00

#### T. Lundin Department of Neuroscience, Uppsala University, Uppsala, Sweden

One way of describing the health status in a population is in terms of the frequency of days of sickness per year. With the help of data on the registered days of sickness per year, a study was made of a group of Swedish tsunami survivors (n=1221) who were non-bereaved and extremely exposed during the 2004 tsunami disaster in Southeast Asia. In order to investigate whether the extreme trauma exposure had influenced registered sickness a period of four years was studied, from two years before the trauma until two years after. In all cases, the “background sickness” was eliminated through taking into account only the increase in days of registered sickness per year. Student's t-test was used for statistical analysis. We found a significantly increased morbidity.

### Reducing flashbacks with somatic updates within trauma focused cognitive behavior therapy 12:00–12:15

#### J. Gratton Traumatic Stress Clinic, London, UK

Traumatic events can lead to a wide range of psychologic and social sequelae for the survivor (Herman, 1992). Some survivors experience flashbacks as part of constellation of symptoms recognized as posttraumatic stress disorder. There are many types of flashbacks; such as visual, auditory, olfactory, and somatic. Somatic flashbacks can involve internal sensations such as pain or nausea. It can also involve movement: restricted, such as being held down; and forced, such as falling. Psychological interventions can come from multiple theoretical perspectives. The most commonly used intervention for specific symptoms of flashbacks is trauma focused cognitive behavior therapy (tf-cbt) (Grey et al., 2002). The use of hotspot updates such as cognitive or image-based updates is particularly well-described for visual flashbacks. However, there is a lack of literature on updating somatic flashbacks especially when cognitive updates are not effective. A brief review of the literature is followed by specific case examples related to traumatic events during child sexual abuse, torture, female genital mutilation, and domestic violence.

References

Grey, N., Young, K., & Holmes, E. (2002). Cognitive restructuring within reliving: A treatment for peritraumatic emotional “hotspots” in posttraumatic stress disorder. *Behavioural and Cognitive Psychotherapy*, *30*(01), 37–56.

Herman, J. (1997). Trauma and recovery: The aftermath of violence from domestic abuse to political terror. (Previous Ed: 1992). London: Basic Books.

### Recovery scan: the routine use of a brief online screening instrument for PTSD at an early stage combined with telephone counseling (coaching exposure in vivo) and/or EMDR as an early therapeutic intervention 12:15–12:30

#### S. Berendsen^1^ and J. Gouweloos^2^

^1^Institute for Psychotrauma (IVP), Diemen, The Netherlands; ^2^Impact, the Dutch knowledge & advice centre for post-disaster psychosocial care, Amsterdam, The Netherlands

Organizations are both morally and legally obliged to provide optimal psychosocial care for employers confronted with traumatic incidents at work. Therefore, various organizations expressed the need for tools that help them to provide this. To meet their needs, the IVP developed the “Recovery Scan”: an online tool to monitor the recovery process of employers. The recovery scan consists of the Impact of Events Scale and questions on daily functioning and social support. Although the NICE guideline on PTSD (2005) recommends to start using a screening instrument 1 month after a traumatic incident, the recovery scan is offered after 1–2 weeks. The scan results in a traffic light score (green, orange, or red) gives an indication of how the employer is recovering. First results show that 1–2 weeks after the traumatic incident a majority (65%) scores “green” (no severe posttraumatic stress symptoms), indicating a high level of self-healing capacity. When employers score “orange” or “red” (possible difficulties with recovering), the IVP calls the employer for an initial telephone intervention. This consists of psycho-education, strengthening coping recourses, and coaching exposure in vivo techniques to diminish avoidance reactions. In accordance with the NICE guideline, EMDR is offered to those with severe posttraumatic stress symptoms in the first month after the traumatic incident. The Dutch railway system uses the Recovery Scan for 2 years. Since May 2012, more then 180 train divers and tickets collectors gave permission to use their results for research. This presentation elaborates on the practical use of the scan and the evaluation of the users. Furthermore, it will show first results on the level of stress reactions shortly after a traumatic incident at work, the recovery processes and treatment needs.

### Narrative exposure therapy for the treatment of complex PTSD: an examination of the effect and adaptation in a Japanese clinical setting 12:30–12:45

#### I. Domen^1^, M. Ejiri^2^ and S. Mori^3^

^1^Division of Clinical Psychology, Sawa Hospital, Osaka/Department of Human Sciences, Konan University, Kobe, Japan; ^2^Department of Psychiatry, Sawa Hospital/Hokuto Clinic Hospital, Osaka, Japan; ^3^Department of Human Sciences, Konan University, Kobe, Japan

*Background*: Narrative exposure therapy (NET) is a short-term intervention based on cognitive-behavioral therapy and testimony therapy. NET has been developed for treating complex PTSD caused by organized violence and mainly has been applied to victims of war and torture. There are few published studies of NET as applied to PTSD patients with trauma histories other than war-related traumas. Neither case studies, trial reports, nor RCTs of NET have been reported yet in Japan, though application of KIDNET to abused children in child-care institutions has been started. *Aim of the study*: A pilot study was employed to examine the adaptation of NET to patients diagnosed with complex PTSD living in safe life-conditions in Japan, and its effect. *Method*: Five patients (all females, from 20s to 40s) with PTSD symptoms, recruited from an out-patient clinic and from a university counseling room, received three to four months of NET treatment (60–120 min, once to twice a week) and supplemental counseling. Two of them were diagnosed with depression and one was diagnosed with BPD. Types of trauma included childhood abuse with attachment trauma, domestic violence and witness of DV, and traffic accidents, etc. The symptoms were measured by CAPS, IES-R, SDS, and DES until a year later. *Results*: Assessments showed a significant reduction in symptoms of PTSD, dissociation, and guilt. Depression symptoms, as assessed by SDS, however, did not decrease significantly. Habituation and reintegration of autobiographical memory, together with cognitive restructuring, are thought to have reduced patients' PTSD symptoms and improved their interpersonal relations and social functioning. Subjects' narrative, behavioral, and physical symptoms were changed positively. NET was applicable even to subjects with symptoms of dissociation, dissociated memories sense, and emotions. *Conclusions*: The results indicate that NET could be an effective method for treating complex PTSD patients in Japan.

### Evaluating a sexual violence therapy group for incarcerated women: symptom change and therapeutic alliance 12:45–13:00

#### M. Karlsson^1^, A. Bridges^1^, L. Milner^1^, J. Bell^2^ and P. Petretic^1^

^1^University of Arkansas, Fayetteville, USA; ^2^Peace at Home Family Shelter Inc., Springdale, USA

Incarcerated women report higher rates of sexual victimization than the average woman (Severson, Postmus, & Berry, 2005). One in three incarcerated women suffers from PTSD (Teplin, Abram, & McClelland, 1996). Researchers have suggested that sexual victimization is a pathway to prison for women and that there is a need for gender specific treatments (Bloom, Owen, & Covington, 2004) such as trauma treatments that targets sexual victimization and associated issues. This study is an evaluation of a brief (8 sessions) sexual violence therapy group for incarcerated women adapted from already established cognitive-behavioral treatments. Outcomes from five groups (N=39) show participants experienced significant decreases in PTSD, depression, anxiety, and worry. All effect sizes are large. This presentation will focus on the relation between symptom change from pre-to post-treatment and therapeutic alliance (*n*=19). Controlling for demographics, there was a trend for therapeutic alliance to predict greater change in depressive symptoms (*p*=0.09; R^2^=0.44). Moreover, symptom severity at pre-treatment (*p*<0.05) and greater therapeutic alliance (*p*=0.08) predicted greater change in depressive symptoms (R^2^=0.39). Therapeutic alliance was rated high (negatively skewed with low variability) and had no significant impact on changes in PTSD, anxiety, and worry symptoms over the course of treatment. Implications for trauma treatments, with a focus on incarcerated women will be discussed.

References

Bloom, B., Owen, B., & Covington, S. (2004). Women offenders and the gendered effects of public policy. *Review of Policy Research, 21*, 31–48. doi: 10.1111/j.1541-1338.2004.00056.x

Severson, M., Postmus, J. L., & Berry, M. (2005). Incarcerated women: Consequences and contributions of victimization and intervention. *International Journal of Prisoner Health, 1*, 223–240. doi: 10.1080/17449200600554611

Teplin, L. A., Abram, K. M., & McClelland, G. M. (1996). Prevalence of psychiatric disorders among incarcerated women I: Pretrial jail detainees. *Archives of General Psychiatry, 53*, 505–512.

## Afternoon
*Open Papers: Evidence-based practice III*

### What research on factors influencing trauma responses can tell us about survivors' vulnerability to later stress and trauma 15:15–15:30

#### E. Carlson US National Center for PTSD, VA Palo Alto Health Care System, CA, USA

A large number of factors affect responses to trauma. These factors include characteristics of individuals (such as genetic or biological tendencies, developmental level and experiences, past trauma exposure, life stress at the time of the event, and gender), aspects of traumatic stressors (such as severity), posttraumatic symptoms, and posttraumatic life experiences and resources (such as social support, financial resources, treatment, and posttraumatic life stress). These variables typically work in combination, and their relative influences vary across individuals. Given such variation in the vulnerabilities of trauma survivors whom clinicians treat and the likelihood that these clients will be exposed later in life to highly stressful and traumatic events, it can be valuable to consider which variables might make a particular client more vulnerable to the *next* highly stressful life event or traumatic event he or she experiences. This presentation will first briefly review past research on pretrauma, time of trauma, and posttrauma variables that have been found to be significantly related to PTSD, noting prospective studies and meta-analysis studies. The presentation will then describe the results of a longitudinal study of recent survivors of traumatic injury of self or a family member. We examined the relationship of pretrauma variables, time of trauma variables, and posttrauma variables to PTSD and depression at two months and one year posttrauma. Results showed that the variables assessed accounted for 73% of the variance in PTSD symptoms at 2 months and 90% at one year and 65% of the variance in depression symptoms at 2 months and 84% at one year. Individual differences in risk profiles for those who developed PTSD and homogeneity in risk profiles for those who recovered well will also be presented, and we will consider which variables that make trauma survivors more vulnerable can most readily be addressed clinically.

### Assessing and addressing survivors' unique risk profiles to foster resilience 15:30–15:45

#### E. Carlson US National Center for PTSD, VA Palo Alto Health Care System, CA, USA

There are important individual differences in the variables that make trauma survivors vulnerable to later stressful life events and later traumatic stressors. In addition to treating trauma survivors presenting and prominent posttraumatic psychological symptoms, assessing these variables and addressing them with clients may increase survivors' resilience. This presentation will review major risk factors for posttraumatic psychological disorder including individual characteristics (such as genetic or biological tendencies, developmental level and experiences, past trauma exposure, life stress at the time of the event, and gender), aspects of traumatic stressors (such as severity), posttraumatic symptoms, and posttraumatic life experiences and resources (such as social support, financial resources, treatment, and posttraumatic life stress). We will then discuss how most of these variables can be assessed to identify an individual trauma survivor's unique vulnerabilities to later life stress and trauma. Clinically relevant and freely available measures will presented for past trauma exposure, life stress, trauma severity, PTSD, dissociation, depression, cognitive/emotional expectancies, posttraumatic cognitions (including negative cognitions about self, negative cognitions about the world, and self-blame), affective sensitivity, self-destructive behaviors, social support, social constraints, and emotion approach coping/avoidance. Using measures like these, therapists can identify a unique risk profile for each trauma survivor that will reflect the survivor's greatest vulnerabilities and can then target these vulnerabilities to reduce or minimize them. Some empirically-supported options for addressing these potential vulnerabilities will be reviewed, including dialectical ehavior therapy, acceptance and commitment therapy, skills training in affect and interpersonal regulation (STAIR), and mentalizing therapy. A brief intervention that specifically addresses social support and social constraints and a mobile phone application aimed at fostering recovery from traumatic stress will also be described.

### Early interventions designed to help victims of a violent crime: a systematic literature review of psychological outcomes 15:45–16:00

#### S. Guay^1^, E. De Tournay-Jett^2^, D. Beaulieu-Prvost^3^ and A. Marchand^3^

^1^School of Criminology, University of Montreal, Montreal, Canada; ^2^Trauma Study Centre, Fernand-Seguin Research Centre, University of Montreal, Montreal, Canada; ^3^University of Quebec, Montreal, Canada

Criminal acts are the most common traumatic events to which the general population is exposed. Developing clinical guidelines for preventing PTSD among crime victims would help to reduce mental and overall health costs. The goal of the present article was to systematically review published studies on the efficacy of early interventions for crime victims. From the 10 studies that were selected, five evaluated the efficacy of cognitive-behavior therapy (CBT), four evaluated psychological debriefing (PD) and one evaluated other types of interventions (i.e., a video). Our review found modest and inconsistent effects of active early interventions. CBT appeared to be the most promising intervention when compared to a control group or a progressive relaxation group, but relatively equivalent to supportive counseling. No proof of efficacy was found for PD when compared to other interventions or a control group, even though delayed PD and critical incident stress management (CISM) appeared to be superior to early PD and critical incident stress debriefing (CISD), respectively. A psychoeducational video for rape victims appeared to help a sub-group of victims (i.e., those who had been previously raped). The size of the reduction in PTSD symptoms for the assessment (or control) condition and PD are quite similar (*r* between 0.36 and 0.45) while the one for CBT is approximately twice as much (*r* between 0.78 and 0.82). The confidence intervals of the differences confirm that the symptoms reduction for CBT is statistically larger than for PD. Most studies did not evaluate the impact of the interventions on variables other than anxiety and depressive symptoms. Further research is needed in order to develop early interventions to prevent PTSD, improve quality of life, and reduce costs for health care.

### Change during the course of therapy: results from a randomized controlled trial 16:00–16:15

#### J. Diehle, F. Boer and R. Lindauer American Medical Center–de Bascule, Amsterdam, The Netherlands

Children who are exposed to a traumatic event are at risk to develop a posttraumatic stress disorder (PTSD) or related problems and co-morbid disorders. Trauma-focused cognitive behavioral therapy (TF-CBT) and eye movement desensitization and reprocessing (EMDR) are advised by the NICE guidelines for the treatment of PTSD in children. We included 48 children in a randomized controlled trial comparing 8 sessions of TF-CBT and 8 sessions of EMDR. Investigation of treatment outcome on the clinician administered PTSD scale for children and adolescents (CAPS-CA) revealed no differences in effect sizes between the two therapies. It has often been argued that EMDR treatment shows faster results for the reduction of PTSD symptoms, especially re-experiencing symptoms. For the elaboration of this hypothesis, we included measures of PTSD during the course of therapy. Before therapy, at sessions 2, 4, and 6 and after therapy, children filled out the children's revised impact of event scale 13 items version (CRIES-13). Data for the change during the course of therapy will be presented and discussed in the light of treatment effectiveness and efficiency.

### Factor analytic structure of the impact of events scale-revised: 16:15–16:30

#### S. Wagner and C. Waters University of Northern British Columbia, Prince George, Canada

*Purpose*: To evaluate the factor structure of the IES-R when used with a volunteer firefighter and a similar community participant sample. *Methodology*: A volunteer firefighter sample (*n*=65) and a sample of similar community respondents (*n*=103) completed a questionnaire study, including responses to the IES-R. The IES-R data from both groups were entered into a three-factor principal components analysis with direct oblimin rotation. *Findings*: We found further support for the validity of the IES-R when used with a community sample. However, our data suggest that when using the IES-R with a community sample, the choice between a two- and a three-factor model may depend on the composition of the participants. For volunteer firefighters, the factor analytic structure of the IES-R appeared to be similar to that of the community sample, with more scatter in terms of item loadings. *Originality/value*: To our knowledge, there is no previous research considering the use of the IES-R with a strictly volunteer firefighting sample. In addition, despite adequate research on the factor analytic structure of the original IES, little research has considered the factor analytic structure of the more recent IES-R, even with community samples.

## Open Papers: Family processes

### Traumatic experiences, anxiety, dissociation and attachment styles in substance abuse patients 16:45–17:00

#### V. Chimienti Dipartmento di Scienze dell'Uomo, Universitá degli Studi di Urbino “Carlo Bo”, Urbino, Italy

Trauma in the early experiences of life is clinically associated with maladaptive development of relational functions and adult patterns of behavior. In particular, subjects with traumatic experiences present symptoms connected to disruption of normally integrated flow of consciousness. Anxiety, dissociation, emotion dis-regulation, and affect avoiding are the core features of complex posttraumatic stress disorder. In many cases, substance abuse can represent an economic way to self-treat this condition. The aim of this research explores reported traumatic experiences in drug addiction sample and relation between trauma, dissociation, anxiety, and attachment styles. This research was conducted on 102 Italian outpatients with drug addiction diagnosis. All subjects were administered a battery of self-report questionnaires: traumatic experiences checklist, dissociative experiences scale II, creative experiences questionnaire, state-trait anxiety scale and attachment style questionnaire. High frequency of traumatic experiences was found, especially in female and polydrug abusers. Emotional neglect, emotional abuse, and body threat severity composite scores were modestly correlated to dissociative experiences, trait and state anxiety, and fantasy proneness. Comfort and preoccupation with relationship are related to majority of reported trauma. No association between trauma and dismissing style of attachment was found. Substance abusers report many and different kind of traumatic experiences in their life. Traumatic experiences confirm a direct influence on activation of alert system, as showed by association with anxious traits, dissociation tendencies and preoccupied style of attachment. In many cases justifying self-medication hypothesis of drug assumption for complex posttraumatic condition.

### Traumatic experiences among men seeking treatment for using intimate partner violence 17:00–17:15

#### B. Loemo, I. R. Askeland, J. Strandmoen, T. Heir and O. A. Tjersland Norwegian Centre for Violence and Traumatic Stress Studies, University of Oslo, Oslo, Norway

*Aims*: Several studies report high prevalence of traumatic experience among men using intimate partner violence (IPV). However, there has been little tradition for integrating trauma work in IPV treatment. The aim of this presentation is to present data on prevalence of potentially traumatic experiences and violent behavior among men using IPV and its implications for treatment. *Methods*: Traumatic experiences checklist (TEC) and a questionnaire on violent behavior (VQ) was administered in a pretreatment clinical interview of 192 men who voluntarily attended treatment for IPV. *Results*: The majority of the men reported high numbers of potential traumatic experiences, especially in their family of origin. Half of the men had experienced emotional neglect (49.2%). Equal numbers were found for emotional abuse (48.4%) and six out of ten (61.8%) had experienced physical abuse from their parents or older siblings. Nearly seven out of ten (65.6%) men reported physical violence against partner during the year prior to assessment. Psychological violence were reported by nearly eight out of ten (78.1%) men. Traumatic experiences in family of origin were associated with the extent of violent behavior. *Discussion*: The high prevalence of traumatic experiences reported in this group of men and the association between trauma experiences and violence indicate that in addition to being a behavioral problem, IPV can be understood as a trauma-related disorder. Further, the results indicate the need to assess for traumatic experience and its possible trajectories in relation to violent behavior. A single case will be present to illustrate how to work trauma focused within the frame of IPV treatment.

### Secondary traumatization and post-traumatic growth among former prisoners of war wives: the moderating role of self-differentiation and empathy 17:15–17:30

#### G. Zerach^1^ and Z. Solomon^2^

^1^Department of Behavioral Sciences, Ariel University Center, Ariel, Israel; ^2^Bob Shapell School of Social Work, Tel Aviv University, Tel-Aviv, Israel

*Objectives*: Psychic trauma can impact the traumatized individual's significant others, a phenomenon known as secondary traumatization (ST). However, the indirect exposure to trauma might also be accompanied with positive psychological changes, termed posttraumatic growth (PTG). Whereas war captivity is known as highly traumatogenic experience, only few studies focused on its pathogenic and salutogenic impact on ex-prisoners of war (ex-POWs) spouses present study examined ST and PTG among ex-POWs wives in comparison to wives of a matched control veterans. Moreover, this study further explores the contribution of two psychological mechanisms-i.e., self-differentiation and empathy-to wives' ST and PTG. *Methods*: Israeli ex-POWs' wives (*N*=116) and a matched control group of wives of combat veterans (N=56) were assessed using a variety of self-report measures during 2011. *Results*: It was found that ex-POWs' wives reported higher levels of ST and perception of husband's posttraumatic symptoms (PTSS) and lower levels of PTG compared to control wives. It was also found that the more husbands' PTSS and the higher wives' levels of fusion self-differentiation, the higher their reported ST. We found that empathy for distress moderated the relationship between husbands' PTSS and wives' ST; interaction of high husbands' PTSS and wives high empathy for distress was related to higher ST. In addition, we found both husbands' PTSS and wives' ST positively contribute to wives' PTG. Last, we found that cut-off self-differentiation moderated the relationship between wives' ST and PTG; interaction of wives' low ST, and low levels of cut-off was related to low PTG. *Conclusion*: The experience of living with former ex-POWs who might also suffer from PTSS is associated with wives' own distress but also positive outlook on life. Both self-differentiation and empathy might serve as a buffer against the toll of ST and advance PTG experiences.

### Reliving childhood trauma in couple relationship and resolution in relational marriage therapy 17:30–17:45

#### S. Jerebic and D. Jerebic Family Institute Blizina, Celje, Slovenia

Childhood sexual abuse is a traumatic event that occurs in a relationship, and it is connected to problems in adult interpersonal relationships, especially couple relationships. Clinical experience shows that some survivors of sexual abuse experience the majority of flashbacks that reminds them of their original sexual abuse through sexuality. The relived and intrusive symptoms, thus, cause the individual to lose touch with the present and respond as if the trauma was taking place now. The involuntary memories are accompanied by physical and emotional distress that does not only hurt the victim but also their spouse who might experience symptoms of traumatization. Thus, couples often seek help from marriage and family therapists. The submission will present a therapeutic model of relational marriage therapy that uses the couple relationship to resolve trauma. The relational marriage paradigm assumes that the spouses regulate their internal psychological pain by projecting them onto each other through various emotional, cognitive, and behavioral patterns. Through transfer of projective-introjective identification one partner at least temporarily frees themselves from their pain; however, the other partner carries the pain instead. Using counter-transfer, the therapist, thus, discovers the internal psychological contents of both partners, addresses and names them, and helps both partners solidify new relationship patterns. This submission will, therefore, help broaden the understanding of the consequences of childhood sexual abuse trauma in relation to the intimacy of a couple relationship and contribute to practical knowledge and efficient use of the relational marriage therapy process in processing trauma.

### Relationship between psychic structure, adult attachment and symptoms on war trauma 17:45–18:00

#### P. Ferrajao and R. Aragao Instituto Superior de Psicologia Aplicada, Lisbon, Portugal

*Introduction*: Literature highlights the protective role of both a secure adult attachment style and a better organization of psychic structure following exposure to a traumatic event. Both psychological processes promote the ability of emotional regulation and integration of traumatic experiences and the mobilization of social support. However, the relationship between attachment style adult and psychic structure is not clearly established in research. *Objectives*: Study of the relationship between the level of integration of psychic structure and adult attachment behavio, and correlation with clinical symptoms after exposure to a traumatic event. *Measures*: Operationalized psychodynamic diagnosis OPD-2; experiences in close relationships; brief symptom inventory. *Method*: Participants, war veterans exposed to traumatic events, performed two interviews that were recorded and transcribed. Two independent raters analyzed the interviews to assess Axis IV (structure) of the OPD-2. Participants completed two self-report instruments: experiences in close relationships; brief symptom inventory. We tested the role of the level of integration of psychic structure as a moderating variable in the relation between adult attachment style and clinical symptoms after exposure to a traumatic event. *Results*: The results indicate that the level of integration of psychic structure is a moderating variable intervening in the relationship between adult attachment style and clinical symptoms after exposure to a traumatic event, verifying that a secure attachment style is a protective factor against exposure to a traumatic event in participants who presented a higher level of integration of psychic structure. *Conclusion*: The results highlight the protective role of the psychic structure of the organization by intervening in mobilizing external resources and emotion regulation skills following exposure to a traumatic event.

## ORAL, JUNE 7 HALL FALCO

## Morning
*Invited Symposium: Neurobiology of dissociation*

### Influence of dissociation on emotional and cognitive processing in interpersonally traumatized patients with borderline personality disorder 10:00–10:15

#### A. Krause-Utz^1^, N. Y. L. Oei^2,3^, P. Spinhoven^2,4,5^, M. Bohus^1^, C. Schmahl^1,*^ and B. M. Elzinga^2,4,*^

(*equally contributed authors)
^1^Department of Psychosomatic Medicine and Psychotherapy, Central Institute of Mental Health, Mannheim, Germany; ^2^Leiden Institute for Brain and Cognition, Leiden University Medical Center, Leiden, The Netherlands; ^3^Department of Gerontology and Geriatrics, Leiden University Medical Center, Leiden, The Netherlands; ^4^Institute of Psychology, Clinical Health and Neuropsychology Unit, Leiden University, Leiden, The Netherlands; ^5^Department of Psychiatry, Leiden University Medical Center, Leiden, The Netherlands

*Objective*: Emotion dysregulation is a core feature in borderline personality disorder (BPD). Functional magnetic resonance imaging (fMRI) studies have revealed a hyperreactivity of the amygdala and insula during emotion challenge in BPD patients compared to healthy participants (HC). Emotional distress is often associated with dissociative experiences in BPD. It has been proposed that dissociation is characterized by an overmodulation of affect associated with an inhibition of limbic brain activation. We aimed to investigate the influence of dissociative states on emotional distractibility in BPD patients. *Methods*: In a first study, we included 22 unmedicated BPD patients with a history of interpersonal traumatization and 22 HC (matched for age and education). During fMRI, participants performed a working memory task, while being distracted by negatively arousing versus neutral pictures from the International Affective Picture System. Before and after the task, participants completed the Dissociation Stress Scale (DSS-4), a measure of state dissociation. Based on a median split of their DSS-4 ratings, BPD patients were assigned to two subgroups with high (n=11) versus low (n=11) dissociation. In a second study, we applied a script-driven imagery approach. Before performing the emotional working memory task, BPD patients were exposed to either a personalized script inducing dissociation (n=15) or to a neutral script (n=15). *Results*: In study 1, BPD patients with high dissociation (n=11) showed significantly lower activation in the amygdala and insula after emotional distraction compared to BPD patients with low dissociation (n=11). In study 2, similar patterns of brain activation in the amygdala and insula were observed in BPD patients, who had been exposed to the dissociation script compared to BPD patients in the neutral condition. *Conclusion*: Findings of our studies suggest that dissociative states are associated with lower activation in limbic brain regions during emotional challenge in interpersonally traumatized individuals with BPD.

### Blunted and discordant affect: syndrome specific to complex trauma 10:15–10:30

#### W. D'Andrea Department of Psychology, University of Michigan, Ann Arbor, MI, USA

The traditional conceptualization of traumatized patients relies heavily on expectations of physiological hyperarousal as a component of symptom presentation, with the assumption that trauma exposure primarily manifests as PTSD. Numerous psychobiological studies have demonstrated that patients with PTSD show strong autonomic and neurological hyperarousal in response to aversive affective stimuli. However, psychobiological data in this presentation suggest three conclusions which counter the prevailing assumption of hyperarousal: (1) that complexly traumatized patients will show evidence of hypoarousal as well as of hyperarousal; (2) that hypoarousal has significant consequences for affect, cognition, and relationship and; (3) that the manifestation of hypoarousal occurs transdiagnostically, in mood, anxiety, and personality disorders. This presentation presents data supportive of the construct of blunted and discordant affect (BADA) accumulated from three separate studies. In the first study, we present data from a trauma-exposed sample stratified on exposure severity; startle probes are presented while autonomic and reflexive responses are recorded. The subgroup with acute, late-onset exposure showed the expected hyperaroused profile, while the subgroup with chronic, early-onset trauma exposure exhibited a profile of blunted autonomic response featuring both sympathetic and parasympathetic withdrawal, followed by late exaggerated physiological rebound. Next, we present data from a sample of women with borderline personality disorder. Here, we find that increased trauma severity predicts blunted autonomic activity and decreased cognitive processing. Finally, we demonstrate the existence of a subgroup of depressed and anxious patients who show amygdala hypoarousal, rather than the expected hyperarousal; furthermore, complex trauma exposure is disproportionately represented in the hypoaroused group, and hypoarousal predicts treatment resistance. Taken together, these findings support the existence of a transdiagnostic construct of BADA strongly associated with early complex trauma history.

### Consciousness and dissociation: how mind/brain/body can adapt to overwhelming experience 10:30–10:45

#### R. Lanius and P. A. Frewen Western University of Canada, London, ON, Canada

Consciousness refers to the quality or state of an organism's awareness of both its external and internal environment. Confrontation with overwhelming experience from which actual escape is not possible, such as childhood abuse, various forms of torture, as well as war trauma often confronts the mind, brain, and body with the challenge of finding an escape from both the external and internal environment when no actual escape is possible. How can consciousness be altered to make such an escape possible? Alterations in various dimensions of consciousness, including 1) temporality (time sense), 2) narrative (the story-like nature of thought as incorporating content, perspective, and structure), 3) embodiment (sense of having, consciously being in, and belonging to a body), and 4) affect (the experience of emotional feelings) can aid the mind, brain, and body's escape from both the external and internal environment when no actual escape is possible. In this symposium, we will describe in detail how each of the four dimensions of consciousness can emanate from overwhelming experience and their relationship to symptoms of dissociation often observed in chronically traumatized individuals. We will also discuss the method of neurophenomenology that has been used to study different dimensions of consciousness. Finally, implications for treatment will be described.

### Structural alterations associated with dissociative traits in PTSD 10:45–11:00

#### M. Pagani^1^, D. Nardo^2^ and R. Lanius^3^

^1^Institute of Cognitive Sciences and Technologies, CNR, Rome, Italy; ^2^Neuroimaging Laboratory, Santa Lucia Foundation, Rome, Italy; ^3^Department of Psychiatry, Schulich School of Medicine and Dentistry, The University of Western Ontario, London, ON, Canada

The nature of comorbidity between PTSD and dissociation is still largely unknown, and the role that dissociation plays in the genesis of PTSD and its current taxonomy as an anxiety disorder, separate from dissociative disorders, has been put into question. Neuroimaging studies have shown a rather heterogeneous pattern of results, by which dissociation might be associated with functional alterations in various areas. This study used Voxel-Based Morphometry (VBM) to investigate brain structural alterations related to trait dissociation and its relationship with posttraumatic stress disorder (PTSD). Thirty-two subjects either developing (N=15) or non-developing (N=17) PTSD underwent MRI scanning and were assessed with the Dissociative Experience Scale (DES), subscales for pathological (DES-T) and non-pathological trait (DES-A) dissociation, and other clinical measures. Gray matter volume (GMV) was analyzed by using VBM as implemented in SPM. PTSD and non-PTSD subjects were compared to assess brain alterations related to PTSD pathology, whereas correlation analyses between dissociation measures and GMV were performed on the whole sample (N=32), irrespective of PTSD diagnosis, to identify alterations related to trait dissociation. As compared to traumatized controls, PTSD subjects showed reduced GMV in the prefrontal cortex, hippocampus, and lingual gyrus. Correlations with dissociation measures (DES, DES-T, DES-A) consistently showed increased GMV in the medial and lateral prefrontal, orbitofrontal, parahippocampal, temporal polar, and inferior parietal cortices. PTSD and dissociation seem to be associated with opposite volumetric patterns in the prefrontal cortex. Trait dissociation appears to involve increased GMV in prefrontal, paralimbic, and parietal cortices, with negligible differences between pathological and non-pathological dissociation. Hence in subclinically dissociated subjects, the tendency to experience pathological dissociative phenomena and absorption or imaginative involvement widely share the same neural substrates supporting a view of dissociation along a continuum.

## Psychobiology and PTSD
*Symposium: Neurobiological effects of treatment for posttraumatic stress disorder and borderline personality disorder*

### Neurobiological correlates of PTSD and related psychotherapeutic treatment 11:45–12:00

#### M. Pagani Institute of Cognitive Sciences and Technologies, CNR, Rome, Italy

Recent studies have shown that psychological trauma may cause anatomical and functional changes resulting in post-traumatic stress disorder (PTSD). It has become increasingly clear that a number of specific brain structures play a key role in the generation of PTSD symptoms. These structures are involved in emotional, memory, linguistic, visuospatial and motor processing, all of which might be affected in the disorder. Different imaging techniques have been used to measure cerebral hemodynamic changes. Positron Emission Tomography and Single Photon Emission Computed Tomography studies have found regional cerebral blood flow (rCBF) changes during trauma recall in PTSD patients with reports of rCBF being either increased or decreased mainly within hippocampus, amygdala, medial pre-frontal cortex, including orbito-frontal and anterior cingulate cortices, as well as other cortical and subcortical structures. Furthermore, structural alterations as investigated by Magnetic Resonance Imaging have been shown to occur either as a predisposing factor for the development of PTSD, or as a neurotoxic consequence. On the other hand, specific neural structures have been recognized to play a role in the generation of PTSD symptoms consistently indicating amygdala hyperreactivity, and a correspondingly reduced medial prefrontal cortex (including anterior cingulated cortex) control over amygdala, as the core functional neural mechanisms implicated in PTSD. Over the past decade neuroimaging techniques have also been used to shed light on the neurobiological correlates of the various psychotherapies treating PTSD clinical symptoms in the attempt to reveal their neurobiological effects. The most recent findings about the neurobiological correlates of PTSD and the related psychotherapies will be reviewed. The different studies will be critically discussed, as well as the functional model underlying the pathophysiological mechanisms of PTSD. The results of an electroencephalographic (EEG) study monitoring for the first time the neuronal activation changes occurring during EMDR therapy in PTSD will be also presented.

### Treatment effects on insular and anterior cingulate cortex activation during classic and emotional Stroop interference in child abuse related complex PTSD 12:00–12:15

#### K. Thomaes^1^, E. Dorrepaal^1^, N. Draijer^1^, M. De Ruiter^2^, B. M. Elzinga^3^, A. Van Balkom^1^, J. Smit^1^ and D. Veltman^1^

^1^GGZ InGeest/Department of Psychiatry, VU University medical center, Amsterdam, The Netherlands; ^2^AMC Academic Psychiatric Center, AIAR, Amsterdam, The Netherlands; ^3^Department of Clinical and Health Psychology, Leiden University, Leiden, The Netherlands

*Background:* Functional neuroimaging studies have shown increased Stroop interference coupled with altered anterior cingulate cortex (ACC) and insula activation in posttraumatic stress disorder (PTSD). These brain areas are associated with error detection and emotional arousal. There is some evidence that treatment can normalize these activation patterns. *Method*: At baseline, we compared classic and emotional Stroop performance and BOLD responses (functional MRI) of 29 child abuse related complex PTSD patients with 22 non-trauma exposed healthy controls. In 16 of these patients, we studied treatment effects of psycho-educational and cognitive behavioral stabilizing group treatment (EXP) added to treatment as usual (TAU) versus TAU only, and correlations with clinical improvement. *Results:* At baseline, Complex PTSD patients showed a trend for increased left anterior insula and dorsal ACC activation in the classic Stroop. Only EXP patients showed decreased dorsal ACC and left anterior insula activation after treatment. In the emotional Stroop contrasts, clinical improvement was associated with decreased dorsal ACC activation and decreased left anterior insula activation. *Conclusions:* We found further evidence that successful treatment in child abuse related complex PTSD is associated with functional changes in ACC and insula, which may be due to improved selective attention and lower emotional arousal, indicating greater cognitive control over PTSD symptoms.

### Effects of dialectical behavior therapy on pain-mediated affect regulation in borderline personality disorder 12:15–12:30

#### I. Niedtfeld^1^, D. Winter^1^, R. Schmitt^2^, M. Bohus^1^, S. Herpertz^2^ and C. Schmahl^1^

^1^Department of Psychosomatic and Psychotherapeutic Medicine, Central Institute of Mental Health, Medical Faculty Mannheim/Heidelberg University, Heidelberg, Germany; ^2^Department of General Psychiatry, Medical Faculty Heidelberg/Heidelberg University, Heidelberg, Germany

*Background:* Disturbed affective responding and affective dysregulation are core symptoms of Borderline Personality Disorder (BPD). At a neurobiological level, findings point to a conjunction of limbic hyperarousal and dysfunctional prefrontal regulation mechanisms. A second core symptom in BPD is self-injurious behavior (SIB), which is known to correspond to affective dysregulation and is used by patients to escape from aversive tension or undesired emotions. Earlier findings point to an improved inhibition of limbic arousal by means of painful stimulation in BPD. *Methods:* We investigated the effects of dialectical behavior therapy (DBT) on the role of pain in emotion regulation in BPD. We conducted an fMRI study with 14 patients and 16 healthy subjects using picture stimuli to induce negative (vs. neutral) affect and thermal stimuli to induce heat pain (vs. warmth perception) before and after 12 weeks inpatient DBT treatment. *Results:* Before therapy, painful stimuli led to stronger activation in the middle frontal gyrus in BPD patients compared to healthy controls. Negative pictures combined with painful stimuli led to more activation of rostral anterior cingulate cortex in patients with BPD compared to negative pictures combined with baseline temperature, which normalized after DBT. Furthermore, patients showed more activation in the middle frontal gyrus in response to negative pictures, even when they were not combined with painful stimuli. *Conclusions:* The results are in line with previous findings on the soothing effect of self-injury. Furthermore, we found that DBT treatment led to diminished activation of limbic pain-related regions and enhanced prefrontal emotion regulation processes.

### The acute effects of oxytocin administration on emotional processing in patients with posttraumatic stress disorder 12:30–12:45

#### L. Nawijn^1^, S. Koch^1^, M. Van Zuiden^1^, J. Frijling^1^, D. Veltman^2^ and M. Olff^1^

^1^Department of Psychiatry, Academic Medical Center, University of Amsterdam, Amsterdam, The Netherlands; ^2^Department of Psychiatry, VU University Medical Center, Amsterdam, The Netherlands

Posttraumatic stress disorder (PTSD) is associated with altered emotional processing and deficient emotion regulation abilities. The most prominent emotional alteration in PTSD is an exaggerated fear response, which is neurobiologically associated with amygdala hyperresponsivity. Indeed, recent meta-analyses have shown that amygdala hyperresponsivity is a consistent finding in PTSD. In healthy participants, the neuropeptide oxytocin has been shown to dampen amygdala reactivity to emotional faces (Kirsch et al., 2005). Moreover, in patients with generalized anxiety disorder, oxytocin was found to dampen amygdala responsivity to emotional faces to levels similar to that of healthy control participants (Labuschagne et al., 2010). Therefore, we investigated whether intranasal oxytocin administration in PTSD patients dampens amygdala reactivity during emotional face processing. In a randomized double-blinded placebo-controlled functional MRI (fMRI) study, we investigated the effects of intranasal oxytocin administration on emotional neural processing using a within-subjects design. In a randomized order, each participant received one dose of intranasal oxytocin and one dose of placebo. Participants performed an emotional face matching task, in which emotional faces (angry/fearful faces and happy/neutral faces) and visuomotor control blocks (scrambled faces) were shown. Our first results indicate that intranasal oxytocin reduces amygdala reactivity towards emotional faces in PTSD patients. These results are the first fMRI data suggesting that intranasal oxytocin administration could reduce the amygdala hyperreactivity consistently observed in PTSD. These initial findings suggest that oxytocin may be a promising agent in medication-enhanced psychotherapy in PTSD.

## Afternoon
*Invited Symposium: Genetic and epigenetic risk factors for PTSD*

### Traumatic stress and memory-related genes in the development of Post-traumatic Stress Disorder 15:30–15:45

#### Iris-Tatjana Kolassa^1^, S. Wilker^2^, V. Ertl^2^, B. Lingenfelder^2^, S. Kolassa^2^, A. Papassotiropoulos^2^, D. de Quervain^2^ and T. Elbert^2^

^1^Department of Psychology, University of Konstanz, Germany; ^2^Clinical & Biological Psychology, Institute of Psychology and Education, University of Ulm, Germany

Genetic risk factors and environmental exposure (i.e., traumatic load) interact to influence the individual vulnerability to develop post-traumatic stress disorder (PTSD). Several genetic risk factors have been identified that influence the formation of a strong associative fear memory in PTSD, which stores sensory-perceptual representations of traumatic memories and leads to intrusive re-experiencing. For example, genetic variations associated with increased fear conditioning, reduced fear extinction, increased emotional memory or long-term memory formation have also been associated with an increased risk for PTSD. The talk will give an overview on the genetics of PTSD, concluding that genetic factors enhancing (emotional) memory formation, which should be evolutionary adaptive, may have a dark side, namely an increased risk for PTSD in case of traumatic life events.

### DNA methylation at the glucocorticoid receptor gene promoter is linked to PTSD risk in genocide survivors 15:45–16:00

#### V. Vukojevic, I. Kolassa, A. Heck, M. Fastenrath, L. Gschwind, C. Vogler, P. Demougin, F. Peter, A. Stetak, T. Elbert, A. Papassotiropoulos and Dominique J.-F. De Quervain Department of Psychology, Division of Molecular Neuroscience, University of Basel, Basel, Switzerland

The HPA-axis plays an important role in the regulation of memory. PTSD, which is characterized by intrusive traumatic memories, is accompanied by a dysregulation of the HPA-axis. This dysregulation is thought to represent-at least in part-a pre-trauma risk factor for the disorder. In the present study, we investigated if epigenetic variability of the human glucocorticoid receptor gene (*NR3C1*) promoter in survivors of the Rwandan genocide is related to PTSD. We found a significant negative correlation of DNA methylation levels at the *NR3C1* promoter with symptoms (intrusions and avoidance, but not hyperarousal) and the risk of PTSD. The significant correlation was restricted to the NGFI-A (nerve growth factor induced A) transcriptional factor-binding site of the *NR3C1* promoter. Inter-individual differences in methylation levels were not related to the number of traumatic live events, suggesting that the differences in methylation levels pre-existed the traumatic events. In a further experiment, we found that DNA methylation levels correlated negatively with *NR3C1* expression. Finally, an fMRI study in healthy humans revealed that inter-individual differences in DNA methylation at the *NR3C1* promoter were related to activation differences in the medial temporal lobe during memory recognition. Together, these findings suggest that an epigenetic modification of the glucocorticoid receptor gene promoter may act as a pre-trauma risk factor for PTSD, possibly through a modulation of memory processes.

### The transgenerational scars of violence 16:00--16:15

#### H. Gunter, K. Radtke, M. Ruf, K. Dohrmann, M. Schauer, A. Meyer and T. Elbert Department of Biology, University of Konstanz, Germany

Stress in the familial environment can have a profound influence on the physical and psychological health of parents and children alike. As familial abuse results in higher rates of depression and suicide, research aimed at monitoring and mitigating the negative effects of family stress can have a powerful societal impact. Recent research on both humans and rodent models demonstrates that epigenetic marks provide a powerful mechanistic link between stressors and behavioural outcomes, providing avenues for objectively assessing the impact of increased stress. Our research examines the epigenetic and psychosocial impact of several stressors experienced in German households, on women and their offspring. Previously we have shown that gestational exposure to intimate partner violence results in elevated levels of methylation in the promoter of glucocorticoid receptor (GR), a key regulator of HPA-axis. We will present the results of our current interdisciplinary investigation of 50 mother-child dyads, aimed at determining the extent to which gestational and familial environment can alter the long-term epigenetic imprinting of children and their behaviour. Our study combines numerous structured interviews that assess allostatic load in mothers (for example the Combined Abuse Scale and Everyday Stressors Index) and behavioural investigations of their offspring (for example, the Strength and Difficulties Questionnaire), with genome-wide investigations of DNA methylation. This allows us to draw a molecular link between the experience of prenatal and maternal stress and potential psychopathology later in life, opening the door for early objective screening and the treatment of 'at risk' individuals.

## Psychobiology and PTSD
*Invited Symposium: Treatment of sleep disturbances in PTSD*

### Medication for sleep disorders in Post-traumatic stress disorder with focus on alpha-blockers 16:45--17:05

#### J. De Jong^1^, M. Van Der Gaag^2,3^ and W. Van Der Does^4^

^1^Parnassia Group, PsyQ Psychotrauma, The Hague, The Netherlands; ^2^Parnassia Group, Parnassia, The Hague, The Netherlands; ^3^VU ParnassiaBavoGroup, VU University, Amsterdam, The Netherlands; ^4^Faculty of Psychology, Leiden University, Leiden, The Netherlands

*Background*: Sleep disorders are very common in patients with post-traumatic stress disorder (PTSD). Some advances in pharmacological treatment have occurred over the last years. *Objective*: An overview of the literature of the last years about the medication for sleep disorders in PTSD, and the influence of medication used for PTSD on sleep disorders with special focus on alpha-blockers and preliminary results of a new study with doxazosin. *Method*: Several databases have been searched and relevant searches done. Based on articles found, some other articles have been chosen and used for this presentation. *Results*: Literature shows that disturbed sleep can contribute to maladaptive trauma responses. Sleep disorders affect treatment negatively, and treatments focused on sleep can influence treatment for PTSD in a positive way. Prazosin is a alpha-blocker and has shown to reduce nightmares in frequency and intensity; there are some hypotheses about working-model. Another alpha-blocker, doxazosin has shown some positive results in a small pilot study. Together with this study, preliminary results of a new study will be presented. *Conclusion*: Sleep is one of the most important factors in development and treatment of PTSD, and medication can influence the course of the sleep disorders in PTSD as well as PTSD. There are several reasons why alpha-blockers can have a positive effect on sleep disorders in PTSD. Prazosin has shown good results, and doxazosin is promising.

### Prazosin versus placebo in the treatment of sleep disturbances in veterans with PTSD: a placebo controlled randomized clinical study 17:05--17:25

#### S. Van Liempt^1^, J. Arends^2^, J. Smulders^1^, R. Kahn^3^ and E. Vermetten^1^

^1^Military Mental Health-Research Center, Utrecht, The Netherlands; ^2^Kempenhaeghe Centre for Sleep Wake Studies, Kempenhaeghe, Heeze, The Netherlands; ^3^Department of Psychiatry, Rudolf Magnus Institute of Neuroscience, Utrecht, The Netherlands

*Background*: Sleep complaints are frequently reported in patients with PTSD and affect about 70% of patients, with frequent nightmares and anxiety dreams, frequent awakenings, difficulty falling asleep, decreased total sleep time, and restless sleep as most reported complaints. Promising studies have been published in the treatment of PTSD related sleep complaints for prazosin, a alpha-1 adrenoceptor antagonist. No studies have been reported that additionally assessed sleep architecture. We performed a randomized placebo-controlled trial in veterans with PTSD to study the effect of prazosin on the subjective sleep quality as well as sleep architecture. *Method*: Veterans with PTSD were treated for 8 weeks with either active medication or placebo in climbing dosages up to 14 mg. Before the treatment started, and in the last week of treatment, two nights of polysomnographic registrations were conducted in the homes of the patients. *Results*: In the prazosin group CAPS score was significantly lower after treatment, in the absence of a group×time effect compared to the placebo group. There was a trend for a lower PTSD severity score at end of the study phase. Sleep related problems were lower in both prazosin and placebo group, but did not differ between groups. Polysomnographic recordings showed a trend for an increase in TST in the prazosin group. This increase was predominantly due to an increase of REM sleep in the prazosin group. *Discussion*: These results showed a trend for increased total sleep time, which may be predominantly due to an increase in REM sleep, and also a trend for a decline in PTSD severity when compared with placebo. Furthermore, an improvement on subjective sleep parameters emerged. A limitation of this study was the small sample size, which limited the power to detect large differences.

### Imagery rehearsal for post-traumatic nightmares: observations from two clinical trials 17:25--17:45

#### J. Cook^1,2^, G. Harb^3,4^ and R. Ross^3,4^

^1^Department of Psychiatry, Yale School of Medicine, New Haven, CT, USA; ^2^National Center for PTSD, West Haven, CT, USA; ^3^University of Pennsylvania, Philadelphia, PA, USA; ^4^Philadelphia VA Medical Center, Philadelphia, PA, USA

Imagery Rehearsal (IR), a cognitive-behavioral therapy for the treatment of post-traumatic nightmares, involves selecting a target nightmare, changing the storyline, and rehearsing the new dream image. This presentation will review findings on dropout and outcome from a randomized controlled trial (RCT) of IR in 124 US Vietnam veterans with PTSD and recurrent nightmares. Intent-to-treat analyses indicated that veterans who received six sessions of manualized IR delivered in group format did not significantly improve more than veterans in the psychotherapy comparison condition in regards to their nightmare frequency or sleep quality. In fact, IR delivered in group did not produce substantive improvement in these older US veterans with chronic, severe PTSD. In addition, dropout was higher in IR than in the comparison condition. IR was most effective when the rescripted dream incorporated a resolution of the nightmare theme and excluded violent details. This presentation will also highlight observations from an ongoing RCT of IR delivered on an individual basis to US veterans from the wars in Iraq and Afghanistan. Although recruitment has been slow for this trial, initial impressions of efficacy are promising.

## ORAL, JUNE 7 HALL GARGANELLI

## Morning
*Open Papers: Children and young people I*

### Expressive arts therapy (EXIT) for unaccompanied minor refugee boys (15–18) in transit refugee centers 10:00–10:15

#### M. A. Meyer Demott Norwegian Centre for Violence and Traumatic Stress Studies, Oslo, Norway

*Objectives:* The lecture will present the research project EXIT. *Content:* EXIT (expressive arts in transition) developed for stabilizing people who live under extreme stress and/or have survived human- or nature-induced trauma. EXIT focuses on enhancing movement, imagination, engagement, connection, here and now, safety, and responsibility. The lecture will be about the research project where EXIT is being carried out with 200 refugee boys (15–18) in a randomized controlled study in Norway. This workshop/lecture will be of relevance and interest to those working with families, multicultural groups, adolescents, and trauma survivors.

### Emotional availability among traumatized refugee families 10:15–10:30

#### M. Brendler Lindqvist^1^, A. Daud^2^ and J. Hermansson Tham^1^

^1^Red Cross Centre, Stockholm, Sweden; ^2^Karolinska Institutet Stockholm, Stockholm, Sweden

The early relationship of parent-infant is fundamental for the survival of the infant and basic for development of a child's social, emotional, and cognitive health. According to research, refugee parents with complex PTSD risk to transform their symptoms to their children. Red Cross Centre for Tortured Refugees, RKC in Stockholm, Sweden started in 2011 a clinical pilot project with the aim to explore and evaluate a treatment program based on the attachment theory and trauma theory for parents and their infants aged 6-24 months, as a possible way to prevent second-generation traumatization. The project runs in co-operation with the Karolinska Institutet Stockholm, Department of Women's and Children's Health. At the conference, we would like to present the work done during 2011–2013 at RKC. During two years, a treatment program for infants and small children to traumatized refugee parents with complex PTSD has been tested. The program, consisting of psycho pedagogical family interventions, video taping, and interplay therapy in groups, has been used. Nine families have taken part in two separate treatment periods. In the first period, there were five families and in the second period four families. All families were Arabic speaking. An Arabic interpreter has been used in all parts of the program. The project has been evaluated by emotional availability scales, EAS, and interviews. The preliminary results of the project indicate a need for developing a treatment program focusing on emotional availability between mother and child in refugee families as a preventive intervention. The results also raise questions about design and the treatment program.

### Disclosing complex trauma in childhood: a train-the-trainer's model for competence building in local services 10:30–10:45

#### I. A. Nordhaug^1^, D. Nordanger^2^, R. Dybsland^1^, E. Rutle Johansson^1^ and V. A. Johansen^1^

^1^Resource Centre on Violence, Traumatic Stress and Suicide Prevention-Region West (RVTS West), Bergen, Norway; ^2^UNI Health, RKBU West, RVTS West, Bergen, Norway

To facilitate disclosure of complex trauma in childhood, Resource Centre on Violence, Traumatic Stress and Suicide Prevention-Western Norway (RVTS West) has established *Consultation Teams* (CTs) in more than 30 local authorities of Western Norway. The CT's function is to support local professionals who have concerns regarding possible child abuse. Professional can present worries anonymously to the teams and get advice on how to proceed. To enable CT members for the task, RVTS West has developed a standardized competence building program composed of four two-day modules. The first three modules cover knowledge about child abuse, consultation method, and disclosure conversations with children. The fourth module trains CT members to become trainers themselves: RVTS West has developed a *teaching package* for this purpose, including a PowerPoint presentation, pedagogic videos (e.g., on how to do disclosure conversations), and a detailed guide/handbook on how to present the material. In this way, competence on this area can be spread in the region through the CTs. The material developed also serves as a *manual for implementation* of the model in other regions or countries. The model and the teaching package will be presented at the conference.

### Attachment and PTSD in multiple trauma samples 10:45–11:00

#### A. Elklit, T. Andersen and K. Karstoft National Center for Psychotraumatology, University of Southern Denmark, Denmark

Attachment orientation has been found to be associated with severity of posttraumatic stress disorder (PTSD) after the exposure to a potentially traumatic event. However, the exact relationships between trauma exposure, attachment orientation, and PTSD remain unknown. In this study, we investigated the relationship between trauma type, attachment, and PTSD in a large multiple trauma sample (*n*=5042). All participants were assessed for PTSD symptomatology using the Harvard trauma questionnaire (HTQ) and for attachment orientation utilizing the revised adult attachment scale (RAAS). In line with our hypotheses, we found that a secure attachment style is related to lower PTSD severity, while the insecure attachment styles are related to higher PTSD severity. Furthermore, we found that anxious as well as avoidant attachment is related to high PTSD severity, albeit the association is stronger for anxious attachment. Furthermore, we found that these associations between attachment and PTSD severity are valid across trauma types. The results underscore the importance of attachment orientations in understanding adaptations to traumatic experiences. Moreover, trauma-focused interventions can be improved by taking attachment styles into consideration in treatment planning. In particular, individuals with negative models of self (preoccupied and fearful) may need additional support mobilizing an internal sense of security.

### Forensic psychological expertise as part of the process of prevention or reprocessing of PTSD 11:00–11:15

#### G. A. Saba Associazione Artemisia, Florence, Italy

Children can be involved in legal proceedings in many ways: as children of separating parents who don't protect them from their own conflicts, or as victims of domestic violence or sexual abuse or as witnesses of crime. In these cases, children can be heard from social workers or by the court and may be subject to psychological evaluation, for example, in order to assess their skills of witnesses or the presence and extent of any post-traumatic experiences, or can be evaluated together parents and their parenting skills to identify the best child custody and the best relationship with non-residential parent. Professionals, especially psychologists or child psychiatrists, may be called to provide assistance to children during hearing, especially after the Italian ratification of the Lanzarote Convention and resulting changes in legal codes, and their work can be a real help for children or may reiterate the negative effects of traumatic experiences. In case of psychological evaluation of children involved in legal proceedings, the ways in which the expertise is carried out have high potentiality to initiate a process of restructuring and recovery personal skills, to improve self-confidence, to increase self-esteem, the perception of better control of their life and greater sense of personal power; an actions against the effects of PTSD. Instead, an expert misconduct may confirm the negative expectations of children, in order to trust in themselves and in others, it may increase the difficulties, the sense of confusion and uncontrollability of the events that affect them and can be a new and stronger traumatization. Also because children have great expectations in justice and to see recognition of their rights and Also because children have great expectations with respect to justice and they want the recognition of their rights and wrongs suffered.

## Open Papers: Children and young people II

### Psychopathology among victims of early stress exposure 11:45–12:00

#### A. Maia and R. Pinto University of Minho, School of Psychology, Braga, Portugal

The “complex trauma” is the term used to describe the exposure at multiple and/or chronic and prolonged adverse experiences (Cook et al., 2005). These chronic exposures often occur in home, result of physical, emotional, sexual abuse, and neglect. In consequence, the child has high probability to develop trauma and vulnerability to cope with later traumatic incidents (Gunnar & Quevedo, 2007). Although some limitations of the child protection services (CPS), the families and children who were the subject of intervention from the authorities are serious cases of abuse and neglect (Pinto & Maia, 2012). This study examined the predictors of psychopathology using two sources of data (official records and self-reports) of ten childhood adversities. The sample included 136 youths, ages 14 to 23 years, identified by CPS prior to age 13 and who lived with their family for at least five years. Results: Global psychopathology was only associated with the total amount of self-reported adverse experiences, but the subscale of depressive symptoms was predicted by both official and self-reported sexual abuse. Females were exposed to more chronic and prolonged adverse experiences than males, based on documented and reported data, and increased risk for psychopathology. Conclusions: Practitioners need to improve the maltreatment identification methods of multiple adverse experiences, particularly the sexual abuse. Maltreated girls should receive special attention, especially those who were sexually abused.

### The unaccompanied foreign minors between trauma and repair interventions. A study on posttraumatic stress disorder, anxiety, depression, and dissociative tendencies in young migrants living in community care for children 12:00–12:15

#### A. Taurino^1^, L. V. Vergatti^1^ and M. T. Colavitto^2^

^1^Department of Educational Sciences, Psychology and Communication, University of Bari, Bari, Italy; ^2^Psychologist, Bari, Italy

*Objective:* The aim of this work is to explore the presence of PTSD symptoms or symptoms associated with this disorder in a small group of unaccompanied foreign children who lived in emergency reception centers and residential communities for minors seeking asylum in Bari land. At the same time, it focused attention about the therapeutic value of community settings in relation to the symptomatic configurations referred above. Specifically, the study first intended to detect the presence of symptoms related to PTSD, anxiety, depression, and dissociative tendencies in the sample examined. The aim is to verify the relationship between the status of unaccompanied foreign minor and the development of PTSD and/or symptoms associated with it. A second aim is to verify any statistically significant differences about the presence of symptoms related to PTSD or associated with it according to the time spent by children in the community. *Statistical methodology and results:* The suggested tests showed the presence of a widespread symptomatology characterizing PTSD, depressive configuration, and mild dissociative tendencies. The Chi-square analysis confirmed how the PTSD symptoms, as well as the presence of depressive disorders, were distributed in a statistically significant manner in relation to the time spent by the children in the community. Both the univariate analysis of variance and the Kruskal-Wallis analysis performed on the scores related to dissociative tendencies indicated a significant tendency η2 compared to the change in anxiety scores in the times examined. *Conclusions:* This study has highlighted the clinical relevance of interventions performed within residential contexts that were organized on the model of the global therapeutic environment.

### The MATER study: analysis of psychosocial risk factors in perinatal and postnatal maternal complication. Preliminary results 12:15–12:30

#### C. Maiorani^1^, M. Di Mario^1^ and C. Zaiontz^2^

^1^Obstetrics Department of “Maggiore” Hospital, Lodi, Italy; ^2^Post Graduate School of Specialization in Transcultural Psychotherapy, Istituto Transculturale per la Salute, Fondazione Cecchini Pace Milan, IES c/o Universita' Cattolica del Sacro Cuore, Milan, Italy

The purpose of this study, carried out at the obstetrics department of Maggiore hospital in Lodi, is to investigate the influence of personality types in pregnant women on child delivery complications, event appraisal, and adjustment process in the post-partum period. Statistical analysis performed involved Chi-square, *T*-test, and ANOVA. Special attention is given to culture-sensitive guidelines in the structuring of the protocol and by creating an empowerment-based clinical setting through a psychoeducational approach. The sample consists of 500 pregnant women, mostly Italian, participants to a preparatory course to child delivery. The protocol consists of an ample structured questionnaire called MATER (maternal adjustment, transcultural empowerment, representation) based primarily on standardized questionnaires (SCID-II, PDPI, PPQ-modified) administered starting from the third trimester of pregnancy. The protocol is divided into four sections: the first section investigates the self-perception of health in woman before pregnancy according to the principles of the bio-psycho-social-cultural model in order to identify personality-bound risk factors using the criteria of the SCID-II questionnaire. The second section investigates the possible presence of risk factors during pregnancy. The third part, focused on delivery, explores the presence of peri and postnatal complications involving clinical data, and a short questionnaire administered two days after child delivery. The fourth section entails a questionnaire aimed at identifying possible medical complications in the post-partum period and the presence of post-partum posttraumatic stress disorder (PP-PTSD) through the administration of PPQ-modified functioning as a preliminary assessment for post-partum PTSD. According to assessments, the clinical range for high-risk mothers is set at 19 or higher (Callahan, Borja, Hynam, 2006). New mothers who have a high-risk range according to PPQ-modified will then be engaged for clinical interviews, psychological diagnosis, and treatment when appropriate.

### The impact of earthquake exposure on psychological well-being of adolescents in Sichuan Earthquake: role of cognitive flexibility 12:30–12:45

#### F. Fu Department of Social Work and Social Administration, Hong Kong University, Hong Kong

Long-term effect of trauma on adolescents has always been concerned in trauma field. This research aimed at examining the impact of earthquake exposure on psychological well-being of adolescents as well as the role of cognitive flexibility between them three years after Sichuan earthquake. A total of 934 adolescents with the mean age was 16.74 years (SD=0.868; range=15–19 years) filled in Earthquake Exposure Inventory (self-developed), CFI (Calhoun & Tedeschi, 1998) and PWB (Ryff & Keyes, 1995) after informed consent. First, ANOVA analysis results showed that there were significant differences of psychological well-being in the dimension of relocation of school (*T*=3.09, *p*<0.01), damage of property (*F*=2.73, *p*<0.05), and damage of school (*F*=5.76, *p*<0.01), and adolescents who had been relocated and suffered the damage of property and school had significant higher psychological well-being than those without such experiences. Furthermore, as for the role of cognitive flexibility, linear regression was performed and cognitive flexibility was found to have moderate relationship between earthquake exposure and psychological well-being, which implied that as the increase of cognitive flexibility, the positive impact of earthquake exposure on psychological well-being also increased. The results of this study provide the evidence for possible long-term positive effect of trauma on adolescents and highlight the importance of enhancing the cognitive flexibility in the recovery from trauma.

References

Calhoun, L. G., & Tedeschi, R. G. (Eds.). (1998). *Posttraumatic growth: Future directions*. Mahwah: Lawrence Erlbaum Associates.

Ryff, C. D., & Keyes, C. L. (1995). The structure of psychological well-being revisited. *Journal of Personality and Social Psychology, 69*, 719–727.

### Shame, self-blame, and gender differences in young survivors of a terrorist attack: the Utøya study 12:45–13:00

#### H. Flood Aakvaag^1^, S. Thoresen^1^, T. Wentzel-Larsen^1^ and G. Dyb^2^

^1^Norwegian Centre for Violence and Traumatic Stress Studies, Kirkeveien, Oslo, Norway; ^2^Institute of Clinical Medicine, University of Oslo, Oslo, Norway

*Background:* It is well documented that women have a higher conditional risk of PTSD compared to men. The reason for this is unresolved and has been much discussed in the literature. Several studies also document an association between shame, self-blame, and mental health. This study investigates if shame and self-blame may contribute to women's increased risk of posttraumatic stress reactions (PTSR) in a sample of young survivors of a terrorist attack. *Method:* Totally, 325 survivors (response rate=66%) from July 22nd, 2011 terror attack on Utøya Island in Norway were interviewed face-to-face 4-5 months after the event. Trauma exposure was measured by a series of dichotomous questions relating to life threat, witnessing, sensory impressions, loss of someone close, and physical injuries. Shame and self-blame were measured by one item each, and PTSR were measured by the UCLA PTSD reaction index. Gender differences in shame and self-blame were investigated using chi square. Linear regression and chi-square tests were employed to investigate associations between gender, shame, self-blame, and PTSR. *Results:* No significant gender differences were found in level of trauma exposure. Girls reported significantly more shame, but not more self-blame, than boys. Shame and self-blame were associated with PTSR after controlling for exposure, gender, and other potential confounders. Girls also had significantly higher levels of PTSR. This was still true after adjusting for shame and self-blame. *Conclusions:* Shame and self-blame may contribute to PTSR in both genders, and may be of concern for preventive and treatment work in the aftermath of trauma. Shame and self-blame did not contribute much to explain the gender difference in PTSR. However, the higher level of shame in girls indicates that addressing shame may be especially important when working with women.

## Afternoon
*Open Papers: Children and young people III*

### Posttraumatic responses to the 22nd of July 2011 Oslo terror attacks among Norwegian high school students 15:15–15:30

#### D. Nordanger^1^, M. Hysing^2^, M. Posserud^3^, A. Johansen Lundervold^2^, R. Jacobsen^4^, M. Olff^5^ and K. M. Stormark^4^

^1^Uni Health, Regional Centre for Child and Youth Mental Health and Child Welfare (RKBU West); Haukeland University Hospital, Resource Centre on Violence, Traumatic Stress and Suicide Prevention-West; ^2^Department of Biological and Medical Psychology, University of Bergen, Bergen, Norway; RKBU West; ^3^Department of Child and Adolescent Psychiatry, Haukeland University Hospital, Bergen, Norway; ^4^Faculty of Health Sciences, University of Tromsø, Tromsø, Norway; ^5^Academic Medical Center, Department of Psychiatry, University of Amsterdam, Amsterdam, The Netherlands

The July 22, 2011 Oslo terror was defined as a national disaster. Former studies on terror attacks and mass shootings have shown elevated levels of posttraumatic complaints both in direct victims and in general populations. Little is known about how such extreme events in a generally safe society such as Norway would affect an adolescent population. This study examines posttraumatic stress reactions and changes in worldview in relation to risk factors among 10,220 high school students using data from the ung@hordaland survey. One out of five of respondents knew someone directly exposed, 55.7% felt the events to some extent as threatening to their own or close ones' lives and 79.9% reported their worldview to be changed. Concerning PTSD symptoms, 0.8% reported substantial distress on the intrusion area, 4.9% on the avoidance area, and 1.1% on the hypervigilance area. Greater personal proximity to the events, higher levels of perceived life threat, and being a female or an immigrant predicted higher levels of PTSD symptom distress. Results indicate that the terror events made a deep impression on Norwegian adolescents, but without causing markedly elevated levels of PTSD symptomatology in the general young population.

### Narration in child trauma therapy: helpful or harmful? 15:45–16:00

#### J. Cohen and A. Mannarino Allegheny General Hospital; Pittsburgh, PA, USA

Many evidence-based child trauma therapies include a trauma narrative component but therapists, children, and parents often wonder whether talking about the child's trauma experiences is helpful, or in some cases whether this may even be detrimental. This presentation describes a recent empirical study that evaluated the use of trauma narration in one evidence-based treatment for young children after sexual abuse. Two hundred children aged 4-11 years were randomized to receive Trauma-Focused CBT (TF-CBT) with or without the trauma narrative and processing component, provided over 8 or 16 sessions. Results showed that regardless of length of treatment or inclusion of trauma narrative, TF-CBT was effective in significantly improving children's symptoms, parenting skills, and children's safety skills. However, some significant differences were found based on group assignment. TF-CBT provided in 8 sessions with the trauma narrative was the most effective and efficient condition for improving children's abuse-related fear and general anxiety, as well as parental abuse-related distress. On the other hand, parents assigned to the 16 session, no narrative condition reported greater increases in effective parenting practices and fewer externalizing child symptoms at post treatment. The results are discussed in the context of how TF-CBT is applied for children with complex trauma and the need for therapists to tailor evidence-based treatments for individual children's needs.

### Hand injury: evolution of post traumatic stress disorders in adolescents 16:00–16:15

#### O. Convertino^1^, M. Lanzetta^2^, G. Urso^3^, E. Berardi^1^, D. Sala^1^, F. Pirovano^1^, F. Porco^1^, S. Arrigoni^1^, C. Recanati^1^ and V. Matacchiera^1^

^1^Studio Convertino, Monza, Italy; ^2^Italian Institute of Hand Surgery, Monza, Italy; ^3^Rehabilitation Centre “Il Carrobiolo”, Monza, Italy

The paper focuses on a new psychotherapeutic approach taking into consideration two cases with Post Traumatic Stress Disorder (PTSD) diagnosis following accidents involving the hand and the upper limb. The clinical cases have pain and motor deficit of the upper limb. It follows that the trauma involved a psychic transformation symbolically significant. This paper focuses on the consideration of PTSD diagnosis in adolescence and of the symbolic of the hand meaning in the construction of identity, in the process of separation-individuation typical of this developmental period of life. The method has provided the treatment of cases through Co-therapy in Differentiated Times by Multidisciplinary Setting, which integrate the psychological and psysical representation and the identity of patients. The multidisciplinary team, consisting of physiotherapists, orthopedic surgeons and psychotherapists, focuses on creating interventions characterized by a common process, based on the analysis of transference and countertransference in different settings. The method provides analysis test results on time which, adequately processed, enable the design of a customized intervention programme, using several and integrated techniques (role-playing, collage, personal empowerment, pnl etc.). Test material, collected on levels of symptoms, strategic and symbolic, is examined by co-therapists in order to set up intervention. This step is planned in order to dissolve the trauma and support patients in identity definition. In the above cases, the processes related to identity construction in adolescents are influenced by the trauma that causes a dynamic Identity Suspension, connected to transposition and symbolic transfiguration of the damaged hand. In conclusion, the strength of the program is the ability to use the patients's symbolic processes with the different countertransferal aspects, in order to identify and implement the best approaches in the settings (systemic, analytic, cognitive, and PNL).

### The course of posttraumatic stress in children: examination of recovery trajectories following traumatic events 16:15–16:30

#### R. Le Brocque^1^, J. Hendrikz^1^, J. Kenardy^1^ and N. Kassam-Adams^2^

^1^The School of Medicine, University of Queensland, Brisbane, Australia; ^2^Children's Hospital of Philadelphia, PA, USA

Despite the significant impact of posttraumatic stress disorder (PTSD) on child functioning and long term outcomes, studies in pediatric populations are plagued by the methodological problem of small sample sizes. To overcome this, a consortium of researchers have come together to develop the “PTSD after Acute Child Trauma” (PACT) data archive. The archive brings together data from prospective studies in the US, UK, and Australia that have assessed more than 2,500 children and their parents/caregivers following acute trauma such as unintentional injury, acute medical events, motor vehicle accidents, interpersonal violence, and disasters. Evidence suggests that symptoms of PTSD appear to be highest in the immediate acute period and most studies report a decline in the prevalence of symptoms over time. However a significant minority of children may develop chronic symptoms. There is also some limited evidence of delayed onset psychopathology. Although varying rates of symptoms have been observed following trauma, few studies have explored individual recovery patterns. This paper examines the course of posttraumatic stress symptom in children. The aim of this paper is to (1) empirically differentiate posttraumatic stress symptom trajectories in children following trauma; and (2) identify risk factors relating to these symptom trajectories. Using secondary analysis of the existing data, group-based trajectory analysis was conducted to examine child self- and parent-report symptom patterns following trauma. The relationships between risk factors such as child age, gender, type of trauma, and peri-trauma child behavior and symptom trajectory patterns are also examined. Results are discussed in terms of both clinical and research implications.

## Open Papers: Children and young people IV

### Does a rebel-affiliated collective identity protect former child soldiers from psychopathology? 16:45–17:00

#### V. Ertl^1^, A. Pfeiffer^2^, E. Schauer-Kaiser^2^, T. Elbert^3^ and F. Neuner^1^

^1^Bielefeld University, Bielefeld, Germany; ^2^In Vivo International, Konstanz, Germany; ^3^University of Konstanz, Konstanz, Germany

Child recruitment for war is one of the most considerable violations of child rights. Child soldiers are exposed to warfare, brutal punishment, cruel assaults, mutilations, killings, and sexual attacks. In many cases, they are not only victims of atrocities, but forced perpetrators. These experiences severely impact the youths' mental health, as well as their functionality and reintegration. An under-researched aspect in this context concerns possible influences on the children's identity formation. Aforementioned war experiences and the omnipresent manipulation by rebel-commanders, who prefer children in their armies not only because they manage with little supply, but also because they are considered more malleable, easier to deceive, browbeat, and indoctrinate most probably affect the development of their personal and collective identities. Studies suggest that a strong identification with one's fighting group may be protective concerning psychopathology. Within an extensive epidemiological survey among 12 to 25-year-olds in Northern Uganda (*n*=1113) we found a substantial abduction rate (43%). Among others, we collected data on socio-demography, trauma-exposure, symptoms of PTSD, and depression and perceived stigmatization. We presented 371 former child soldiers with an additional questionnaire investigating on current identification with the rebel-army. We hypothesized identification to act as a moderator in the relationship between trauma-exposure and psychopathology. Multiple regression analyses including rebel-affiliated identity and major predictors of psychopathology showed that identification with the rebel-army did not act protective. On the contrary, concerning symptoms of depression current identification was associated with a higher level of symptoms. However, a significant interaction between trauma-exposure and identification indicated that the strength of this association was smaller for subjects with extreme levels of war-exposure, who presented with high symptoms of depression, regardless of identification. We speculate that a high incongruence between the former child soldiers' current political and social reality and a rebel-affiliated world-view might explain our results.

### Bounce back now: a web-based intervention for youth and families affected by disaster 17:00–17:15

#### K. Ruggiero^1^, M. Price^1^, J. McCauley^1^, K. Gros^2^ and H. Resnick^1^

^1^Department of Psychiatry & Behavioral Sciences, Medical University of South Carolina, Charleston, SC, USA; ^2^Ralph H. Johnson VA Medical Center, Charleston, SC, USA

Disasters confront families with a wide range of stressors, including threat of death or injury, loss of loved ones, limited access to basic necessities, and financial strain. Family roles, routines, and relationships also may be affected. Most youth disaster victims experience only transient distress, but many experience elevated symptoms of PTSD, depression, and substance abuse. Highly accessible, evidence-based interventions are needed. Our research team was awarded a grant from the National Institute of Mental Health to develop and evaluate *Bounce Back Now*, an *e*-health intervention for disaster-affected families. *Bounce Back Now* consists of four adolescent mental health modules and one parent module. Adolescent modules target posttraumatic stress, depression, alcohol use, and smoking. The parenting module includes education on adolescent mental health, parent-child communication, and family routines and relationships. A randomized controlled trial is ongoing. A sample of 2,000 disaster-affected families was recruited from households in the USA that were affected by a major tornado outbreak in the Spring of 2011 that resulted in over 450 deaths. We conducted baseline interviews with 2,000 adolescents and caretakers to assess disaster impact and mental health. Prevalence of post-disaster PTSD and depression among adolescents was 7.3 and 7.9%, respectively. Over 750 caregivers and over 700 adolescents have accessed the study website to date. Families who went to the site were randomized to receive the *Bounce Back Now* intervention *vs* an assessment-only comparison condition. Four month follow-up interviews have been completed with over 1,000 families, and 12-month interviews are ongoing. This presentation will describe data on mental health functioning of these families, and will focus on web-usage statistics and demographic, disaster-related, and mental health-related variables associated with use of the intervention. Adolescent and parent reactions to the site will be described and discussed, as will evaluation and knowledge-change data.

### Acute and posttraumatic stress reactions in young children and their parents following accidents 17:00–17:30

#### M. Gigengack^1^, E. Van Meijel^1^, E. Alisic^2^ and R. Lindauer^1^

^1^Academic Medical Center-De Bascule, Amsterdam, The Netherlands; ^2^Monash Injury Research Institute, Monash University, Melbourne, Australia

Young children can develop posttraumatic stress disorder (PTSD) after an accident, although symptoms may differ from those in older children. We know that in older children, child and parent acute reactions after an accident are associated with the development of child posttraumatic stress (PTS) symptoms. If we had insight into the reactions of *young* children and their parents, we would better be able to diagnose PTSD and offer timely treatment. The purpose of the present study was to explore the acute and PTS reactions of children aged 0-8 years and their parents following an accident. Participants were parents of 104 children, aged 0-8 years, and medically treated in a level I trauma center following an accident during the last 5 years. We conducted semi-structured telephone interviews to explore acute reactions in children and their parents and to screen for PTS symptoms. The descriptions of acute reactions were analyzed qualitatively. If parents reported PTS symptoms, the interview was extended with a semi-structured PTSD interview. We will present the characteristics of children's acute stress reactions mapped on their eventual PTS symptom development. Surprisingly, those children who eventually developed PTS symptoms, were not able to calm down shortly after the accident or were unconscious. Their parents reported high levels of anxiety directly after the accident. A significant minority of the children (13%) showed substantial PTS symptoms, although not all of them met the DSM-IV criteria. Additional symptoms like aggression and new fears were prevalent. Clinical implications for assessment of young children and suggestions for further studies into their acute and posttraumatic stress reactions will be discussed.

### African American children raised by grandparents: the role of trauma and psychological well-being 17:30–17:45

#### S. Kelley and D. Whitley Georgia State University, Atlanta, GA, USA

A growing number of children around the globe are raised by grandparents because their birth parents are unable, or unwilling, to raise them. While the reasons for this type of out-of-home care vary by region, the majority of these children have been traumatized prior to placement with grandparents. Being raised by grandparents can occur abruptly or after a long and difficult period with the birth parents. Despite recent attention to this phenomenon, very little is known about the psychological well-being of these children. The purpose of this study was to explore the trauma histories of African-American children raised by grandmothers and to determine their psychological well-being. The sample was comprised of 1,146 African American children, aged 2–17 years, who were being raised by grandmothers (96%) or great-grandmothers (4%) in parent-absent households. The majority of grandmothers had low educational attainment and resided in an urban area. Almost one-third were 60 years of age or older. Results indicate that the most prevalent traumatic events and situations experienced prior to placement with grandmothers included child abuse and neglect (76.6%), parental substance abuse (67%), abandonment by birth parents (34.5%), parental incarceration (19%), and parental death (17%). Furthermore, almost 60% of children currently had no contact or only sporadic contact with their birth mother. Based on the results of the Child Behavior Checklist, 27.5% of children were determined to be in the clinically elevated range on total behavior problems, with 19.6 and 33.4% scoring in the clinically elevated range for internalizing and externalizing behaviors, respectively. In conclusion, children in out-of-home care with grandmothers typically experience multiple traumatic events prior to their placement. Given their past histories, it is not surprising that one-third of the children had clinically elevated behavior problem scores. Implications for practice, policy, and further research will be discussed.

### A school based and teacher led TF-CBT-intervention for reducing nightmares among war affected youth in Northern Uganda 17:45–18:00

#### J. Schultz Norwegian Centre for Violence and Traumatic Stress Studies, Oslo, Norway

The objective of this study is to explore the change in nightmares in former child soldiers and war-affected youth in Northern-Uganda attending a one-year school program. Due to conflict, the adolescent have dropped out of the ordinary education system. Pupils with posttraumatic nightmares were invited to participate in a school based short term intervention led by local teachers. The pupils went through screening interviews, baseline was established and they were interviewed 4 months post intervention. The intervention consisted of 4 group sessions and 4 individual sessions based on a short version of trauma-focused cognitive behavior therapy (TF-CBT) focusing on: psycho education, exposure, trauma narrative, breathing and relaxation exercises, and future orientation. Thirty one learners participated in the intervention and the majority had a significant reduction in their number of nightmares measured 4 months post intervention. Through the presentation, there will be a discussion of 1) cultural issues of trauma with a special focus on psychoeducation and 2) adaptions of evidence-based practice in order to facilitate the program to be led by teachers. To what extent and in what way can teachers and schools facilitate a trauma-focused intervention targeting nightmares and posttraumatic stress reactions (PTSD).

## ORAL, JUNE 7 HALL GLORIA

## Morning Responding to disasters
*Symposium: Education of helpers in preventing and treating conditions after disasters*

### Early intervention and treatment programs in public health services—implementation, philosophy, and strategy. How does this specific pedagogical approach strengthen professional content and maintenance of the competence in trauma and suicide prevention? 10:00--10:15

#### T. Anstorp, K. Silvola, N. P. Reinholdt and T. Araldsen Regional Centre for Violence, Trauma and Suicide Prevention, Region East (RVTS Øst), Oslo, Norway

The Regional Centre for Violence, Trauma and Suicide Prevention, Region East (RVTS Øst) is located at Oslo University Hospital and financed by The Norwegian Directorate of Health. It covers four counties with almost two million inhabitants and is one of five similar units in Norway. These centers have two main tasks: to increase competence among professionals in public health and other relevant public institutions working with persons affected by trauma and suicide, and to support local and regional cooperation and professional networking. We use RVTS Ost as an example to highlight challenges in training programs in public services. Increased competence must be visible in practice as altered professional behavior and/or as better organization of administrative or other frames. On the individual-level, competence is conceptualized as interaction between knowledge, attitudes, and skills. This view has a direct consequence to the organization of training and to the pedagogical approach. The group size and length of the intervention process as well as maintenance of the results are important elements. To transform complex knowledge into useful content is a constant challenge. The content has to be rich yet clear, deep yet practical, and comprehensive yet simple. Skills training and practice require balance between safety and challenge throughout the whole training process. On the organizational level, two models will be identified and presented in this workshop. To reach out for professionals in the primary health care and other related services, the training program has to connect to different administrations, whereas the in- and out specialist health care is under the same administration. The preliminary work with leaders is crucial in both situations but looks different in practice.

### What community caregivers with multidiciplinary background need in order to intervene succesfully after crisis and disasters to prevent long-term posttraumatic conditions? An educational program 10:15–10:30

#### K. Silvola, H. Herrestad, G. Nordmo and M. Kjolseth Braein Regional Center for Violence, Trauma and Suicide prevention, Region East (RVTS Øst), Oslo, Norway

How should communities prepare to effectively respond to a disaster that involves their inhabitants? This became a nationwide issue in Norway after the terror of 22nd July, 2011. The terrorist attack on the political camp at Utøya affected hundreds of young people from all over the country. At the same time, The Norwegian Directorate of Health had just presented new guidelines for psychosocial care after crises and disasters. The Regional Centres for violence, trauma and suicide prevention (RVTS) got the task to help communities to implement these guidelines. As a first step, RVTS Øst (Region East) has designed a standardized learning experience “Crises, trauma and sorrow”-the basic education in psychological first aid. The workshop is presented during two consecutive days. Each workshop requires minimum of two trainers and 24 participants. Best results are achieved with a mix of professionals from different organisations who have similar tasks in early intervening. A special target group is crises intervention teams in municipalities. A web education program “When the crisis strikes” is used as a warm-up and teaching of theory so that more time is left for skills training, interactive teaching, and networking. Our goal is to teach both the processes behind acute stress reactions as well as stabilization techniques, so that participants get both knowledge and skills to enhance resilience and to prevent long-term harmful effects. Communities are supported in making written guidelines for their work. Distributing the workshop to large numbers of professional is best done through regional training recourses together with the staff at RVTS Ost. A special focus on trainer's level of the program is under planning. The piloting phase lasts until summer 2013. By then, approximately 300 professional helpers have participated. This presentation focuses on the pedagogical design, thematic content, and implementation strategy. Workshop evaluations are summarized.

### Integrating more expert trauma treatment into the regular health care system—experiences from the “Norwegian Model” 10:30--10:45

#### T. Anstorp Regional Resource Centre for Violence, Trauma and Suicide Prevention, Region East (RVTS Øst), Oslo, Norway

In Norway, traumatized people often failed to get good enough help from the health services. Only a few therapists, most of them working in private practice, were treating complex trauma successfully. Fifteen years ago, a group of psychologists decided to organize educational programs to strengthen the understanding and treatment of severe traumatization. How could trauma knowledge be a part of ordinary health practice? The prevalence of complex traumas in psychiatric populations is so high that the general health service really needs to develop competence in this field. Six years ago, the organization of trauma educational programs was taken over by the newly established Regional Resource Centre of Violence, Trauma and Suicide Prevention (RVTS Øst). This center is financed by The Norwegian Directorate of Health. During the last six years, several thousand specialized health care workers have attended to one-year intensive training followed by a four-year period of workshops and supervision. Both out- and in-patient services have been very enthusiastic about this program, in which leaders are especially encouraged to attend. Our slogan is: “Building competence involves change in both the organization of health systems as in clinical practice”. The workshop will describe how the trainings were organized as well as share some of the thematic content. It will describe system changes that have been made after massive training interventions. Furthermore, it will describe how we have developed a trauma perspective in which therapists of many theoretical backgrounds feel comfortable. They all share the common language of phase-oriented treatment. The tables are turned. From a few activist therapists, to health systems creating structured treatment programs for many different categories of traumatized patients—supported by The Directorate of Health.

### Strenthening the support and care of Norwegian war veterans. Educational programs integrating trauma understanding and experiencies from soldiers in active duty and war veterans 10:45–11:00

#### N. P. Reinholdt and L. Lyster
Regional Resource Centre for Violence, Trauma and Suicide Prevention, Region East (RVTS Ost), Oslo, Norway

The Regional Recourse Centre for Violence, Trauma and Suicide Prevention-Region East (RVTS Ost) has a mission from the Norwegian Directorate of Health to perform different activities in order to strengthen the support and care for Norwegian war veterans. Since 1946, approximately 100,000 Norwegians have contributed in more then 40 peace keeping and military operations in four continents. Most of these veterans return with valuable experiences they can take advantage of in their personal and professional lives. However, a significant amount of soldiers have engaged in severe combat actions experiencing stress disorders and psychological distress. The project aims to build bridges between the Norwegian Defence at different levels and the civilian care system. Families of soldiers who are participating in international operations are an important target group. One of the aims is to prevent stress disorders among new veterans and their families and provide better treatment and care for those who already have difficulties. Extensive educational activities are planned for target groups who are responsible for the follow-up of our veterans. There are established educational programs for central groups in the health care systems. These programs use actively experiences from soldiers in duty, veterans, and their relatives. A decisive success criterion in order to be able to increase the knowledge within the civilian supporting systems is realistic understanding of the military reality and the challenges veterans and their families have to deal with. We have published a number of articles to spread knowledge about how foreign assignments can affect everyday life for the veterans and their families. This presentation covers different parts of the project and describes a structure for regional resource networks.

## Responding to disasters
*Symposium: Psychological responses following major incidents in the UK*

### Trauma risk management-an organizational response for police officers responding to a multiple shooting 11:45–12:05

#### E. Hunt, N. Jones, V. Hastings and N. Greenberg Academic Centre for Defence Mental Health (ACDMH), King's College London, England, UK

*Introduction:* A major incident involving multiple fatalities occurred in Cumbria, England on June 2nd, 2010. It was one of the worst crimes involving firearms in British history. The Cumbrian Constabulary deployed an organizational peer support response for personnel involved known as Trauma Risk Management (TRiM). *Aim:* To examine data gathered during the TRiM process to evaluate the relationship of the intervention to sickness absence. *Method:* Seven hundred and twenty-three police officers and civilian support staff were identified from incident databases; details were gathered regarding exposure to the murders, sociodemographic information, and type of TRiM intervention, including an assessment of the psychological risk of the individual developing a trauma-related mental health problem. Cumulative sickness absence in the two months following the murders was used as a proxy for mental health status. *Results:* A total of 42.1% of officers received a TRiM intervention; those who did reported the highest levels of potentially traumatic exposure. The majority of psychological risk indices had reduced from the period of first evaluation to those evaluated one month later. Greater traumatic exposure was associated with longer sickness absence lengths. Higher TRiM risk assessment scores were significantly associated with receiving a supportive intervention for mental health difficulties; there was no evidence that the TRiM process itself affected mental health status. *Conclusion:* In this study of TRiM deployed within a police force responding to a major critical event, we found that it offered a way of mounting a structured response for the police officers involved. Our data suggest that TRiM may offer a way of identifying those at risk so that they can be offered early psychological treatment.

### A psychosocial response to the Cardiff hit-and-run major incident October 19, 2012 12:05–12:25

#### N. Kitchiner^1^, N. Roberts^1^, T. Vick^2^ and J. Bisson^1^

^1^Cardiff University, Cardiff, UK; ^2^University Hospital of Wales, Cardiff, UK

On October 19th, 2012, a male driving a van murdered a mother of 3 and tried to kill 13 more in hit-and-run rampage in Cardiff, UK. More than a dozen people were injured in the incident. Two adults were left in a critical condition and five children, all received inpatient treatment at the University Hospital. This unusual incident triggered the local NHS Cardiff and Vale, Psychosocial Disaster Management Plan (PDMP). This paper will describe how the PDMP has been developed and tested over the past 10 years via mock table top scenarios prior to this incident (Bisson et al., 2010). The strengths and weaknesses of our PDMP have been systematically highlighted when applied to this incident. Data will include: how many victims and witnesses responded to a targeted contact via the police via a specifically designed psychosocial information leaflet. Scores from individuals who completed the trauma screening questionnaire (Brewin et al., 2002) sent with the information leaflet and pre and postdata from individuals who were offered early trauma focused psychological therapy. Implications for services offering a psychosocial response following a major incident will be discussed with recommendations for both clinicians and researchers (Meewisse, Olff, Kleber, Kitchiner, & Gersons, 2011).

References

Brewin, C., Rose, S., Andrews, B., Green, J., Tata, P., Mcevedy, C., et al. (2002). Brief screening instrument for post-traumatic stress disorder. *BJP*, *181*, 158–162. doi: 10.1192/bjp.181.2.158.

Bisson, J. I., Tavakoly, B., Witteveen, A. B., Ajdukovic, D., Jehel, L., Johansen, V. J., et al. (2010). TENTS guidelines: Development of post-disaster psychosocial care guidelines through a Delphi process. *BJP*, *196*, 69–74. doi: 10.1192/bjp.bp.109.066266.

Meewisse, M. L., Olff, M., Kleber, R., Kitchiner, N. J., & Gersons, B. P. R. (2011). *Journal of Traumatic Stress*, *24*(4), 405–413.

### Planning for the psychosocial and mental health needs of people affected by emergencies: the Scottish model 12:25–12:45

#### G. Moreton Rivers Centre for Traumatic Stress, Edinburgh, Scotland

Scotland has recently adopted new guidance to assist in planning for the psychosocial and mental health needs of people affected by emergencies. The guidance was produced by the Rivers Centre in consultation with a wide range of Scottish, UK, and international sources of expertise and is broadly based on the 2009 document, “Guidance for Responding to the Psychosocial and Mental Health Needs of People Affected by Disasters or Major Incidents” which was written by the team involved in the North Atlantic Treaty Organisation (NATO) guidance and the European Network for Traumatic Stress (TENTS) program. The consultation process benefited greatly from the input of survivors of previous emergencies. The Scottish guidance is based on the model of psychological first aid (PFA) and outlines a number of core principles which should inform the interagency response, including a timeline and tasks for the responding agencies. This paper will summarize the current understanding of best practice in disaster mental health and will present the Scottish model. We will share the lessons learned in the development of the guidance and in the process of implementing it, including plans to deliver training in PFA for first responders and seeking agreement on data-protection issues. Lessons learned will be illustrated with examples of the challenges associated with turning a document into something that responders will remember and find helpful in the emergency context.

## Afternoon Evidence-based practice on trauma
*Workshop: Early Interventions based EBP for reducing the ASR and preventing PTSD*

### Scientifically based early interventions for reducing the ASR and preventing PTSD 15:15–15:35

#### M. Farchi^1^ and Y. Gidron^2^

^1^Department of Stress, Trauma aand Resilience Studies, Tel-Hai College, Israel; ^2^Faculty of Medicine and Pharmacy, Free University of Brussels (VUB), Belgium

**Theory, evidence, and demonstration**

This proposed symposium will provide the scientific rationale, the empirical evidence, and practical skills in three early interventions for treating the acute stress reaction (ASR), for preventing posttraumatic stress disorder (PTSD), and for improving recovery from traumatic events. First, we provide the neuropsychological rationale for the memory structuring intervention (MSI), which aims to help traumatized people shift the processing of their memory from an implicit, limbic, and emotional/somatic manner to an explicit, frontal-lobe and cognitive manner. The MSI prevented PTSD mainly in women, in two small randomized trials. We recently added to it vagal nerve breathing, to reduce sympathetic hyperactivity, and provide evidence for its ability to reduce ASR in a third trial. The second intervention tested on patients in an emergency room is based on stress and coping research, where coping self-efficacy prevents PTSD. This stress management (SM) intervention was also found to reduce ASR in patients attending an emergency room. The SM teaches patient's emotion-focused coping (vagal breathing) and problem-focused coping (identifying and planning active coping). Finally, the third intervention is psychological inoculation (PI), which aims to remove people's cognitive distortions and barriers that prevent adaptation to traumas. In PI, we expose people to challenging sentences that reflect an exaggerated form of their barriers or distortions (the “vaccine”), they learn to refute (the “antibody” response). PI was found to increase physical activity, reduce barriers for condoms, reduce road hostility, reduce fears during a global flu epidemic and reduce helplessness under missile attacks, better than various control conditions. We shall demonstrate each method and its rationale, the evidence for its efficacy, ask participants to practice, and provide criteria for choosing when to use each intervention.

### Memory structure intervention (MSI) for reduction of ASR symptoms and PTSD prevention 15:35–15:55

#### Y. Gidron^1^ and M. Farchi^2^

^1^Faculty of Medicine and Pharmacy, Free University of Brussels (VUB), Belgium; ^2^Department of Stress, Trauma and Resilience Studies, Tel-Hai College, Israel

*Background:* Currently, research shows very less evidence for early interventions that prevents posttraumatic stress disorder (PTSD), and less systematic work has been done to reduce the acute stress response (ASR) after traumas. We developed the memory structuring intervention (MSI) and recently added to it vagal breathing (VB). The MSI tries to shift trauma processing from implicit, limbic, and affective-somatic manners to explicit, frontal-lobe and cognitive manners, as these predict better prognosis. Furthermore, chronological organization of traumatic memories, labeling sensations, and providing causality may help produce such processing shift. Yet, since the MSI was ineffective in past for men, in whom sympathetic hyperactivity predicts PTSD, we added VB. We provide the scientific background, research evidence for the effectiveness, and will demonstrate the MSI+VB. *Method:* We conducted to date three randomized controlled trials (RCTs): first in 17 postaccident victims, second in 34 accident victims, and third in 124 patients attending an emergency room. The first two RCTs included PTSD measures, while the last RCT included enough patients only for assessing the ASR. Patients were randomized to MSI or to supportive listening (RCT1 and RCT2) or to MSI+VB versus supportive listening versus stress management (RCT3; see presentation 2). In the MSI+VB, patients learn slow paced breathing, and to chronologically organize their memory, label sensations, and provide causality for event elements. *Results:* In RCT1, PTSD symptoms at 3 months were reduced more in the MSI than in the control group, while in RCT2, this occurred only in women. In RCT3, the MSI+VB reduced ASR symptoms, while not in controls. *Conclusions:* The MSI+VB reduced ASR symptoms, and the MSI alone may prevent PTSD symptoms in women. A large RCT will test the MSI+VB in relation to ASR and PTSD.

### A stress-management-derived intervention for ASR reduction and PTSD prevention: theory, evidence, and demonstration 15:55–16:15

#### M. Farchi^1^ and Y. Gidron^2^

^1^Department of Stress, Trauma and Resilience Studies, Tel-Hai College, Israel; ^2^Faculty of Medicine and Pharmacy, Free University of Brussels (VUB), Belgium

Theory, evidence, and demonstration *Background:* Currently, research shows very less evidence for early interventions that may prevent posttraumatic stress disorder (PTSD), and less systematic work was done to reduce the acute stress response (ASR). We developed a stress management (SM)-based intervention. The SM intervention teaches people to perform emotion-focused coping by vagal breathing (VB) and problem-focused coping (PFC) by empowering patients to focus on successful activities they did or plan to do during and after the event. These are aimed at increasing coping self-efficacy, a predictor of better prognosis after trauma. We provide the scientific background, research evidence for the effectiveness, and will demonstrate the SM intervention. *Method:* We conducted a randomized controlled trial (RCTs), with 124 patients attending an emergency room. Patients were randomized to SM or to supportive listening or to memory structuring intervention (explained in presentation 1). We focus on ASR symptoms-pain, anxiety, and heart rate as outcomes. The SM included VB and asking patients what they did during the event, in the emergency room and what they can do later, to help themselves. *Results:* The SM reduced ASR symptoms, while the control condition did not. These were seen in both genders. *Conclusions:* The SM reduced ASR symptoms. We are running a large RCT to test the effects of SM on the ASR and PTSD prevention.

## Responding to disasters
*Symposium: Aftermath of Van earthquake - psychosocial interventions, health workers and NGOs*

### Aftermath of Van earthquake: psychosocial interventions 16:45–17:05

#### T. Aker Kocaeli University, Kocaeli, Turkey

Psychosocial support and interventions in Van were implemented by 184 mental health workers. Psychosocial services included the day nursery and etude facilities, analyzing the needs and resources, group workshops, short-term group psychotherapy, psychoeducation, social activities, psychiatric evaluation, and therapy and activities for supporting the volunteers. Psychosocial support was provided to a total of 14,603 people in 11 temporary settlements. Psychosocial support was also provided to 2,207 victims of disaster who moved out of Van in collaboration with the Ministry of Family and Social Policies and the International Organization for Migration. Training programs were organized for health care professionals working at the epicenter to equip them with the necessary know-how in psychosocial support provision under disaster conditions. A basic level trauma training program for improving the self-efficiency of individuals was provided to the psychosocial workers of organizations working in the field of trauma in collaboration with the Ministry of Family and Policies, UNICEF and Disaster, and Emergency Management Presidency.

### Aftermath of Van earthquake: health workers 17:05–17:25

#### H. S. Kalkan DiyarbakIr Ergani State Hospital, DIyarbakIr, Turkey

Health professionals in disaster areas form a unique group of at-risk individuals. They have two hats to wear: they are both victims and relief workers at the same time. Van was no exception to this. The health workers in Van had been deprived of their homes similar to anybody else in Van at the time and they had been angst ridden with the safety of their loved ones; however, they were still expected to serve the earthquake victims as if they had not been victimized themselves. They were expected to overcome an immense work load and heal others without having been given the chance to heal, pointing to the importance of psychosocial support interventions targeting the health workers.

### Aftermath of Van earthquake: NGOs 17:25–17:45

#### E. Kirmizi Alsan Kocaeli University, Kocaeli, Turkey

The Van-Erciş and Van-Edremit earthquakes have shown once more that a healthy cooperation between NGOs and the governmental bodies is necessary for the provision of psychosocial support to the earthquake victims. The presence of a roof-top organization such as the Union of Disaster Psychosocial Services (UDPS) that consist of the Turkish Psychiatric Association (TPA), Turkish Red Crescent Society, Turkish Association for Child and Adolescent Psychiatry, Turkish Psychological Association, Turkish Psychological Counseling and Guidance Association, and Turkish Association of Social Workers has proven to be an essential ingredient for the formation of a healthy and productive collaboration between various NGOs and the governmental bodies.

## ORAL, JUNE 7 HALL LADY G

## Morning Cultural issues and trauma
*Workshop: Research on cultural competence for treating posttraumatic stress*

### Cultural competence in treating traumatic stress: towards a joint grant proposal 10:00–10:20

#### J. Knipscheer and R. Kleber Arq Psychotrauma Expert Group/Department of Clinical and Health Psychology, University Utrecht, Utrecht, The Netherlands

Mental health care for ethnic minorities with PTSD is often associated with low efficiency. Psychotherapeutic interventions, such as CBT and EMDR, are not evidence-based for ethnic minority groups, (Crumlish & O'Rourke, 2010; Palic & Elklit, 2011) and treatment as usual is characterized by high numbers of no show and drop out with substantial adverse psychosocial and economic consequences. Since a large and growing part of trauma victims concern people from ethnic minority groups, the key question for many mental health care professionals nowadays is: Are evidence-based interventions applicable and effective for affected ethnic minority groups and if not, what should be the alternative? Culturally adapted interventions seem to be more effective than regular interventions for primary measures of psychological functioning (Benish, Quitana & Wampold, 2011). However, the robustness of the evidence is marginal and only available for specific ethnic subgroups in the USA. In this workshop, two questions are central: (1) what characterizes a cultural competent trauma treatment and (2) how can the effectiveness be determined? We will introduce the cultural competence program that has been developed within *Foundation Arq* (the national expert centre for treatment of, and research into, the psychosocial consequences of trauma in The Netherlands) including cultural competence training for therapists and application of culture sensitive treatment modules. We will invite the participants to share their ideas and experiences concerning developing and studying cultural competency, and to explore possibilities of working together in creating a consortium. We aim to leverage our resources by collaborating with complementary partners on joint grant seeking regarding research on the effectiveness of cultural competent interventions for PTSD. The symposium will be highly interactive and active involvement of the participants will be very much appreciated.

References

Benish, S. G., Quintana, S., & Wampold, B. E. (2011). Culturally adapted psychotherapy and the legitimacy of myth: A direct-comparison metaanalysis. *Journal of Counseling Psychology*, *58*, 279–289. doi: 10.1037/a0023626

Crumlish, N., & O'Rourke. A. (2010). Systematic review of treatments for Post-Traumatic Stress Disorder among refugees and asylum-Seekers. *The Journal of Nervous and Mental Disease*, *198*(4), 237–251.

Palic, S., & Elklit, A. (2010). Psychosocial treatment of posttraumatic stress disorder in adult refugees: a systematic review of the literature of prospective treatment outcome studies and a critique. *Journal of Affective Disorders*, *131*(1–3), 8–23.

### PTSD research among migrants and refugees 10:20–10:40

#### R. Kleber Arq Psychotrauma Expert Group/Department of Clinical and Health Psychology, University Utrecht, Utrecht, The Netherlands

Ethnic minorities form groups at risk for developing PTSD (Drogendijk et al., 2003; Norris et al., 2002) with prevalences varying from 20% for labor migrants (Lindert, Ehrenstein, Priebe, Mielck & Brhler, 2009) to more than 40% for refugees and asylum seekers (Fazal, Wheeler & Danesh, 2005; Toar et al., 2009). Posttraumatic symptoms may differ extensively between western and non-western groups with somatic complaints, hostility, and embitterment being more prominently articulated among migrants and refugee groups. The so-called “*condicin migrante*” may account for much of the variability in symptom presentation and PTSD development.

### Cultural competence in trauma treatment: how can we measure if it works? 10:40–11:00

#### J. Knipscheer Arq Psychotrauma Expert Group/Department of Clinical and Health Psychology, University Utrecht, Utrecht, The Netherlands

Trauma-focused CBT and EMDR are evidence-based treatments of choice for treating PTSD (Bisson, 2009; NICE, 2005), however the external validity of RCT's is low as ethnic minority patients concern less than 1 percent in efficacy trials. The question is whether evidence-based interventions are applicable and effective among affected migrants and refugees. Cultural competencies (key notions are knowledge, attitude, and skills) as well as specific culture sensitive interventions (e.g., psycho-education, relaxation techniques, a contextual and systemic perspective, explicit attention to practical, societal and physical factors, affect tolerance and “empowerment”) have been suggested to bridge the (cultural) gap between western therapists and non-western patients. Yet, up to now, the effectiveness of increased cultural competence of therapists in reducing drop-out of treatment and improving the success rate has not been studied. Methods to increase cultural competency (e.g., training therapists) and to determine the effectiveness will be discussed.

## Effects of trauma on families and children
*Symposium: Effects of war trauma in family life: From etiology to intervention*

### Parental care moderates the association between trauma and mental health in Tamil children in northern Sri Lanka 11:45–12:00

#### V. Sriskandarajah, F. Neuner and C. Catani Bielefeld University, Bielefeld, Germany

Traumatic experiences are common in the North and East of Sri Lanka, a region devastated by a civil war lasting for more than two decades and the Tsunami catastrophe in 2004. The Tamil population, in particular the children, now have to face the widespread consequences of years of trauma due to war and disaster. Research, so far, has mainly focused on the impact of these traumatic experiences on individual mental health and has found a high prevalence of posttraumatic stress disorder (PTSD) and depression in affected adults and children. However, little is known about the multifaceted effects of war on family life, on parenting behavior, and the use of violence against children. Against this background, we conducted an epidemiological survey with families in three regions of Northern Sri Lanka. The regions differed in their level of war and Tsunami exposure. Structured clinical interviews were conducted separately with children and their caretakers and included standardized measures for the assessment of traumatic events, mental health, and parenting behavior. Interviews were carried out by previously trained local counselors. This presentation focuses on the child sample only (*N*=359). Findings showed that children from the region not affected by war or the Tsunami, report less family violence than children from more affected regions. Depending on the trauma exposure in the specific area, PTSD prevalence varied from 1.7 to 33.6%. In a regression model (*R* ^*2*^=0.44) family violence, war exposure and parental care were significant predictors of child mental health. Most importantly, we found a moderating effect (β=−0.11) of parental care on the relationship between war exposure and mental health. War exposure leads to mental illness, only if parents are perceived as being less caring. These results are particularly relevant for the development of targeted psychosocial interventions for war torn families.

### Does war lead to violence against children? Findings from a multi-informant survey in Northern Uganda 12:00–12:15

#### R. Saile, F. Neuner, V. Ertl and C. Catani Bielefeld University, Bielefeld, Germany

After 20 years of civil war in Northern Uganda, families are challenged by a multitude of individual level and family level risk factors for violence within the family. Parents who have been exposed to high levels of war-related traumatic events, and whose psychological functioning is impaired by posttraumatic symptoms and alcohol-related problems, may be more prone to engage in aggressive parenting behaviors towards their children. On a family system level, inter-parental violence, insecure living conditions, and changes in the family structure may contribute to more violent parent-child interactions. The current study was located in seven heavily war-affected rural communities in Northern Uganda, where experienced local therapists interviewed an exhaustive sample of second-grade students and their male and female guardians using standardized clinical questionnaires. The aim of the study was to identify individual level risk factors for self-reported aggressive parental behaviors as well as family level risk factors for child-reported experiences of family violence. Analyses are based on self-report data from 365 female guardians, 304 male guardians, and 283 triads including both guardians and the index child. The strongest predictors of self-reported aggressive parenting behaviors towards the child were guardians' own experiences of childhood maltreatment followed by female guardians' victimization experiences in their intimate relationship and male guardians' PTSD symptoms and alcohol-related problems. Regarding children's self-report of family violence, environmental variables such as general traumatic events and violence between adults in the household predicted children's experience of maltreatment. Parental variables such as female guardians' history of childhood maltreatment, female guardians' exposure to traumatic war events, and male guardians' PTSD symptom severity level increased children's risk for the experience of family violence. The current findings suggest that in a context of organized violence, an intergenerational cycle of violence persists that is exacerbated by female guardians' revictimization experiences and male guardians' psychopathological symptoms.

### Families in the context of war—behavioral observations as a method to assess parenting in Northern Uganda 12:15–12:30

#### J. Moellerherm^1^, R. Saile^1^, E. Wieling^2^, F. Neuner^1^ and C. Catani^1^

^1^Bielefeld University, Bielefeld, Germany; ^2^University of Minnesota, Minneapolis, MN, USA

A growing body of literature has shown that war-affected families are at an increased risk for family violence. Ineffective parenting has been hypothesized as a mediating mechanism underlying this association. Recent studies mainly relied on standardized questionnaires to gain information about parenting practices following war exposure. However, quantitative data based on self-reports of behavior is subject to a number of limitations. Moreover, hardly any research has been conducted to systematically study family interactions in non-western countries affected by war and conflict using alternative methods of assessment. Considering the limitations of self-report measures, we used a combination of behavioral observations and quantitative methods to get a unique insight into parenting in Northern Uganda, where virtually the entire civil population has been severely affected by 20 years of civil war. Between April 2012 and December 2012, interactions of 100 mothers and their 6- to 12-year-old children (*M*=8.96, *SD*=1.90) were observed during five structured culturally adapted tasks. These activities included an emotion-focused discussion of one positive and one negative event from the child's life as well as one problem solving task. Activity-oriented tasks consisted of culturally adapted games. Each task took 5 min and was recorded on video. In addition to behavioral observations, mothers and their children participated in separate interviews that were based on standardized questionnaires. The questionnaires captured socio-demographic information, previous traumatic experiences, psychopathology, and parenting behavior. In the current presentation, we will focus on the process of adapting and implementing behavioral observation tasks in Northern Uganda. Further, we will present preliminary findings on the cultural validity of parenting dimensions as well as potential associations of parenting behavior with socio-economic background, war exposure, and mental health in children and their mothers.

### Pilot implementation of a parenting and family group intervention with Acholi mothers in Northern Uganda 12:30–12:45

#### E. Wieling^1^, C. Mehus^1^, J. Moellerherm^2^, F. Neuner^2^ and C. Catani^2^

^1^University of Minnesota, Minneapolis, MN, USA; ^2^Bielefeld University, Bielefeld, Germany

Preliminary results of multi-method data will be presented for this pilot study exploring the feasibility of implementing a 9-session manualized parenting/family intervention called “Enhancing Family Connection (EFC).” Two groups (*N*=14) were conducted in summer 2012 with Acholi mothers. Each session consisted of meeting for 3 hours, two times per week. EFC was conducted by two co-facilitators, two trained interpreters, and a support team. Pre-, post- and 4-month follow-up data were gathered for mothers and identified a focal child between the age 9 and 13 using measures to assess for mental health status, parenting practices, and child outcomes. Specifically, study goals were to evaluate 1) acceptability, 2) implementation, and 3) limited efficacy and effectiveness. Reports from recent studies in Uganda indicate that the civil war has resulted in individual-level consequences as well as deleteriously impacting the family system, including higher levels of partner violence, risk of alcohol problems in males and adverse parenting/child abuse. These findings endorse an ecological understanding that trauma and its sequelae do not happen at an isolated and individual-level. Rather, reciprocal and interdependent influences within family systems must be taken into account to the greatest degree possible. The EFC intervention was adapted from the widely established evidence-based Parent Management Training-Oregon Model (6 sessions), and also integrated trauma psychoeducation (1 session) and intergenerational transmission of violence models that include associated relational adversities (2 sessions). It is noteworthy that mothers attended all group sessions and three-wave data were collected for all mother-child dyads. Ongoing analyses consist of examining psychological and parenting measures, coding of mother-child observational structured interaction tasks, and content analysis of individual qualitative interviews. Preliminary results indicate that EFC was acceptable and can be implemented with Acholi mothers. There is early evidence that although parenting practices seemed to change for most mothers, prolonged intervention with additional support is necessary.

## Afternoon Miscellaneous
*Symposium: Struggle of paradigms in a system of trauma care in Georgia*

### Trauma-informed mental health policy: how far can we go to close treatment gap? 16:45–17:00

#### N. Makhashvili Global Initiative on Psychiatry-Ilia State University, Tbilisi, GA, USA

There are currently an estimated 40 million persons that have been forcibly displaced by armed conflict, the vast majority of whom live in low- and middle-income countries (LMICs). They include over 26 million internally displaced persons (IDPs) who remain within the borders of their countries. These populations affected by conflicts commonly experience high levels of exposure to traumatic events and, consequently, significantly elevated levels of posttraumatic stress disorder (PTSD), depression, somatoform disorders, anxiety, etc. Poor living conditions, loss of livelihoods, reduced social support, and other stressors aggravate the burden of mental disorders. Recent study in Georgia provides data on high prevalence of PTSD, depression, anxiety and other disorders among IDPs, significant increase in functional disability and lack of mental health (MH) services. This evidence highlights the importance of a comprehensive approach to tackling trauma-related conditions to help ensure more effective interventions. Nevertheless, a lack of political support, inadequate management, and overburdened mainstream health services are hampering the development of coherent MH systems. The paper explores the current formal MH care system in Georgia, discusses the national program for MH and challenges of on-going reform, and indicates at wide treatment gap. The important step towards providing well-considered MH care is development of a strategic policy that will guide MH reform. However, how far the mainstream MH policies could incorporate services for trauma-affected large groups? What are the best practices in LMICs that can influence local policies and practice? The paper discusses the fresh data from on-going qualitative study and tries to conceptualize the chain of integrated care according to WHO model of 'Optimal Mix of Services' (2007). It proposes concrete strategies that should be implemented to tackle mental disorders and associated disability, as well as specifying the targets to be achieved by policy-makers.

### Life with ambiguous loss—re-constructing identities: working with families of the persons missing as a result of wars in Georgia 17:00–17:15

#### S. Tabaghua, N. Kiladze and L. Tsiskarishvili Georgian Centre for Psychosocial and Medical Rehabilitation of Torture Victims, Tbilisi, GA, USA

Since 2009 the Georgian Center for Psychosocial and Medical Rehabilitation of Torture Victims—GCRT in cooperation with the International Committee on the Red Cross—ICRC has been implementing a program of psychosocial assistance to family members of the persons missing from the war of 1992–1993 in Abkhazia—a breakaway region of Georgia. In four cities of Georgia support groups are running. The program involves psychologic, legal and economic elements. The authors will present the concept and structure of the work; will discuss implemented group and individual interventions—importance of legal awareness workshops, as well as small business grants program available for the families. Apart from these peculiarities of working with ambiguous loss, ascribing meaning to life after the loss and reconstructing identity will be reflected upon. The effectiveness of the program will be analyzed; results of outcome evaluation shared; the challenges and barriers discussed as well as future plans in further developing the program will be presented.

### Traumatizing treatment? Understanding the extent and nature of problems faced by children with mental health disorders in Georgia 17:15–17:30

#### N. Agapishvili Georgian Association for Psychosocial Aid Ndoba, Tbilisi, GA, USA

As a result of poor management of the mental health field in Georgia, majority of children and adolescents with mental disorders do not receive adequate treatment for their condition and mental disorders impose a heavy burden on their families, driving them under the poverty line. Presented study addresses two specific problems existing in Georgia that hinders children and adolescents from realization of their rights for mental health: 1) absence of stand alone strategy and programs for adequate management of mental health disorders for children and adolescents and 2) low awareness of the decision makers of the country and also public on the burden associated with mental health problems. Study of services funded under the state programs with regard to child and adolescents mental health was conducted in 19 psycho neurologic dispanseries, six psychiatric hospitals in 2012. Focus groups and structured interviews were used as instruments of study with service providers: medical staff, psychologists, nurses; service beneficiaries and parents; with mental health care experts. Findings of study: existing problems, inadequacies of treatment, treatment gaps, lack of needed services, and underestimation of specialized services, especially trauma services for children and adolescents etc will be presented. Working directions for improvement of child and adolescents mental health care field performance will be presented as well. Case demonstrating inadequate medical treatment provided by state funded outpatient service, alongside with negligence of child's real needs resulted in trauma-related conditions will be presented.

### “The Caucasian Chalk Circle”—two families struggling for a 2 years old girl: reenactment of family trauma 17:30–17:45

#### J. D. Javakhishvili^1^, N. Burduli^2^, N. Kuchukhidze^2^ and K. Mgebrishvili^2^

^1^Foundation Global Initiative on Psychiatry-Tbilisi, Ilia State University, Tbilisi, GA, USA; ^2^Family and Child Care Centre, Georgian Centre for Rehabilitation of Torture Victims, Tbilisi, GA, USA

The paper presents a case of multidisciplinary work with a 2-year-old girl put into situation similar to that described by Bertold Brecht in his famous “Caucasian Chalk Circle”: two families struggle with each other for obtaining full guardianship and therefore, right to up-bring the child and live together. The dispute split her between the two families and turned into a dehumanized object of an ongoing conflict. In the course of psychotherapeutic treatment the situation was analyzed by the child's mother as a function of her family trauma. The paper describes a strategy of case management by the multidisciplinary team and explores factors of success; namely, how putting the child into the role of “patient” was avoided during management of the case, how impartiality towards the two sides engaged in the protracted conflict was achieved and maintained; how the initial “facade” treatment request to examine, diagnose and treat child formulated by biological mother's family was transformed into demand for psychotherapeutic help for biological mother and the whole family; how in the course of family counseling ongoing abnormal situation was linked with the trauma of biological mother's family and considered as re-enactment of her mother's traumatic experience which put her in front of the dilemma to choose between two parents in her childhood; guilt associated with her choice facilitated transmission of trauma to the next generation and its reenactment. In addition, issues related to counter transference developed among case managers due to extreme emotionality of the case and the ways of overcoming them will be discussed; lessons learned out of overall management of the case will be shared.

## ORAL, JUNE 7 HALL SAVOIA

## Morning Evidence-based practice on trauma
*Symposium: Internet-based interventions for trauma-related disorders in different populations: Treatment outcome and therapeutic alliance*

### Internet-based psychotherapy for posttraumatic stress disorder in war-traumatized Arab patients: a randomized controlled trial 10:00–10:20

#### B. Wagner^1^, J. Brand^2^, W. Schulz^2^ and C. Knaevelsrud^2^

^1^Medical University Leipzig, Leipzig, German; ^2^Treatment Centre of Torture Victims, Berlin, Germany

*Objective:* Internet-based interventions for posttraumatic stress disorder have proved feasible and effective in Western countries. Their applicability and efficacy in war and conflict regions remains unknown. This study investigated the efficacy of a cognitive-behavioral Internet-based intervention for war-traumatized Arab patients, with focus on Iraq. *Method:* A total of 159 individuals with posttraumatic stress disorder participated in a parallel-group randomized trial. Participants were randomly allocated to a five-week treatment group (*n*=79) or a waiting list control group (*n*=80). The treatment group received two weekly 45-minute cognitive-behavioral interventions via Internet over a five-week period. The primary outcome was recovery from posttraumatic stress symptoms at posttreatment. *Results:* Posttraumatic stress symptoms were significantly reduced from baseline to posttreatment (intent-to-treat analysis) in the treatment group relative to the control group (*d=*0.68 to *d=*0.92). Additionally, patients in the treatment group showed greater reduction of comorbid depression (*d=*1.03) and anxiety (*d=*0.79) than did those in the control group. Treatment effects were sustained at 3-month follow-up. Completer analysis indicated that 62% of patients in the treatment group had recovered from posttraumatic stress symptoms at posttreatment versus 2% in the control group (odds ratio: 74.19, 95% CI [9.93-585.8], *p<*0.001). *Conclusion:* The results indicate that, even in unstable settings with ongoing exposure to human rights violations through war, people with posttraumatic stress symptoms benefit from a cognitive-behavioral treatment provided entirely through the Internet. This method of delivery could improve patients' access to humanitarian aid in the form of e-mental health services.

### EMMA and TEO: two e-health applications for stress related disorders 10:20–10:40

#### R. Banos^1^, S. Quero^2^, V. Guillen^2^, M. Moles^2^, M. A. Perez-Ara^2^ and C. Botella^2^

^1^Universidad de Valencia; ^2^Universitat Jaume I

The aim of this work is to present two e-health applications (EMMA and TEO) for the treatment of stress related disorders: Posttraumatic Stress Disorder, Adjustment Disorders and Complicated Grief. A common therapeutic element for these disorders is the exposure and processing of internal and external stimuli related to the negative event (e.g., Rosen, 2004). EMMA is a Virtual Reality applicationwhichadapts itself in a flexible manner to the particular needs of patients providingsignificant virtual environments capable to activate and enhance the emotional processing of the negativeevent.Results obtained so farin several case studies (Andreu-Mateu, Botella, Quero, Guillén & Baños, 2012; BaÞos et al., 2008; Botella et al., 2006, Botella, Osma, García-palacios, Guillén, & Baños, 2008) and in two controlled works(Andreu-Mateu, 2011; BaÞos et al., 2011; Quero et al., 2012) support the efficacy of this system for the treatment of stress-related-disorders. Furhtermore, other studies have shown high levels of expectations and satisfaction among patients and lower levels of aversiveness over the traditional condition (BaÞos et al., 2009; Botella, Baños, et al., 2006; Botella et al., 2010). More recently, our team has developed an Online Emotional Regulation System (TEO) which permits the patient to do the homework assignments at home over the Internet. This web-based system allows in a simple and effective way to create personalized therapeutic material to present to the patient (Quero, Botella et al., 2011). Preliminary data about the acceptability (Quero, Pérez-Ara et al., 2011) and efficacy (Quero et al., 2012) has already been obtained. TEO system falicites the patient’s treatment adherence and the therapist’s work in designing homework assigments.

### Predictors of treatment outcome in an Internet-based cognitive-behavioral therapy for posttraumatic stress disorder in older adults 10:40–11:00

#### M. Boettche^1^, P. Kuwert^2^ and C. Knaevelsrud^1^

^1^Center for Torture Victims, Freie University, Berlin, Germany; ^2^Ernst-Moritz-Arndt-University Greifswald, HELIOS Hanse Hospital Stralsund, Stralsund, Germany

*Background:* There have been important advances in the development of Internet-based treatment approaches for posttraumatic stress disorder (PTSD). However, data regarding which variables are uniquely linked to treatment response are rare. The aim of the study is to examine the influence of potential predictors on treatment outcome in Internet-based cognitive-behavioral intervention for PTSD in older adults. *Method:* In a manualized writing therapy, 72 older adults (M=70.9 years, SD=4.56) with war-related (subsyndromal) PTSD were examined at four assessment points (pre, post, three-, and six-month follow-up). Initial psychopathology, sociodemographic variables, and resource-related variables (self-efficacy; posttraumatic growth; locus of control) were examined as potential predictors of treatment outcome. *Results*: Multiple hierarchical regression analyses for the prediction of PTSD directly and 6 months after treatment identify PTSD at pretreatment (β=−0.52, *p*<0.001, β=−0.60, *p*<0.001, respectively), external (β=0.23, *p*=0.03, β=−0.26, *p*=0.02, respectively) and internal locus of control (β=−0.27, *p*=0.02, β=−0.24, *p*=0.03, respectively) and posttraumatic growth (β=−0.32, *p*=0.01, β=−0.20, *p*<0.10, respectively) as predictors. Already well-known variables in face-to-face therapy (e.g., gender, marital status, education) failed to be significant outcome predictors in this Internet-based treatment study. *Discussion*: The results demonstrate the relevance of resources for treatment outcome in older adults with PTSD and pave the way for future research whether prior additional resource-oriented treatment components can lead to a better therapeutic outcome in Internet-based cognitive-behavioral therapy.

## Evidence-based practice on trauma
*
Workshop: Psychodynamic trauma therapy Part I*

### Psychodynamic trauma therapy 11:45–12:05

#### R. Bering^1^, A. Elklit^2^ and K. Harold^3^

^
1^Center of Psychotraumatology, Alexianer Krefeld GmbH/University of Cologne, Cologne, Germany; ^2^South Danish University, Odense, Denmark; ^3^Duke University, Durham, NC, USA

The integration of various psychotherapeutic schools is crucial for the development of efficient trauma therapy. However, in the Guidelines of the ISTSS the psychodynamic approach is thought to be less efficient than cognitive-behavioral therapy, EMDR, or pharmacotherapy. This is mostly due to the lack of controlled studies. Nevertheless, the depth psychology has been of main importance to understand attachment disorders, the dynamic of stress response syndromes, and the development of trauma therapy. For this, our workshop addresses the following questions: how can we integrate psychodynamic trauma therapy (PTT) in order to guarantee a state-of-the-art treatment in psychotraumatology? In the meantime, there exist elaborated trauma-specific manuals based on PTT such as the multidimensional psychodynamic trauma therapy, the psychodynamic-imaginative trauma therapy, and the configurational analysis. Efficiency is proven for the PTT in single-case, clinical studies and controlled studies. According to the ESTSS General Certificate in Psychotraumatology, our workshop has three objectives: 1. We describe the essential components of PTT. 2. We discuss how PTT can be integrated into management plans that include trauma-focused psychological treatments also combined with pharmacotherapy and EMDR. 3. Finally, we compare and contrast PTT and the evidence for it with other treatments for PTSD. We conclude that basic principles of PTT should be integrated into trauma therapy.

### Inpatient unit treatment of posttraumatic stress disorder: combination of psychodynamic trauma therapy and the myoreflextherapy 12:05–12:25

#### R. Bering^1^, K. Mosetter^2^ and K. Muth^3^

^1^Center of Psychotraumatology, Alexianer Krefeld GmbH/University of Cologne, Germany; ^2^Gesellschaft fr Regulationsmedizin, Konstanz, Germany; ^3^Center of Psychotraumatology, Alexianer Krefeld GmbH, Germany

*Introduction:* Posttraumatic stress disorder (PTSD) often involves a variation of neuro-muscular syndromes, such as tension-headaches, shoulder-neck-pains, backaches, or abdomen-trouble. In order to provide broad treatment to victims of trauma, the Center of Psychotraumatology, Nordrhein, endorses psychodynamic trauma therapy (PTT) through a specialized physiotherapy (myoreflextherapy). The session includes two parts: first, the concept of the center is described briefly. *Method:* Empirical data on the therapeutic effects of inpatient unit treatment in a multiprofessional setting (*N=*96) are presented. The effect seizes of approximate 6-week treatment is between *d*=0.60 (SCl-90), *d*=0.78 (PTSS-10), *d*=0.90 (BDI), and *d*=1.0 (IES). Over this, we examined a subpopulation (*n*=30) symptoms of pain by semistandardized interviews and psychometric scales, such as SES, FESV, and FKB-20. *Results:* We confirmed our previous results and could provide evidence of moderate to large effects in treatment of PTSD-related and neuro-muscular syndromes. Over this, we compare our findings to other single-case, clinical studies and controlled studies based on psychodynamic, cognitive behavioral, and EMDR treatment. *Conclusion:* We conclude that the combination of PTT and myoreflextherapy is effective in treatment of PTSD and takes neuro-muscular symptoms associated with PTSD into account.

### Psychodynamic psychotherapy in clinical practice guidelines for PTSD: present status and future directions 12:25–12:45

#### H. Kudler Duke University, Durham, NC, USA

As first author of the ISTSS practice guideline on psychodynamic psychotherapy for PTSD and clinical champion for the USA Department of Veterans Affairs (VA) in the development of the VA/Department of Defense (DoD) Clinical Practice Guideline for the Management of PTSD, I have had first hand and “back room” experience in determining the quality and implications of research evidence for the efficacy of psychodynamic psychotherapy for PTSD. This presentation will consider the existing evidence base, biases that exist on all sides of this issue, current clinical practice guideline recommendations around the world and suggestions for future guideline development, research, and practice.

## Afternoon Evidence-based practice on trauma
*Workshop: Psychodynamic trauma therapy (part II) - making sense of repetitions and countertransference*

### Psychodynamic trauma therapy (part II): making sense of repetitions and countertransference 15:15–16:15

#### R. Ørner^1^ and L. Wittmann^2^

^1^University of Lincoln, Lincoln, UK; ^2^International Psychoanalytic University, Berlin, Germany

Traumatic stress is a core concept for psychodynamic theory and practice. It has been thus for more than 100 years. Its continuing relevance arises from a number of considerations, not least of which is emergent views and conceptualizations of repetitions and the various poque-related ways in which they are expressed. This second part of the psychodynamic trauma therapy workshop will concentrate on two ways in which repetitions occur and thus assume a pivotal role in caring trauma survivors: REPETITIONS: First of all, consideration will be given to the phenomenon of repetition from the point of view of what are essential differences between one off occurrences and that which expresses itself in ways that are persistently recurrent. Insights gained from recognition of patterns that are inherent in repetitions will be used to develop a more reflective view of the predicament of trauma survivors, how to improve care and which outcomes are realistic and realisable when some degree of repetition is recognized as integral to the human predicament. With participation from workshop participants and drawing upon their own experiences, the emergent construction of repetition will be applied to persistent intrusive re-experiencing, transference and COUNTERTRANSFERENCE: Reports of traumatic events can cause all kinds of cognitive, emotional, or behavioral reactions. Therapists may have to cope with normative reactions, activation of own behavioral patterns, or experience ego-alien aspects. Determinants of countertransference reactions and possibilities to recognize them will be reflected on. This will be followed by an illustration of specific countertransference topics and strategies of using them as a tool for therapeutic progress. Room will be given for participants to discuss cases of their own clinical work.

## The spectrum of trauma-related disorders
*
Workshop: Simple, complex PTSD and comorbidity, traps in the treatment*

### Simple, complex PTSD, and comorbidity, traps in the treatment 16:45–17:05

#### M. S. Patti, V. Franchi and G. Pistocchi ARP, Milan, Italy

Clinical practice with patients with different trauma-related disorders, occurring singularly or repeatedly in childhood or adulthood, demands unique attention on setting the necessary conditions before treatment. Traumatic events arouse substantial alarm with relevant impairments of impulse control functioning and affect regulation. Especially for patients with complex and repeated trauma experiences occurring during developmental age, these disorders are associated with severe dissociative phenomena that make alliance with therapist very difficult and insecure. Aptitude to use support of other human beings may be compromised; the impairments keep on because of continuous changing of self-conditions, such as anxiety because of expectations of new aggression, tendency to replace traumatic experiences with disruptive behaviors in relation to himself or others, in order to relieve violence, shame, fear, terror, and other overwhelming emotions. Therefore, acquiring reliable diagnostic criteria may be useful in order to decide promptly if focusing intervention on the elaboration of fixated and painful trauma experiences, and to facilitate metabolizing processes, that may provoke worsening, or if taking care of dysfunctional features of personality. Indeed, the suffering of many patients may often get worse, when they deal with their traumatic experiences, inside respect, care, and attention of the therapist.

### The diagnostic process with traumatized patients 17:05–17:25

#### M. S. Patti, G. Pistocchi and V. Franchi ARP, Milan, Italy

The diagnostic process is an important step before starting any treatment. It is a very delicate phase in which we glean precious information on which we subsequently base the therapeutic alliance and treatment plan. This phase may last sometime. When the client presents with posttraumatic symptomatology, the first goal is to understand the impact of the trauma on the client's functioning before the traumatic event and not only on the client's current existence. That is in order to understand more about the dynamics of the client's psychological balance before the trauma and in what way this was impacted on by the trauma. All these help us to appreciate the nature of the symptoms presented. The extent and the strength of the reaction in relation to the seriousness of the traumatic event helps us to perceive whether the construction of our client's personality allows them to go through a process of self-reflection and to integrate aversive experiences into their personality. Sometimes, the traumatic event is not mentioned as the reason for the consultation and only after the initial work of accurate diagnosis (“diagnostic process”) it is possible to recognize a traumatic etiopathogenesis, which will lead us in the setup of the treatment. For the first period, any therapeutical intention aimed at the trauma processing is suspended in order to create a reflective space shared with the patient. The goal of that is to understand how that particular trauma destabilized the patient's way of functioning so far. In particular, the goal is to understand the real stuff of the patient, based on his or her attachment relationship, so that we can set up a treatment plan, tailored to the current difficulties and to the available resources. Contemporary, it is necessary carrying out stabilization of those symptoms (intrusive, hyper-arousal) that are disabling the patient.

### The “impossible” patients: the importance of a multimodal approach 17:25–17:45

#### M. S. Patti, G. Pistocchi and V. Franchi ARP, Milan, Italy

The patients that Chu calls “chronically disempowered” present long-standing difficulties that seem impervious to change. (…) They are highly symptomatic and utilize extensive amount of psychiatric and psychological care. They are prone to severe regression in treatment, which may lead to considerable morbidity even mortality … They also create uncomfortable countertransference responses. With these patients, it is very important to set up a multimodal approach for treatment, tailored on the dissociative parts and their different functioning, and for the collaboration between the different professional figures and services, it is necessary to take care of the patient in a safe situation. It is crucial to keep the patient in a very long phase 1 of treatment (stabilization). First of all, this phase involves the building of a service network (psychiatric and social) that the patient can use, paying attention to the necessary alliance between the different colleagues (this is a fundamental element, based on the seriousness and peculiarity of the pathology). In this network, it is possible to start the work of setting boundaries with precise rules, concerning the way of asking for help (limits on times and duration of calls, limits on sending sms). It is crucial an initial work of psychoeducation/description of the presence of dissociative parts, contemporary to a work of affective regulation that consists of developing self-soothing strategies with a particular attention at creating a trust atmosphere, often subject to ruptures and threats. In fact, the threat of a rupture of the alliance is always present. It is important to activate body safety strategies and emotional modulation before choosing a combination of several approaches such as EMDR, sensorimotor psychotherapy in a context of a constant monitoring of the relationship.

## ORAL, JUNE 7 HALL STUART TUDOR

## Morning Effects of trauma on families and children
*
Symposium: Child sexual abuse and its consequences across Europe*

### Prevalence, incidence and some correlates of sexual abuse of children in Croatia 10:00–10:15

#### M. Ajdukovic, N. Susac and M. Rajter University of Zagreb, Zagreb, Croatia

Sexual abuse of children has been a topic of many international studies, but so far no epidemiological data obtained on a nationally representative sample from Croatia has been published. This study was conducted as a part of the BECAN project and it focused, among other types of violence against children, on lifetime prevalence and one-year incidence of child sexual abuse. The aim of this paper is to examine age and gender differences in prevalence and incidence of child sexual abuse and its correlations with other types of violence in children's lives, as well as some characteristics of children. A probabilistic stratified cluster sample included 2.62% of children aged 11 (*n*=1223), 13 (*n*=1188), and 16 (*n*=1233) from 40 primary and 29 secondary schools. A modified version of ISPCAN child abuse screening tool-children's version was used, with five items referring to child sexual abuse. The instrument used also included socio-demographical questions and questionnaire of peer violence. Results showed that 10.8% of children in Croatia have experienced some form of sexual abuse during their lifetime, while 7.7% of them experienced it in the last year. Prevalence and incidence are higher for older than for younger participants (4.8%, 10.7%, and 16.5% prevalence for particular age groups and 3.7%, 8.1%, and 11.1% incidence). Gender differences were obtained only in the older age groups and only when it came to non-contact sexual abuse, with girls experiencing it more than boys. Children who have experienced sexual abuse have a higher prevalence and incidence of abuse in the family. Those children also experience and perpetrate peer violence more often, skip school more often, have lower grades in school, and use internet communication services more often. These data will be discussed in the context of results obtained in previous studies and the effects of multiple victimization.

### Perpetrators of child sexual abuse in Bosnia and Herzegovina and Croatia: victimization by peers, family members and adult acquaintances 10:15–10:30

#### N. Susac^1^, M. Rajter^1^, J. Brkic Smigoc^2^ and M. Ajdukovic^1^

^1^University of Zagreb, Zagreb, Croatia; ^2^University of Sarajevo, Sarajevo, Bosnia and Herzegovina

This study was conducted as a part of the BECAN project and the focus of this paper will be on presenting data about the perpetrators of sexual abuse against children and comparison of results obtained in Bosnia and Herzegovina and Croatia. The samples in this study are nationally representative and include children aged 11, 13 and, 16 years from Bosnia and Herzegovina (*n=*2664) and in Croatia (*n*=3644). A modified version of ISPCAN child abuse screening tool-children's version was used with five items referring to child sexual abuse by various perpetrators. Children who reported experiencing some form of sexual abuse in the last year or earlier in life were asked to specify whether the perpetrator was an adult man, adult woman, child/adolescent male, or child/adolescent female and then if he/she was a stranger, a person they know, or someone who is related to them. Results showed that prevalence of sexual abuse in Croatia is 4.8%, 10.7%, and 16.5% depending on the age group, while in Bosnia and Herzegovina it amounts to 8.9%, 11.9%, and 26.8%. More detailed item analyses between countries regarding gender and age will be provided. Female participants from Croatia, regardless of their age, most often listed boys who they already knew as perpetrators of both contact and non-contact sexual abuse. Younger male participants most often listed adult men as perpetrators of non-contact sexual abuse but, as their age increased, most frequent perpetrators of sexual abuse became familiar girls. These data will be compared with those obtained in Bosnia and Herzegovina and will be discussed in the context of preventive activities.

### Institutional abuse in Austria: the relation of CSA, different types of sexual violence and psychopathology in adult survivors 10:30–10:45

#### D. Weindl, M. Knefel, V. Kantor, R. Jagsch, A. Butollo and B. Lueger-Schuster Department of Clinical Psychology, University of Vienna, Vienna, Austria

*Background*: Institutional abuse (IA) and its aftermaths are still lacking well-founded research. Nowadays, groups of victims of IA are struggling with different kinds of consequences. Two studies about the psychotraumatological consequences of IA in institutions of the Catholic Church and the country of Lower Austria were made. Data about three dimensions of abuse (emotional, physical, and sexual) were collected and analyzed. Furthermore, psychopathological symptoms were screened with questionnaires. In this paper, we will concentrate on the consequences of CSA in institutional context. *Method*: Four-hundred ninety clearing reports of victims of IA were analyzed, and 224 adult survivors actively participated in these investigations. All sexual violent acts were abstracted into five clusters, which were then related to the psychopathology in adulthood. Questionnaires used: PTSD checklist-civilian version (PCL-C) and the brief symptom inventory (BSI). BSI scales and the PCL-C sum scores of all participants, who especially had experienced CSA were compared with the different clusters of CSA. *Results*: (preliminary): One-hundred thirty-five participants reported any type of sexual violent acts. The existence of those different violent acts showed different outcomes on the BSI scales and the PCL-C sum score. The PCL-C sum score in association with the different types of sexual violence (cluster 1-5) indicate statistical significance, e.g., in cluster 1-(sexual violence with penetration) the PCL-C sum score differentiated significantly between those who had experienced it and those who didn't (cohens *d*=0.50, *p*=0.013, medium effect size). For the BSI-scales, significant differences were identified. *Conclusions*: In these studies, some evidence was found that different types of sexual violence cause different psychopathological impacts on adult survivors. These results might be the first step towards constructing a “trauma-dose”-index. Further, adequate and different interventions according to the experienced type of CSA could be applied in future to the victims.

### Health-related consequences of child sexual abuse 10:45–11:00

#### U. Schnyder^1^, M. A. Landolt^1^, T. Maier^2^ and M. Mohler-Kuo^1^

^1^University of Zurich, Zurich, Switzerland; ^2^Psychiatric Services of the Canton St. Gallen-North, Wil, Switzerland

*Background*: Child sexual abuse (CSA) is a worldwide problem due to its high prevalence and short-term and long-term consequences. The present study examined health-related quality of life (HQoL) and survivors’ behavioral and emotional problems by type of CSA. *Method*: The present study on adolescent victimization involved a nationally-representative sample of 9^th^ grade students 13–20 (15.5±0.66) years old in Switzerland. Data were collected through self-reported computer-assisted questionnaires between September 2009 and May 2010. Fifteen forms of sexual victimization were assessed using a newly-developed Child Sexual Abuse Questionnaire (CSAQ). The sample consisted of 6'787 students. CSA was further categorized as ‘non-contact CSA only’ and ‘CSA with physical contact’. Health-related consequences were assessed using the SF-12 and the Strength and Difficulty Questionnaire (SDQ). *Results*: About 24% of girls and 12% of boys reported having experienced ‘non-contact CSA only’, and 15% of girls and 5% of boys reported about ‘CSA with physical contact’. Children who reported ‘CSA with physical contact’ had lowest HQoL in both mental (mean=41.7 [40.9–42.6]) and physical health (mean=52.3 [51.6–53.0] compared to children with ‘non-contact CSA only’ (mean=44.9 [44.3–45.5] and 53.8 [53.3–54.2] respectively) and ‘no history of CSA’ (mean=48.9 [48.6–49.2] and 54.5 [54.1–54.8] respectively). Similarly, children who had experienced ‘CSA with physical contact’ reported more behavioral and emotional problems (mean=13.2 [12.8–13.7]) than those with ‘non-contact CSA only’ (mean=11.9 [11.6–12.2] and ‘no history of CSA’. Similar pattern was found in both girls and boys. Results remained the same for all three outcomes after controlling for gender, not living with biological parents, and nationality in multiple regression models. *Conclusion*: Children who experienced CSA reported lower quality of life and more behavioral and emotional problems. A gradient effect was observed by the severity of CSA. Children who experienced CSA with physical contact had worst health status. Immediate intervention for victims of CSA is necessary to reduce long-term consequences.

## Miscellaneous
*Symposium: From vision to daily routine - practical clinical implementation of interdisciplinary teamwork*

### A specific interdisciplinary outpatient pre-program as an example of seamless transition from outpatient to inpatient setting 11:45–12:00

#### J. Binder Integrierte Psychiatrie Winterthur, Winterthur, Switzerland

Specialized wards for posttraumatic stress disorders (PTSD) often have a long waiting-list and generate longer treatment times. The latter frequently causes problems with health insurance providers or other cost-bearers. In order to constructively address these difficulties and to take into account both the needs of the patient and cost limitations, an outpatient pre-program in a small-group setting was included in the treatment concept of IPW's (Integrierte Psychiatrie Winterthur) specialized ward for PTSD. This 8-week pre-program prior to planned inpatient admission not only utilizes patients' waiting-list time for therapeutic interventions, but also reduces the average length of inpatient treatment. Goal of this symposium is to present the elements of the program (psycho-education, trauma-adapted skills-training, resource-oriented movement therapy) and to report on our experiences in working with this comprehensive inpatient-outpatient and cross-disciplinary intervention. Benefits such as solid psycho-educational knowledge, early trust-building and improved predictability of patients' readiness for trauma therapy considerably facilitate the earlier introduction of exposure-based therapy in the inpatient setting in comparison with patients who did not participate in the pre-program, thus reducing the length of hospitalization.

### Multimethod teamwork in trauma-specific psychotherapy 12:00–12:15

#### S. Weber Integrierte Psychiatrie Winterthur, Winterthur, Switzerland

Patients who are admitted to our specialized ward for post-traumatic stress disorders are allocated to a one-to-one psychotherapist based on their case history and clinical characteristics. We offer a range of trauma-specific interventions, although not every therapist uses every intervention. The specialization and continuing education of the individual therapist, therefore, plays an important role in the allocation of patients, as the aim is to match trauma-specific interventions to individual disorder characteristics and predominant symptoms. In practice, it often becomes necessary to adapt or extend psychotherapeutic case planning at short notice in the course of treatment. In order to flexibly achieve these process changes, we selectively work with multimethod interventions in individual cases by using other therapists within the team. This occurs during long periods of absence cover, but also selectively for one-to-one sessions. With the help of short case studies, we will demonstrate in which specific situations we decided on a multimethod therapy and consequences for the course of treatment.

### Courage for new beginnings 12:15–12:30

#### K. Wild Integrierte Psychiatrei Winterthur, Winterthur, Switzerland

This is a case study of the course of treatment of a 60-year-old Chilean, who fled to Switzerland 40 years ago after incarceration and torture. He lived for his family, work, and football and had his traumatic memories much under control until he was involved in a road traffic accident 9 years ago, in which he was trapped in his car and had to be cut free. His physical injuries were slight. Nevertheless, from that moment on he suffered from extreme pain. His legs refused to carry him. He described his own catastrophic condition figuratively as “I lived like a house plant”. Our primary therapeutic approach was on a physical level, as taking the case history and beginning with NET had caused emotional flooding and denial of any connection with events in Chile. Intensive one-to-one movement therapy was enhanced by psycho-educational psychiatric sessions, one-to-one nursing with the main focus on coping strategies in everyday life as well as physiotherapy. The movement therapy approach focused on the patient's feeling of security, which he initially only achieved when lying in the embryo position. This symposium highlights how minimal changes in body position and the tiniest movements gradually increased the patient's feeling of security, until he managed to stand up without using a stick or props whilst at the same time being fully aware of his actions. In the course of treatment, he gained understanding of how his emotions influenced his physical condition and insight into personal means of control. Connections to early traumata could be introduced gradually.

### Integration of interdisciplinary teamwork in everyday clinical practice through trauma-related topics 12:30–12:45

#### M. Stadtmann Integrierte Psychiatrie Winterthur, Winterthur, Switzerland

The IPW's (Integrierte Psychiatrie Winterthur) specialized ward for posttraumatic stress disorders works with an interdisciplinary concept aimed at improving symptom management in an inpatient setting. Tasks and treatment areas are defined and allocated according to the correspondent expertise of individual members of the treatment team. The goal is to offer a holistic and complementary form of therapy, which empowers patients to improve symptom management and therefore facilitate coping in their daily lives. The integration of this interdisciplinary concept in everyday clinical practice is achieved through so-called “weekly themes”. Important trauma-related topics are focused in turn for a two-week period. Topics comprise security/insecurity, self-efficacy, anger/aggression, avoidance, closeness/distance, future perspectives, and self-acceptance and are addressed verbally and non-verbally. The movement therapy group enables improved body perception based on the specific theme. In the art therapy, group patients address the topic with composition and expression. The psycho-education group focuses on the verbal-cognitive level. The nursing staff introduces the theme with the help of theoretical principles and is responsible for transfer to everyday life, using practical situations as examples. The above groups are distributed over the weekly therapy plan. In addition, the evaluated results will be presented.

## Afternoon The spectrum of trauma-related disorders
*
Symposium: Assessment of childhook trauma exposure and adult posttraumatic symptoms in routine clinical work*

### Stability of childhood trauma questionnaire-scores before and after therapy 15:15–15:35

#### K. Arefjord^1^, D. Winje^1^, A. Dovran^2^, L. Waage^3^ and A. L. Hansen^4^

^1^Department of Clinical Psychology, University of Bergen, Bergen, Norway; ^2^Department of Clinical Psychology, University of Bergen, Bergen, Norway; District Psychiatric Center Kronstad, Haukeland University Hospital, Bergen, Norway; ^3^Centre for Research and Education in Forensic Psychiatry, Haukeland University Hospital, Bergen, Norway; Correctional Service, Ontario, Canada; ^4^Faculty of Psychology, Department of Psychosocial Science, University of Bergen, Bergen, Norway

The childhood trauma questionnaire-short form (CTQ-SF) is a 28-item retrospective measure of the frequency and severity of different types of abuse and neglect. CTQ-SF has exhibited good test-retest reliability and good convergent validity with measures of PTSD, dissociation and depression, and discriminant validity with measures of vocabulary and social desirability. In this study, the CTQ-SF was administered at pre- and post-treatment to a sample of patients in therapy with psychology students in supervised clinical training at an out-patient clinic. The stability of scores on the CTQ-SF was examined. Preliminary analyses indicate that CTQ-SF demonstrate good test-retest reliability after ended therapy. The stability of the CTQ-SF in the context of reduction of in different types of psychopathology in the sample, contributes to evidence supporting the accuracy of retrospective self-reports of childhood abuse.

### Multiple types of childhood trauma in a sample of sexually abused adults 15:35–15:55

#### I. Steine^1^, D. Winje^2^, A. Dovran^3^ and S. Pallesen^4^

^1^Faculty of Psychology, Department of Biological and Medical Psychology, University of Bergen, Bergen, Norway; Child and Adolescent Psychiatric Outpatient Clinic, Fana, Fusa, Austevoll, Haukeland University Hospital, Bergen, Norway; ^2^Faculty of Psychology, Department of Clinical Psychology, University of Bergen, Bergen, Norway; ^3^Department of Clinical Psychology, University of Bergen, Bergen, Norway; District Psychiatric Center Kronstad, Haukeland University Hospital, Bergen, Norway; ^4^Department of Psychosocial Science, University of Bergen, Bergen, Norway

*Background*: Sexual abuse is a widespread problem in the general population in Norway as well as internationally. A recent WHO-study showed that childhood adversities, such as sexual, physical, and emotional abuse are highly interrelated. The childhood trauma questionnaire short form (CTQ-SF) is a well-validated screening instrument of childhood sexual, physical, and emotional abuse, as well as of physical and emotional neglect. A recent review of studies utilizing the CTQ-SF reported a high prevalence of severe emotional abuse and neglect in both clinical and victim populations. Taken together, the literature underline the importance of assessing multiple rather than single types of childhood adversities in studies of child abuse and neglect, in order to better contain the complex and inter-related nature of the topic. *Objective*: The aim of the present study was, therefore, to investigate the prevalence of emotional and physical abuse and neglect in a sample of sexual abuse survivors in Norway, using the CTQ-SF in order to ensure compatibility with previous studies. *Method*: Throughout 2011 and 2012, approximately 300 users of support centres for sexual abuse survivors in Norway completed a comprehensive questionnaire, including among other things the 28-item version of the CTQ-SF. *Results*: Preliminary results will be presented and compared to existing literature. Implications of the findings will be discussed.

### Childhood trauma, attachment style, psychopathy and underlying biological mechanisms 15:55–16:15

#### L. Waage^1^, A. L. Hansen^2^, D. Winje^3^, A. Dovran^4^ and K. Arefjord^3^

^1^Correctional Service, Ontario, Canada; Centre for Research and Education in Forensic Psychiatry, Haukeland University Hospital, Bergen, Norway; ^2^Faculty of Psychology, Department of Psychosocial Science, University of Bergen, Bergen, Norway; Centre for Research and Education in Forensic Psychiatry, Haukeland University Hospital, Bergen, Norway; ^3^Department of Clinical Psychology, University of Bergen, Bergen, Norway; ^4^Department of Clinical Psychology, University of Bergen, Bergen, Norway; District Psychiatric Center Kronstad, Haukeland University Hospital, Bergen, Norway

*Objective*: The aim of this study was to investigate the relationship between childhood trauma, attachment styles, facets of self-reported psychopathy, and underlying biological markers. *Method*: One-hundred four inmates from Bergen prison participated in this study. The childhood trauma questionnaire-short form (CTQ-SF) was used to assess childhood maltreatment. Attachment style was measured by the experiences in close relationships (ECR) and self-reported psychopathy was measured by self-report of psychopathy-III (SRP-III). Underlying biological mechanisms were measured as heart rate variability and heart rate using the Actiheart system. *Outcomes*: When looking at the four categories of adult attachment styles (secure, dismissing, preoccupied, and fearful) the results indicated that individuals with fearful attachment style reported high levels of childhood maltreatment and posttraumatic stress symptoms. Moreover, the results revealed that participants with fearful attachment style had higher parasympathetic activity compared to secure attachment style. However, there was an abnormal relationship between sympathetic and parasympathetic activity in the group of fearful attached participants. We also examined the relationship between the two-dimension model of attachment (avoidant and anxious) and self-reported psychopathy (SRP-III), and found that the avoidant dimension was positive related to all facets of psychopathy. The strongest relation was with the callous facet. *Conclusion*: The present results indicated that there might be a relationship between childhood trauma, attachment, and self-reported psychopathy. Specific underlying biological mechanisms that might be affected due to adverse childhood experiences were identified.

## The spectrum of trauma-related disorders
*Symposium: Psychological processes following childhood trauma, separations and loss–more than PTSD?*

### Autobiographical memory specificity in complex PTSD and DID 16:45–17:05

#### R. Huntjens^1^, A. Van Minnen^2^, D. Hermans^3^ and I. Wessel^1^

^1^University of Groningen, Groningen, The Netherlands; ^2^Overwaal Center for Anxiety Disorders, Radboud University Nijmegen, Nijmegen, The Netherlands; ^3^University of Leuven, Leuven, Belgium

This study investigated autobiographical memory in patients with complex PTSD and dissociative identity disorder (DID). When recalling autobiographical events, many emotionally disturbed patients summarize categories of events rather than retrieving a single episode, so-called over general memory. One of the key mechanisms considered underlying over general memory retrieval is affect regulation. The recollection of general memories may produce less affect than the recollection of specific episodic memories, thus enabling the individual to carry on with normal daily life. The current study was aimed at investigating over general memory in a sample of patients with experiences of chronic sexual and physical abuse in childhood. We included the autobiographical memory test (AMT), in which respondents have to provide autobiographical memories in response to positive and negative cue words. In addition, we also asked participants to indicate the perspective of remembering for each retrieved event. That is, a distinction can be drawn between two different perspectives of remembering. Field memories refer to memories in which the person remembers the event from the original viewpoint (i.e., seeing it again through their own eyes). Observer memories refer to memories in which the person sees him or herself while remembering the event (i.e., from the perspective of a detached spectator). The results indicated that the complex PTSD and DID patients recalled significantly fewer specific memories compared to controls. Associations with several types of posttraumatic symptoms will be presented, including cognitive and behavioral avoidance.

### Effects of separation and loss on PTSD symptom change over time in a treatment seeking sample 17:05–17:25

#### G. Smid and N. Van Der Aa Foundation Centrum '45/Arq Research Program, Oegstgeest, The Netherlands

*Background*: Separation and loss experiences often accompany traumatic events, especially in refugees. Current conceptualizations of loss-related psychopathology emphasize similarities with posttraumatic stress disorder (PTSD) (Maercker & Znoj, 2010). It is unclear what the effects of separation and loss experiences are on PTSD levels as well as change in these levels during treatment. *Methods*: In a sample of treatment seeking individuals at a specialized trauma treatment centre (N=139), we assessed PTSD symptoms as well as exposure to traumatic events using the Harvard trauma questionnaire at the start of treatment (care as usual) and one year later. Fifty-eight percent of the sample consisted of refugees, whereas the other part consisted of non-refugee groups, such as military veterans, police officers, and other violence victims. We used latent growth modeling to evaluate the effects of separation and loss experiences as well as being refugee on both baseline level of PTSD symptoms and change during treatment. *Results*: More separation and loss experiences were associated with a higher baseline PTSD symptom level (standardized regression weight=0.45, p<0.001). In addition, more separation and loss experiences were associated with a greater reduction in PTSD symptoms after one year of treatment. Refugees reported significantly more frequent exposure to almost all (21 out of 24) types of traumatic event, including separation and loss experiences. Nonetheless, after adjusting for separation and loss experiences, the baseline level of PTSD symptoms in refugees was only marginally elevated compared with non-refugee groups (standardized regression weight=0.17, *p*=0.08). *Conclusion*: Separation and loss experiences strongly contribute to the distress associated with PTSD. Results suggest that the effects of these experiences can be effectively targeted during treatment.

Reference

Maercker, A., & Znoj, H. (2010). The younger sibling of PTSD: Similarities and differences between complicated grief and posttraumatic stress disorder. *European Journal of Psychotraumatology, 1*, 5558, doi: http://dx.doi.org/10.3402/ejpt.v1i0.5558

### Mental health problems associated with female genital mutilation 17:25–17:45

#### J. Knipscheer^1^, E. Vloeberghs^2^, A. Van Der Kwaak^3^, Z. Naleie^4^ and M. Van Den Muijsenbergh^5^

^1^Arq Psychotrauma Expert Group, Diemen/Department of Clinical and Health Psychology Utrecht University, Utrecht, The Netherlands; ^2^Pharos, knowledge-and advisory center for migrants, refugees and health, Utrecht, The Netherlands; ^3^Royal Institute for the Tropics/University of Amsterdam, Amsterdam, The Netherlands; ^4^Federation of Somali Associations, Rotterdam, The Netherlands; ^5^Radboud University Medical Centre Nijmegen, Nijmegen, The Netherlands

*Objective*: Although experts have assumed that circumcised women are more prone to developing mental health problems than the general population, there has been little research to confirm this claim. This study investigated the mental health status of adult women who had undergone genital mutilation in their youth in Africa and later in life migrated to Europe. Risk factors associated with the report of mental health problems were also determined. *Method*: Sixty-six circumcised women originating from five African countries (Somalia, Ethiopia, Sudan, Eritrea, and Sierra Leone) and who had migrated to The Netherlands were assessed by means of four standardized questionnaires (HTQ-30, HSCL-25, COPE-Easy, LAS) and topic interviews. *Results*: One-third of the respondents met criteria for affective or anxiety disorders, scores indicative for PTSD were presented by 17.5% of the subjects. Infibulation as the type of circumcision, a lively memory of the circumcision, an avoidant coping style (in particular substance abuse), and lack of income were significant factors associated with psychopathology. *Conclusions*: There is no reason to pathologize the consequences of female genital mutilation, but specific attention to the serious psychosocial problems among a considerable minority group (especially infibulated women who remember their circumcision well and use avoidant ways of coping) is warranted.

## ORAL, JUNE 7 HALL SYDNEY

## Morning
*Open Papers: Occupational health and secondary trauma I*

### Physicians and nurses involved in adverse patient events are traumatized and have a higher risk in burnout: a nationwide multicenter study 10:00–10:15

#### E. Van Gerven^1^, S. Vandenbroek^2^, L. Godderis^3^, H. De Witte^4^, M. Euwema^4^, W. Sermeus^1^ and K. Vanhaecht^1^

^1^Department of Public Health, University of Leuven, Leuven, Belgium; ^2^IDEWE, Leuven, Belgium; ^3^Department of Occupational, Environmental and Insurance Medicine, University of Leuven, Leuven, Belgium; ^4^Department of Psychology, University of Leuven, Leuven, Belgium

In healthcare, one out of seven patients is involved in an adverse event. Adverse events can lead to two types of victims. The first and most important victim is the patient and family. The second victim is the involved physician and/or nurse. The second victim is defined as a health care provider involved in an unanticipated adverse patient event or medical error, who becomes victimized in the sense that the provider is traumatized by the event. They feel personally responsible, feel as they have failed their patient and second-guess their clinical skills and knowledge base. Symptoms are both personal and professional and include posttraumatic stress disorder (PTSD). PTSD has a strong association with the existence of burnout. We performed a nationwide cross-sectional multicenter study in 37 hospitals in Belgium. One-thousand one ninety-eight physicians and 4,635 nurses participated in the web survey regarding the prevalence of burnout and adverse events. Results show that involvement in an adverse patient event implies a 2-fold increased risk of burnout. There is a high correlation between adverse events and depersonalization. Since involvement in an adverse patient event has serious consequences on emotional and professional level, support systems need to be in place to protect both patients and health care workers. Several European institutes and universities are actually repeating this study and are launching a new European research network to collaborate, share knowledge, and launch scientific studies on this health policy topic.

### Perception of Threat and Safety at Work among Government Employees after the 2011 Oslo Terrorist Attack 10:15–10:30

#### A. Nissen and T. Heir Norwegian Center for Violence and Traumatic Stress Studies

The aim of this study was to examine the perception of threat and safety at work among employees who have experienced a terrorist attack directed at their workplace. Employees in 14 of the 17 Norwegian ministries were asked about threat and safety perception at work, traumatic exposure, and symptoms of posttraumatic stress disorder (PTSD) 9 to 10 months after the terrorist car bomb attack in Oslo on the 22nd of July, 2011. Of the 1881 ministerial employees who completed the survey, 198 (10.5%) were at work in the government district when the terrorist bomb exploded. This high stress-exposed group reported a significantly higher level of perceived threat (odds ratio [OR]=3.03), and a lower level of perceived safety (OR=2.81) at work compared to low stress-exposed employees. When controlling for PTSD symptoms, however, the ORs did not significantly differ between the two groups, whereas PTSD symptomatology in itself was significantly associated with both high perceived threat (OR=2.87) and low perceived safety (OR=2.59). Women (OR=0.49), older employees (OR=0.78) and highly educated employees (OR=0.43) had significantly lower levels of perceived threat after controlling for PTSD symptoms. Our data suggest that employees with a high degree of stress-exposure during a workplace terrorist attack have greater fears of future attacks and feel less safe at work after the attack compared to low stress-exposed employees. It appears that this can be explained by the higher prevalence of PTSD symptoms among high stress-exposed employees.

### Integrated group counseling for mental health & resilience of Thai army rangers in southern most provinces of Thailand 10:30–10:45

#### D. Chongruksa^1^, P. Prinyapol^1^, S. Sawatsri^2^ and C. Pansomboon^3^

^1^Prince of Songkla University, Songkhla, Southern Thailand; ^2^Pramongkok Hospital, Bangkok, Thailand; ^3^Youth Observation, Tumbon Bangjak Muang District, Thailand

This research presented an integrated group counseling developed for Thai army rangers deploying in the three southern most provinces during the unrest. The intervention focused on the improvement of mental health and resilience, and the reduction of risk symptoms related to stress. The group process was the interactive model of existential therapy, art therapy using mandala, Cognitive Behavioral Therapy and Psycho-education. The design was the control experiment. Forty-four voluntary rangers aged between 22 and 45 years were randomly assigned equally to the experiment and the control groups. They were selected from 384 rangers derived by cluster sampling based on low scores of resilience inventory and Thai mental health inventory (TMHI-54), and on high scores of Thai general health questionnaires (GHQ 28). The assessment was done 3 times: before treatment, at termination, and at 1-month follow up. The experiment attended 20 session group counseling while the control received educational information. The data were analyzed by two-way MANOVA and ANOVA: repeated measure. The results were: 1. The average scores of those attending group counseling after the experiment and follow up were significantly different from those of the control in all three inventories with relative medium effect sizes and at 1-month follow up with small decrease in effect sizes. The significant differences were also revealed in all subscales: hardiness, optimism, resources and purposes for resilience inventory; symptom, anxiety, social dysfunction, and depression for GHQ 28; and mental state, mental capacity, mental quality, and social support for TMHI-54. 2. Of all 384 rangers, half of them scored at low level on resilience and nearly half on mental health. About two-thirds scored at normal level on general health where more than half were at high level on depression subscale.

### Indirect exposure to client trauma and the impact on trainee clinical psychologists: secondary traumatic stress or vicarious traumatization? 10:45–11:00

#### G. Turpin^1^, R. Makadia^1^ and R. Sabion-Farrel^2^

^1^Department of Psychology, University of Sheffield, Sheffield, UK; ^3^University of Nottingham, Nottingham, UK

This study investigated the extent of exposure to trauma work among trainee clinical psychologists and its impact on well-being. It investigated which theoretical model (secondary traumatic stress (STS), vicarious traumatization (VT), or even a non-specific model of general psychological distress) could best account for any negative effects associated with indirect exposure to client trauma. Five-hundred sixty-four trainees participated in an online survey, which included self-report measures of general psychological distress, trauma symptoms, and disrupted beliefs. Most trainees had caseloads of 1-2 trauma cases, with the most common trauma experienced by their clients as being sexual abuse. Exposure to trauma work was not related to self-reported general psychological distress or disrupted beliefs within trainees, but was a significant predictor of trauma symptoms. Level of stress of clinical work and quality of trauma training contributed to the variance in trauma symptoms. It is concluded that the study provides support for STS but lacked evidence to support VT or a non-specific model of general psychological distress. The implications for training clinical psychologists and other psychological therapists are discussed.

### Trauma for physicians and nurses after an adverse patient event: a systematic literature research of the impact on functioning and well-being 11:00–11:15

#### E. Van Gerven^1^, D. Seys^1^, S. Scott^2^, J. Conway^3^, A. Wu^4^, M. Panella^5^, M. Euwema^6^, W. Sermeus^1^ and K. Vanhaecht^1^

^1^Department of Public Health, University of Leuven, Leuven, Belgium; ^2^Sinclair School of Nursing, University of Missouri, Missouri, USA; ^3^Institute for Healthcare Improvement, Cambridge, USA; ^4^Johns Hopkins Bloomberg School of Public Health, Baltimore, USA; ^5^Faculty of Medicine, Amedeo Avogadro University of Eastern Piedmont, Novara, Italy; ^6^Department of Psychology, University of Leuven, Leuven, Belgium

One out of seven patients is involved in an adverse event. Adverse events within healthcare settings can lead to two victims: the first and most important victim is the patient and family. The second victim, however, is the involved health care professional. The objectives of this systematic literature research were to determine definitions of the second victim concept, the prevalence and the impact of the adverse event on the second victim, and the used coping strategies. A second victim is defined as a health care provider involved in an unanticipated adverse patient event and/or medical error who become victimized in the sense that the provider is traumatized by the event. Frequently, second victims feel personally responsible for the unexpected patient outcomes and feel as though they have failed their patient, second-guessing their clinical skills and knowledge base. It is estimated that almost 50% of all health care providers become a second victim once in their career. Feelings of guilt, anger, frustration, psychological distress, fear, and PTSD are the most common psychosocial and physical symptoms. The error can have an impact on both the personal and professional life of the second victim. The coping strategies used by second victims have an impact on their patients, colleagues, and themselves. Defensive as well as constructive coping strategies have been reported in practice. Support networks need to be in place to protect both the patient and involved health care providers.

Reference

Seys, D., Wu, A. W., Van Gerven, E., Vleugels, A., Euwema, M., Panella, M., et al. (2012). Health care professionals as second victims after adverse events: A systematic review. *Evaluation & the Health Professions*. In press. doi: 10.1177/0163278712458918.

## Open Papers: Occupational health and secondary trauma II

### The relationship between secondary traumatic stress and job burnout: a meta-analysis 11:45–12:00

#### R. Cieslak^1^, K. Shoji^2^, A. Douglas^3^, E. Melville^3^, A. Luszczynska^4^ and C. Benight^5^

^1^Department of Psychology, University of Social Sciences and Humanities, Warsaw, Poland and Trauma, Health, and Hazards Center, University of Colorado, Colorado Springs, USA; ^2^Trauma, Health, and Hazards Center, University of Colorado, Colorado Springs, USA; ^3^Department of Psychology, University of Colorado, Colorado Springs, USA; ^4^University of Social Sciences and Humanities, Wroclaw, Poland and Trauma, Health, and Hazards Center, University of Colorado, Colorado Springs, USA; ^5^Trauma, Health, and Hazards Center and Department of Psychology, University of Colorado, Colorado Springs, USA

This study is aimed at reviewing the evidence for relationships between secondary traumatic stress (STS) and job burnout among professionals working with trauma survivors. Critical moderators explored were: 1) the type of measurement, 2) the conceptualization of STS and job burnout, 3) gender, and 4) the cultural context. To evaluate cultural context we focused on differences between the findings obtained in the US and other countries, as well as the differences in findings obtained for English-language measures *versus* other-language measures. A systematic review of literature yielded 41 original studies, analyzing data from a total of 8,256 workers. Meta-analysis indicated that associations between STS and job burnout were strong (weighted *r*=.69). Studies applying measures developed within the compassion fatigue framework (one of the conceptualizations of job burnout and STS) showed significantly stronger relationships between STS and job burnout, indicating a substantial overlap between measures (weighted *r*=.74; 55% of shared variance). Research applying other frameworks and measures of job burnout (i.e., stressing the role of emotional exhaustion) and STS (i.e., focusing on symptoms resembling posttraumatic stress disorder or a cognitive shift specific for vicarious trauma) showed weaker, although still substantial associations (weighted *r*=.58; 34% of shared variance). Significantly stronger associations between job burnout and STS were found for: 1) studies conducted in the US compared to other countries, 2) studies using English-language versions of the questionnaires compared to other-language versions, and 3) in predominantly female samples. In conclusion, results suggests that due to high correlations between STS and job burnout, there is a substantial likelihood that a professional exposed to secondary trauma would report similar levels of job burnout and STS, particularly if job burnout and STS were measured within the framework of compassion fatigue.

### Occupational stress: approach and intervention with paid-professional firefighters 12:00–12:15

#### A. Sommerfeld and S. Wagner University of Northern British Columbia, Prince George, Canada

The primary purpose of this project was to answer the question “What support do firefighters and their partners feel they need for the prevention and treatment of fire rescue occupational stress”? phase 1 of the research was a reflexive ethnography of facilitator experiences during presentation of a workshop to paid-professional fire rescue members based on the Veteran Affair's Wellness Kit. Phase 2 consisted of completion, coding and, analysis of in-depth personal interviews with select fire rescue members and their partners. Results from phase 1 indicated that the workshop format was positively accepted and well attended; members stated that they learned from the workshops and would recommend other similar learning opportunities. Results from the phase 2 interviews revealed findings of positives from the job such as time-off, but less positives for safety, stress, technology usage, and training. Training was suggested for improvement in several areas, and continuing discussion of behavioral health was an often requested topic. Overall, the present research supported a broad holistic view of stress that institutes overall cultural and organizational changes to support stress prevention. Despite this ongoing need for additional behavioral health management information, firefighters and their partners believed the level of support within the fire department could be sufficient for the prevention and treatment of occupational stress. Further, acknowledgement of existing psychological issues remains paramount to prevention strategies for this group.

### Hostility in firefighters: personality and mental health 12:15–12:30

#### S. Wagner, R. Pasca and J. Crosina University of Northern British Columbia, Prince George, Canada

*Purpose*: To evaluate the self-reported personality and mental health symptomatology of a group of firefighters characterized as high-hostile *versus* those characterized as low-hostile. *Methodology*: A group of paid-professional firefighters (*n*=94) completed a questionnaire study that included use of a demographic questionnaire, the impact of event scale-revised (IES-R), the NEO personality inventory (NEO-PI), Framingham type A scale, and the symptom checklist-90 (SCL-90). *Analysis*: Firefighters were divided into high-hostile and low-hostile using the 50th percentile as the categorizing factor. Once categorized, group differences were investigated regarding personality characteristics as measured by the NEO-PI, controlling for type A behavior. Group differences were also investigated for mental health as measured by the SCL-90, controlling for both type A behavior and neuroticism. *Findings*: Low-hostile firefighters self-reported greater agreeableness; whereas, high-hostile firefighters self-reported greater neuroticism. Further, high-hostile firefighters self-reported greater posttraumatic symptomatology, as well as increased symptoms of somatization, obsessive-compulsive, interpersonal sensitivity, depression, anxiety, phobia anxiety, paranoid ideation, and psychoticism. *Originality/value*: To our knowledge, this is the first study to specifically investigate the impact of hostility on mental health of paid-professional firefighters. In addition, the findings suggest that interventions to screen for and subsequently, reduce hostility in firefighters may be beneficial for mental health in this occupational group.

### Gender differences in humanitarian aid workers 12:30–12:45

#### H. Siller^1^ and B. Juen^2^

^1^Women's Health Centre of Innsbruck Medical University Hospital, Medical University Innsbruck, Innsbruck, Austria; ^2^Department of Psychology, University of Innsbruck, Innsbruck, Austria

Gender differences in trauma (e.g., trauma type, lifetime prevalence of trauma) and the posttraumatic stress disorder are well-established and may point to a different vulnerability of women to traumatic events. Studies on short- term delegates after the tsunami have drawn a more complex picture, indicating that the social reference of men and women should be taken into account. The study was set on humanitarian aid workers using quantitative and qualitative approaches to assess resources, stress, gender - and culture - specific aspects in humanitarian aid. Analysis was done on 22 semi-structured interviews with humanitarian aid workers using the content analysis. Gender-specific content was additionally analyzed using an intersectional approach to refine results. The poster will focus on qualitative results. Results show that resources during the mission refer to effectiveness of work, support and safety provided by the organization, team-related resources and culturally adapted coping strategies. Stressful factors during the mission were more strongly emphasized for external factors (work- and organization-related) and team-related factors. Moreover, stress was expressed after returning from the international work, leading to a specific feeling of detachment and difficult re-entry. Returning to the home country was also related to a changed appreciation of the home country. The results of the intersectional approach on gender revealed feelings of in/equality of men and women, impact of gender mixture in teams, and also the importance of the status of a person. Results can be translated for further implications for training and further support of humanitarian aid workers.

### Examining disaster mental health workforce capacity 12:45–13:00

#### L. Reifels^1^, L. Naccarella^2^, G. Blashki^3^ and J. Pirkis^1^

^1^Centre for Health Policy, Programs & Economics, Melbourne School of Population Health, The University of Melbourne, Melbourne, Australia; ^2^Australian Health Workforce Institute, The University of Melbourne, Melbourne, Australia; ^3^Nossal Institute for Global Health & Melbourne Sustainable Society Institute, The University of Melbourne, Melbourne Australia

*Objective*: Despite considerable advances in disaster mental health a lack of systematic data on the capacity of the multifaceted workforce which provides best-practice mental health support to disaster-affected individuals constitutes one of the biggest challenges to effective disaster response planning. In order to address this challenge and inform future disaster planning, we conducted a state-level examination of the profile and capacity of the disaster mental health workforce in Victoria, Australia. *Method*: Comprehensive workforce scoping (including professional and paraprofessional providers) informed recruitment for a cross-sectional online survey (*n*=791). The survey elicited information regarding the workforce profile, key indicators, and correlates of disaster mental health capacity, as well as key barriers and enablers of effective disaster response participation. Data analysis involved a combination of descriptive, correlational, and thematic analyses of survey data. *Results*: Study findings highlight the diverse profile and considerable variability in the disaster mental health capacity of providers. Existing workforce strengths include high provider interest and mobility levels, and a good understanding of disaster impacts. However, many providers lacked disaster work experience and confidence to provide best-practice interventions, with confidence levels corresponding to training and provider experience. *Conclusions:* Study findings provide a broad-based training mandate, whilst highlighting the need for practice opportunities and structural provider support. Cross-professional capacity surveys focused on best-practice disaster mental health interventions can provide systematic data to inform strategic disaster workforce planning, sustainable capacity building and provision of enhanced support services in disaster-affected communities.

## Afternoon
*Open Papers: Occupational health and secondary trauma III*

### Traumatic stress in intensive care staff: associations with burnout and coping 15:15–15:30

#### C. Dalia^1^, G. Colville^2^, J. Brierley^3^, K. Abbas^3^ and L. Perkins-Porras^1^

^1^St George's University of London Medical School, London, UK; ^2^St George's Hospital, London, UK; ^3^Great Ormond Street Hospital for Children, London, UK

*Background*: A recent meta-analysis has found that healthcare workers are at risk of posttraumatic stress symptoms arising from work-related critical incidents. It is hypothesized that repeated exposure to traumatic medical events in Intensive Care settings places this group of health professionals at increased risk of developing symptoms of posttraumatic stress disorder (PTSD). *Objectives*: 1) To ascertain level of posttraumatic stress symptomatology in a mixed staff group working in an intensive care setting. 2) To examine associations between PTSD symptoms, burnout and coping strategies. 3) To determine whether the use of particular coping strategies was associated with scores on a PTSD screening instrument. *Design*: Cross-sectional questionnaire study *Participants*: Fifty-eight health professionals working on a pediatric/neonatal intensive care unit. *Measures*: Trauma screening questionnaire (TSQ); abbreviated maslach burnout inventory (aMBI), list of coping strategies. *Results*: In total 48 (83%) participants reported at least one posttraumatic stress symptom in the previous week and a significant number, *n*=10 (17%), scored above the clinical cut-off on the TSQ, suggesting they were at risk of developing PTSD in relation to traumatic work-related experiences. There was no significant association with gender, number of years qualified, whether the staff member lived alone or had children, or whether they were a doctor or a nurse. Scores on the TSQ were however associated with scores for emotional exhaustion (*r=*0.496, *p*<0.001) and depersonalization (*r=*0.273, *p*=0.038) on the aMBI. People with higher TSQ scores were more likely to report ignoring stress (*p*=0.008) and taking time off (*p=*0.039) as coping strategies and less likely to say they had hobbies (*p=*0.02). *Conclusion*: A significant minority of intensive care staff reported PTSD symptoms relating to their work. Symptoms were independent of demographic factors or length of experience but were related to burnout. More research is needed on the prevalence of psychological distress in this group.

### “An investigation into the consequences and effects of secondary trauma on health professionals working with traumatized individuals” 15:30–15:45

#### R. Konistan London Metropolitan University, London, UK

Secondary traumatization has been reported among professionals working with traumatized individuals, hence themselves fall victim to secondary traumatic stress (Figley, 2002a) That is through exposure and while engaging in helping or wanting to help a traumatized individuals. Secondary trauma is receiving attention in recent years particularly within mental health professions (Bride, 2002). Sabo (2006) found that nurses who provide intensive take to patients fall victims and suffer compassion fatigue. Thomas (2004) suggested that almost seven percent of professionals who work with victims of trauma display emotional reactions that are very similar to symptoms of PTSD. The American Psychological Association has indicated that these symptoms can be grouped under three categories, which are re-experiencing the traumatic event, augmented arousal and relentless avoidance and, numbing of widespread thoughts associated with the trauma. Thomas (2002) approved that STS (i.e., stress reaction almost indistinguishable to PTSD symptoms, except that the trauma was experienced indirectly by hearing about or knowing about a traumatic situation. Overall, the aims of the current research are to highlight many of the above-indicated issues suggested by pervious research work; and to examine the concept, the prevalence, the main etiology and, effective treatment approaches used for compassion fatigue and secondary traumatic stress among a sample of healthcare providers working in hospitals in London.

### Shattered assumptions in trauma assistants 15:45–16:00

#### P. Andreatta University of Innsbruck, Innsbruck, Austria

“Disasters and crises tap into our deepest fears, setting in motion the struggle for survival and the pain and suffering that go along with it. No one who participates in such an experience, including the helpers, can escape being affected by it.” (Charney & Pearlman, 1998). This presentation focuses on the social-cognitive aspects of facilitating the development and maintenance of PTSD within secondary traumatization. Our cognitive worlds consist of theories and working models including beliefs about ourselves, the external world, and the relationship between the two. This approach is theoretically approached in the “Assumptive Worlds” of Janoff-Bulman (1992). Working with victims of disasters can shatter the helper's own fundamental concepts and leads to an abrupt disintegration of the inner world. Helpers are at-risk for secondary traumatization, which is mainly facilitated by the role of empathy (Figley, 2002). Quantitative data (N=131) among a sample of trauma assistants working in crises intervention and paramedics were collected. To examine the effects of traumatic stress the posttraumatic stress diagnostic scale (Foa, 1995) was used and to survey fundamental beliefs and view of the self and the world the World Assumptions Scale of Janoff-Bulman (2007). Results show changes within cognitive schemas regarding the participant's world and self-view concerning fundamental assumptions about the meaningfulness of life, benevolence of people, control, self-worth, and assumptions about justice. Trauma assistants show more impact on their fundamental assumptions than paramedics, which can be discussed form the perspective of empathy. On top of that, a specific pattern of disruption in schemas was found. This pattern can be either interpreted to function as psychodynamic defense and/or to be a hint for posttraumatic growth.

### Psychological trauma and other stressors affecting the mental health of local staff working in the Vanni region in Sri Lanka 16:00–16:15

#### B. Lopes Cardozo^1^, T. Sivilli^2^, C. Crawford^1^, W. Scholte^3^, P. Petit^3^, F. Ghitis^3^, A. Ager^4^ and C. Eriksson^5^

^1^Centers for Disease Control and Prevention, Atlanta, USA; ^2^Department of Psychiatry, Emory University, Atlanta, USA; ^3^Antares Foundation, Amsterdam, The Netherlands; ^4^Columbia University, New York, USA; ^5^Fuller Theological Seminary, Pasadena, USA

In the aftermath of the civil war in Sri Lanka that extended from 1983 to 2009, humanitarian organizations provided aid to the conflict-affected population of the Vanni region in northern Sri Lanka. Little is known about the consequences of the stress of humanitarian aid work on national staff, even though they make up the majority of the workforce in many humanitarian organizations. In August 2010, we conducted a needs assessment to determine the mental health status of Sri Lankan national humanitarian aid staff working in post-war conditions of stress and hardship, and consider cultural, contextual, and organizational characteristics influencing such status. A total of 398 staff members from nine organizations working in the Vanni area participated in the survey, which assessed stress, work characteristics, social support, coping styles, and symptoms of psychological distress. Exposure to traumatic, chronic, and secondary stressors was common. Nineteen percent of the population met the criteria for posttraumatic stress disorder (PTSD), 53% of participants reported anxiety symptoms, and 58% reported depression symptoms. Those reporting high levels of support from their organizations were less likely to suffer depression and PTSD symptoms than those reporting lower levels of staff support (*OR=*0.23, *p<*0 .001) and (*OR*=0.26, *p*<0.001), respectively. Participants who were age 55 or older were significantly less likely to suffer anxiety symptoms than those who were between 15 and 34 years of age (*OR*=0.13, *p=*0.011). Having experienced travel difficulties, including threatening checkpoints, and rough roads, was significantly associated with more anxiety symptoms (*OR*=3.35, *p*<.001). We recommended that humanitarian organizations provide stress management training and increase support to their staff. Best practices to address high levels of depression and anxiety symptoms in these workers needs to be explored further.

## Open Papers: The spectrum of trauma related disorders

### The co-occurrence of PTSD and dissociation: differentiating severe PTSD from dissociative PTSD 17:00–17:15

#### C. Armour^1^, K. Karstoft^1^, A. Elklit^1^ and D. Richardson^2^

^1^University of Southern Denmark, Odense, Denmark; ^2^Operational Stress Injury Clinic, St. Joseph's Health Care London, University of Western Ontario, London, Ontario, Canada; National Center for Operational Stress Injury Canada

Recent studies have suggested that distinct subgroups of PTSD may exist based on their level of dissociation, indeed a dissociative PTSD subtype has been suggested for the DSM-V. However, the nature of the relationship between dissociation and PTSD remains unclear. Furthermore, it is not yet clear whether certain characteristics and experiences differentiate between severe PTSD and dissociative PTSD. The current study investigated the co-occurrence of dissociation and posttraumatic psychopathology in a sample of 432 treatment seeking Canadian military veterans. Participants were assessed with the clinician administered PTSD scale (CAPS) and self-report measures of traumatic life events, depression, and anxiety. CAPS severity scores were created reflecting the sum of the frequency and intensity items from each of the 17 PTSD and three dissociation items. The CAPS severity scores were applied to latent profile analysis (LPA). Subsequently, several covariates were added to the model. The LPA identified five classes: two low PTSD severity subgroups (13.7%, and 20.0%, respectively), an intermediate PTSD severity group (22.1%), a severe PTSD group (30.5%), and a dissociative PTSD group (13.7%). The experience of sexual assault (i.e., attempted rape, made to perform any type of sexual act through force or threat of harm) was the only covariate which was significantly predictive (OR=2.733; CI=1.253–5.967) of membership in the dissociative PTSD group compared to the severe PTSD group. The participants from this all-veteran sample were assessed by the same clinician. Furthermore, the study was retrospective and included a relatively narrow measure of dissociation. In conclusion, a significant proportion of individuals experienced high levels of dissociation alongside their PTSD, which may constitute a dissociative-PTSD subtype. Sexual assault may increase the likelihood of experiencing a dissociative-PTSD subtype compared to severe PTSD alone.

### The amputated fingers: reflections on co-therapy in differentiated time 17:15–17:30

#### O. Convertino^1^, M. Lanzetta^2^, G. Urso^3^, E. Berardi^1^, D. Sala ^1^, F. Pirovano^1^, F. Porco^1^, S. Arrigoni^1^, C. Recanati^1^ and V. Matacchiera^1^

^1^Studio Convertino, Monza, Italy; ^2^Italian Institute of Hand Surgery, Monza, Italy; ^3^Rehabilitation Centre “Il Carrobiolo”, Monza, Italy

The paper aims to examine two cases with amputation of the fingers as a result of workplace accidents. We will consider, through psychodiagnostic tests, the trauma's influence entailment of the redefinition of the body schema and the symbolic value of the loss of fingers. The approach of “co-therapy at differentiated time by multidisciplinary setting” has the objective of helping patients to revise the posttraumatic stress disorder by drawing on various techniques that aim to integrate the new body schema, the new symbols connected to it, activated from behavioral strategies and processes of identity. The hand assumes a central life significance for the individual: an amputation is not only an motor expressive impairment, but it compromises psychic mechanisms related to self-definition and the representation of reality inside and outside the person. The individual defines himself according to the perception of the body (body schema) and the features that it can perform. The method is based on diversified and integrated techniques (role playing, collage, personal empowerment etc.) elaborated by the team of professionals after the analysis of the final test results. The multidisciplinary team operates with a shared procedure based on the analysis of transference and countertransference in different settings. In conclusion, the “co-therapy at differentiated time by multidisciplinary setting” creates, from the integration of aspects of the co-therapist's countertransferal and the patient's symbolic process, a new and effective technique under different settings.

### PTSD and anxiety and depression after exposure to physical assault, through 8 years 17:30–17:45

#### V. A. Johansen^1^, D. E. Eilertsen^2^, D. Nordanger^1^ and L. Weisaeth^3^

^1^Resource Center on Violence, Traumatic Stress and Suicide Prevention, Haukeland University Hospital, Bergen, Norway; ^2^Department of Psychology, Center for the Study of Human Cognition, University of Oslo, Oslo, Norway; ^3^Faculty of Medicine, Institute of Clinical Medicine, University of Oslo, Oslo, Norway

*Background*: There is a lack of prospective longitudinal studies focusing on the victims exposed to physical violence by a perpetrator other than a family member. *Aims*: To assess the prevalence and comorbidity of PTSD and anxiety and depression symptoms, and the stability of symptoms, in a sample of victims of non-domestic violence exposed to physical assault, through 8 years. *Method*: This study had a single group longitudinal design with four repeated measures, the first as soon as possible after the exposure (*N*=143 at T1), the second 3 months later (*N*=94 at T2), the third after one year (*N*=73 at T3) and the fourth after 8 years (*N*=47 at T4). Questionnaires used were: impact of event scale-15 and 22 (IES-15 and 22), posttraumatic symptom scale-10 (PTSS-10) and the Hopkins symptoms check list (HSCL-25). *Results*: Probable PTSD cases measured with IES-15 were found to be 33.6% at T1, 30.9 at T2, 30.1% at T3 (12 months), and 19.1% at T4 (8 years), while probable anxiety and depression cases measured with HSCL-25 were 42,3% at T1, 35,5% at T2, 35,6% at T3, and 23,4% at T4. The comorbidity of probable PTSD and probable anxiety and depression symptoms were high, the values ranged from 87.5% at T1 to 55.6% at T4. The estimated probability of recovery during the 8 years from PTSD symptoms is 52% while the corresponding findings concerning anxiety and depression are 43%. *Conclusion*: The high occurrence of both PTSD symptoms and anxiety-depression after 8 years shows that exposure to physical assault by strangers need to be given more attention as a severe risk of chronic mental health problems. Clinicians have to be aware of individual experience and symptoms when offering follow-ups and psychological treatment.

### Long-term consequences of armed conflicts: the burden of mental health disorders and factors influencing coping among conflict-affected populations in Georgia 17:45–18:00

#### N. Makhashvili^1^, I. Chikovani^2^, M. McKee^3^, V. Patel^3^, J. Bisson^4^, N. Rukhadze^2^ and B. Roberts^3^

^1^Global Initiative on Psychiatry-Tbilisi, Ilia State University, Tbilisi, Georgia; ^2^Curatio International Foundations, Tbilisi, Georgia; ^3^London School of Hygiene and Tropical Medicine, London, UK; ^4^Cardiff University School of Medicine and Cardiff and Vale University Health Board, London, UK

*Background*: There are high numbers of internally displaced persons (IDPs) in the Republic of Georgia as a result of armed conflicts over the past two decades. *Methods*: A cross-sectional household survey conducted in late 2010 using multistage random sampling of conflict-affected persons aged ≥18 years from three war-affected groups: displaced in early 1990s (Old IDPs); displaced in 2008 (New IPDs) and those who returned to border-line villages after being initially displaced from their homes (Returnees). Outcome measures included PTSD, depression, anxiety, and functional disability. *Results*: The data shows that almost a quarter (24%) of IDPs met criteria for PTSD, 14% for depression, and 11% for anxiety. When limited to only respondents who had any mental health condition, 42% of them had co-morbidity of these disorders. The burden of disability was also substantial: a 14% increase in disability was related to PTSD, 20% to depression and, 19% to anxiety, with women significantly more likely to report higher disability scores than men. The influences of time and coping mechanism with trauma were also examined. *Conclusions*: The paper explores patterns of mental health conditions among IDPs and returnees and presents range of factors associated with them, and their associated burden with disability, including coping factors. The policy implications are also discussed and recommendations made on establishing appropriate mental health and trauma services for large groups of IDPs.

## ORAL, JUNE 7 HALL LIZ

## Open Papers: Approcci di cura e trauma

### Psychotherapy of trauma and resources of the patient: theoretical and practical aspects in the psychotherapeutic practice 17.30–17.45

#### Marialfonsa Fontana Sartorio Associazione Qualità e Formazione, Milan - Sipst, Milan, Italy

From the most recent studies in neuroscience, psychology, and Psychotherapy, it is known that any elaborated crisis or conflict gets registered in our brain as a new experience, and changes our knowledge and behaviour. We addressed this issue in the psychotherapy of trauma and also the ways to facilitate this process and strengthen the patient's specific competencies dealing with anxiety and trauma.

It is very important to work on the activation and reinforcement of personal resources, both in the elaboration of trauma and as a preventive measure, because extreme impotence activates ego states related to feelings of helplessness and despair, as the traumatic experience undermines the positive image of ourselves.

This leads to narrowing of perception and consequently inability to evaluation. The brain will no longer be able to find creative solutions: a situation that is consolidated in the presence of PTSD, when the process of elaborating experiences is blocked, frozen in a perpetual present causing an obstacle to the free unfolding of the individual creativeness.

For this purpose, we mentioned consolidated intervention tools: Psychoenergetic Drawing^®^ (www.qualitaeformazione.com), which is based on the positive and projecting aspect inherent in the concept of complex of Jung, and the TRUST-Resilienz-Training/TRUST-RT^®^ of Christa Diegelmann and Margarete Isermann (www.idinstitut.de) recognized as formative course at the Deutsche Psychologen Akademie from 2012, both designed to strengthen the psychic resilience.

It does not deal with a hypothetical concept of ‘health’; on the contrary, it deals with the ways to distinguish well-being and post-traumatic growth, that is to say, all those positive changes that result from the processing of the traumatic event, trusting thereby in the potentialities of individuals to integrate trauma in their own world and to find a more complex balance to continue their process of personal identification.

## POSTERS, JUNE 7

## Psychobiology and trauma

### Resilience to the development of posttraumatic stress disorder associated with common KIBRA alleles

#### S. Wilker^1^, S. Kolassa^2^, C. Vogler^3^, B. Lingenfelder^4^, T. Elbert^4^, A. Papassotiropoulos^3^, D. De Quervain^5^ and I. Kolassa^1^

^1^Clinical & Biological Psychology, Institute for Psychology & Education, University of Ulm, Ulm, Germany; ^2^SAP Switzerland AG, Tägerwilen, Switzerland; ^3^Division of Molecular Neuroscience, University of Basel, Basel, Switzerland; ^4^Department of Psychology, University of Konstanz, Konstanz, Germany; ^5^Division of Cognitive Neuroscience, University of Basel, Basel, Switzerland

*Background*: The core feature of posttraumatic stress disorder (PTSD) is a strong but defragmented memory for the traumatic events survived. Genetic factors involved in (emotional) memory formation have been repeatedly found to influence vulnerability to PTSD development subsequent to trauma exposure. Because accumulating evidence from behavioral and biomolecular studies indicates that the gene encoding brain protein KIBRA is involved in long-term memory performance, we hypothesized that common *KIBRA* alleles influence the susceptibility of lifetime PTSD development. *Methods*: We employed a structured clinical interview to assess traumatic load and current and lifetime PTSD in two independent samples of survivors from genocide (N=392, Rwanda) and civil war experiences (N=399, Northern Uganda). DNA was isolated from saliva samples and chip-based single nucleotide polymorphism (SNP) genotyping was performed. We fitted logistic regression models with correction for multiple comparisons to evaluate the influence of 115 tagged *KIBRA* SNPs on current and lifetime PTSD in the Rwandan discovery sample. Hypothesis-driven replication analysis was performed in the Ugandan sample employing the same statistical model. *Results*: We discovered an association of two *KIBRA* SNPs, rs10038727 and rs4576167, in near complete linkage disequilibrium with lifetime PTSD in the Rwandan sample and replicated this association in the independent Ugandan sample. Traumatic load increased the likelihood of PTSD development in both genotype groups; however, carriers of the minor allele of rs10038727 and rs4576167 had significantly reduced risk to develop PTSD across all levels of traumatic load. *Discussion*: We identified a protective effect of the minor allele of rs10038727 and rs4576167 in two independent samples. The results of this study indicate that the gene encoding KIBRA influences the likelihood of PTSD development through its impact on long-term memory processes.

### Childhood trauma, PTSD, and physical health

#### N. B. Hansen, S. Palic, T. Andersen, S. Roenholt, S. Karsberg and A. Elklit National Research Centre for Psychotraumatology, University of Southern Denmark, Odense, Denmark

Associations between childhood trauma and adult mental health problems like depression, anxiety, and posttraumatic stress disorder (PTSD) are especially emphasized in the literature. Although less extensively researched, childhood traumatic experiences have been hypothesized to increase the risk of adult onset of a spectrum of chronic physical diseases. A recent meta-analysis of the effects of child abuse on medical outcomes in adulthood found that the increased risk of selected adverse physical health outcomes was comparable to that observed for poor mental health outcomes. Unfortunately, the literature suffers from methodological shortcomings that limit the more specific understanding of the relationship between the childhood trauma and physical health. The effects of neglect and emotional abuse are underrepresented in the literature, whereas childhood sexual abuse and childhood physical abuse are overrepresented. The potential mediators (e.g., behavioural, physiological, and psychological) of the relationship are neither well understood nor well researched, and some common health outcomes, such as an unhealthy low body weight, are almost entirely overlooked in the literature. On this background, we present the results of a stratified random probability survey conducted in Denmark with 2981 participants born in 1984. Our results showed that PTSD symptomatology and childhood abuse were significantly associated with both underweight and overweight/obesity in adulthood. Furthermore, childhood emotional abuse was especially associated with underweight, whereas sexual abuse and overall abuse were particularly associated with overweight/obesity. Also, we found that childhood abuse was significantly associated with poorer self-reported physical health status in adulthood. Psychological distress and health risk behaviours partially mediated the relationship between no abuse, sexual abuse, and physical abuse and health problems and fully mediated the relationship between emotional abuse and physical health. These results indicate that early interventions following adverse childhood experiences are important to prevent some of the long-lasting consequences on adult physical health.

### Validity of Impact of Event Scale-6—Portuguese version

#### A. Lopes, J. Rocha, V. Bastos, B. Frade, E. Ferreira, F. Afonso and D. Pacheco UnIPSa-CICS, InstitutoSuperior de Ciências da Saúde—Norte, CESPU, Gandra, Portugal

The posttraumatic stress disorder (PTSD) is characterized by specific symptoms that individuals develop after exposure to traumatic events and respond with intense fear, helplessness, or horror. The value and limitations of brief screening procedures is well known for traumatic stress. However, its usefulness depends on adapting instruments for native languages and its application being disseminated on practice contexts. The purposes of this study are (1) to develop an abbreviated version of the Impact of Event Scale-Revised (IES-R) based on the study of Thoresen and colleagues and (2) to calculate the cutoff of the IES-R-6. The sample has 520 participants, 147 (28.3%) men and 373 (71.7%) women. The average age is 28.72 (SD=12.61) years. The instruments used were the Portuguese versions of Impact of Event Scale – Revised (IES-R) and the Clinician-Administered PTSD Scale (CAPS). To accomplish the abbreviation IES-R for the Portuguese version, we use regression enter method to prove the hypothesis from Thoresen and colleagues. The procedure for abbreviation results in a subset of six items (IES-6), which was correlated with the IES-R (pooled correlation=0.951). Using CAPS as gold standard, the cutoff for IES-6 is 12.5 and for IES-R is 35.5. The Cronbach's alpha calculated for the IES-6 is 0.84. The IES-6 has good psychometric qualities and is a good instrument for PTSD screening, and also is a robust brief measure of posttraumatic stress reactions. Our data demonstrate that the IES-6 is a reliable measure to assess PTSD; however, it should be used with an interview to acknowledge the complexity of traumatic stress. The specific utility of both IES-R and IES-6 on several contexts is discussed considering future developments.

## Miscellaneous

### Transformation performance: a dramaturgical methodology for the processing of traumatic memory

#### S. P. Philip Edge Hill University, Ormskirk, Lancashire, UK

This article examines the potential of performance as a transformative event, realigning a wounded psyche by enabling embodied dialogues between self and traumatic memory through the application of a nascent methodology, “Transformation Performance” (T/P). It considers the postadoption “self” as a posttraumatic “self,” making critical connections between the two, and proposes that the adoption process is characteristically traumatic in nature, resulting in personal and cultural dislocation. The research questions the ontological nature of psychic trauma as an invasive, compromising agent, exploring how an etiological strategy of temporal disruption, intervention through creative process and alternate memory planting through performed narrative, might operate to address the characteristically oppressive nature of traumatic memory instigating a process of transformation in the adopted individual. The research design is guided by a geological metaphor: the psyche is distorted around ley-lines—traces of historic trauma—connecting and influencing public representations of “self”. T/P resolves these “misrepresentations” through a supported creative process, which seeks to liberate the psychic and physical “self”. This practice-led research originates in responses developed in reflection on work undertaken with recovering addicts in 2009, the personal testimonies of American and British military personnel collected in 2010–2011, alongside my own heuristic questioning and autobiographical deconstruction, in search of a posttraumatic performance of the liberated postadoption “self”. The research considers the work of Dr. Francine Shapiro and will hypothesise that the methodology of T/P offers a comparable creative approach to the processing of traumatic memory. The experience of T/P is one of “living through” posttrauma, and not of transcendence, on one hand, or “coping”, on the other. Accordingly the “self”, arrived at by means of T/P is hypothesised as a publicly articulated site of personal history reclaimed through performance as a subaltern consciousness, articulated and liberated by the enabled narrative voice.

### Factorial structure and invariance of the Posttraumatic Symptom Scale (PTSS-10) in patients with posttraumatic stress disorder

#### M. Iskenius^1^ and R. Bering^2^

^1^Bergische Universität Wuppertal, Germany; ^2^Center of Psychotraumatology, Krefeld, Germany

*Background*: Posttraumatic Symptom Scale (PTSS-10) detects multiple symptoms of posttraumatic stress disorder (PTSD). The results for the factorial structure of the scale are inconsistent and it is not known to what extent the scale exhibits measurement invariance. *Objective*: Different factor structure models of the PTSS-10 scale were tested, and various forms of measurement invariance examined. *Methods*: A total of 247 subjects with a diagnosis of PTSD were examined on two time points. The factor structure was determined by means of exploratory and confirmatory factor analysis. The invariance of the PTSS-10 scale was tested by confirmatory factor analysis with increasing restrictions. *Results*: A two-factor structure was identified; the first factor reflected a general mental instability, while the second factor included classic symptoms of PTSD. For this model, strong measurement invariance was observed. *Conclusions*: The PTSS-10 scale is a screening tool that is appropriate for the evaluation of changes in PTSD symptoms.

### Posttraumatic stress disorder in the elderly in Poland

#### M. Lis-Turlejska^1^ and S. Szumial^2^

^1^Warsaw School of Social Psychology and Humanities, Warsaw, Poland; ^2^Caritas Community Self-Help Home, Minsk Mazowiecki, Poland

*Background*: The studies on the elderly people who survived WWII in Europe show the negative impact of exposure to war-related traumatic events on mental health even after a long time. The prevalence of posttraumatic stress disorder (PTSD) and other trauma-related symptoms and disorders differ between the countries. Despite the issue of long lasting consequences of WWII-related traumas, there is also a challenge to explain the differences between particular countries. *Objective*: The aim of the study was to estimate prevalence of PTSD and other trauma-related symptoms among the Polish elderly who survived the WWII. *Method*: There were two studies conducted in 2007–2010 (N=218) and in 2012 (N=177). All participants were born before 1945. Measures: PDS, IES, BDI, GHQ-12, NHP, a questionnaire with the items addressing exposure to different types of war-related traumatic experiences and a scale measuring perceived negative impact of the WWII experience on one's life as a whole. *Results*: Prevalence rate of PTSD in study I was 29.4% and in study II was 33.7%. Mean values of both number and severity of symptoms of PTSD were significantly higher for respondents with at least one war-related trauma compared to the participants who did not relate any such trauma. Among the predictors of PTSD were older age, experiencing loss of parent during the war, and experiencing at least one war-related trauma. *Conclusions*: Compared to other studies on WWII-related PTSD in other countries, the level of PTSD is very high. It is important to explain the factors contributing to such results.

### The role of peritraumatic dissociation, anxiety level, and perceived controllability in development of PTSD symptoms following childbirth

#### K. Geronalowicz Warsaw School of Social Sciences and Humanities, Warsaw, Poland

It is still controversial whether childbirth should be included as a traumatic happening. Unlike other traumatic experiences, childbirth is not sudden; however, there are many factors that bear signs of trauma. Strous et al. (2012) showed that the degree of experienced labor pain has a direct impact on woman's well-being and a desire of having more children in future. Most of examined women who developed PTSD symptoms had a natural delivery without analgesics. In this study, it was assumed that PTSD depends on level of dissociation, anxiety level during labor, perceived sense of control before and during childbirth, level of experienced labor pain, and previous traumatic experiences. This study involved two groups of women which differed in a type of delivery (natural without analgesic vs. labor with analgesic). Carried statistical analysis showed significant differences between those two groups directly after birth and three months after delivery.

## Cultural issues and trauma

### Basic assumptions in different groups of trauma patients

#### R. Broekhof, A. Smith and N. Van Der Aa Arq Research Program, Foundation Centrum '45, Oegstgeest, The Netherlands

The core of trauma is an experience that is outside the range of normal human experiences. As a result of the traumatization, existing basic assumptions about oneself and the world are invalidated. In psychotherapeutic treatment of posttraumatic psychopathology, telling and reliving the traumatic experiences in a safe environment entails also a revision of these basic assumptions. Trauma context, cultural background, and forced migration may be associated with differences in basic assumptions. Earlier results showed that war victims reported high levels of global and low levels of personal assumptions concerning justice, control, and predictability (Smith, 2005). Children of war-traumatized parents reported lower levels ofworld assumptions (Smith, 2005). Veterans with traumatic exposure reported lower levels of self-worth and benevolence of people than non-exposed veterans (Dekel et al., 2004). The aim of our study is to compare the characteristics of basic assumptions in refugees, Second World War victims, children of war victims, and veterans. Data were obtained from patients referred to Foundation Centrum '45 at intake (n>100 patients per subgroup). The World Assumptions Scale (Janoff-Bulman, 1989), a self-report scale, was used to examine participants'cognitive schemes about themselves and their world.The scale consists of eight subscales: benevolence of the world and people, just world, controllability, randomness, self worth, self-controllability, and luck. The study aims to access differences in basic assumptions about oneself and the world in patients with different traumatization contexts and possible implications of these differences for intervention purposes.

References

Dekel, R., Dolomon, Z., Elklit, A., & Ginzburg, K. (2004). World assumptions and combat-related post traumatic stress disorder. *The Journal of Social Psychology*, *144*(4), 407–420.

Janoff-Bulman, R. (1989). Assumptive worlds and the stress of traumatic events: applications of the schema construct. *Social Cognition, 7*, 113–136.

Smith, A. J. M. Cognitive reactions to war trauma. Poster presentation, unpublished 2005.

### Torture and sex: the present in which women and men who survived the camp live

#### A. Arnautovic Vive Zene Center for Therapy and Counseling, Tuzla, Bosnia & Herzegovina

Torture is a series of traumatic events focused on the individual and directed to break down and revert personality with cruel, deliberate, and systematic application of psychological violence, or violence against the body, but often a combination of both. According to the principles of international humanitarian law and international human rights laws, victims of torture in Bosnia and Herzegovina have, due to 1992–1995 armed conflict, three categories recognized: All persons subjected to torture during the conflict (camps and illegal detention centers); victims of rape and other forms of sexual violence (identified form of torture as an instrument of torture and crimes against humanity—by the ICTY); families of the missing—only until the identification of the missing. All survivors of torture live with a lifelong trauma. Unacknowledged status, unemployment, housing, disorganized legal framework are just some of the additional stressors. A comprehensive rehabilitation plan is required. Those who survived torture in Bosnia face discrimination because they cannot achieve their basic rights under the existing legislation. Officially, women are in the same position with men, but in practical life situation has another dimension. Social stereotypes and traditional views on the roles of men and women are entrenched. Throughout and after the war, the situation changed and complicated the position of both sides regardless of whether they were exposed to the same extreme traumatic situation, torture and rape. The author attempted to illustrate the similarities and differences existing in the manifested symptomatology, the family and society circumstances, and daily functioning of women and men who have an extremely traumatic camp experience in common.

Keywords: *Torture*; *gender discrimination*

### SRGS and SZŻ—questionnaire measurements of posttraumatic growth

#### M. Wawrzyniak, M. Zieba and M. Malinowska University of Social Sciences and Humanities, Warsaw, Poland

More and more research point to the fact that many people develop significantly after stressful experiences (Park, 1998). For several years, the subject of systematic study is the phenomenon of posttraumatic growth (Calhoun & Tedeschi, 2004; Linley & Joseph, 2004; Tedeschi & Calhoun, 1996, 2004; Tedeschi, Park, & Calhoun, 1998), defined as “the experience of positive change that occurs as a result of the struggle with difficult or traumatic life events, this is expressed by appreciation and increase the value of life, enhanced interpersonal relationships, increased sense of personal strength, change the system of values, and enrich the spiritual life” (Tedeschi & Calhoun, 2004, p. 1). The beginning of the process of posttraumatic growth is a major event in life that undermines the basic patterns of the world, including assumptions about the predictability and the controllability, as well as those core beliefs about oneself and the meaning of their existence (Janoff-Bulman, 2004). Since the first conceptualization of posttraumatic growth, there is a challenge for researchers to create a good tool for measuring this phenomenon. To measure the posttraumatic growth, questionnaires and various qualitative techniques are most commonly used, which are based on a structured interview and narrative techniques (Neymeier, 2006). Among the methods for quantitative measurement of posttraumatic growth, two questionnaires seem to be the most popular: Posttraumatic Growth Inventory (PTGI) developed by Tedeschiego and Calhoun (1996) and Stress-Related Growth Scale (SRGS) developed by Park, Cohen, and Murch (1996). The poster presents the results of work on the Polish adaptation of the SRGS (Park, Cohen, & Murch, 1996) and the authors questionnaire Life Change Inventory.

### Cultural competence training: a pilot program for providers working with trauma-exposed lesbian, gay, bisexual, transgender, and questioning youth in America

#### S. Strahl and I. Seilicovich The Village Family Services, North Hollywood, USA

Lesbian, gay, bisexual, transgender, and questioning (LGBTQ) youth are at a higher risk of depression and suicide compared to their heterosexual peers (Marshal et al., 2011). They experience an increased exposure to chronic stress, bullying, rejection, and other trauma(D'Augelli, Hershberger, & Pilkington, 2010). There is a critical need for culturally competent providers to work with trauma-exposed youth; yet, asset mapping reveals a lack of training resources on how to provide LGBTQ sensitive and affirming services. *Working with LGBTQ Youth: What You Really need to Know* is a pilot provider training program that was funded by the Los Angeles County Department of Mental Health. In 2012, The Village Family Services created, tested, and implemented a 9 module, 11-hour training to 201 service professionals across 10 locations in Los Angeles County. The program included 7 hours of interactive classroom training paired with 2 hours of technical assistance and 2 hours of onsite coaching. In addition, a group of LGBTQ youth advocates was recruited and trained, and each classroom session included the active participation of at least one youth advocate. Evaluation results indicate improved provider ability to deliver appropriate, sensitive, and affirming services to LGBTQ transitional age youth. Moreover, organizational change occurred including modification of policies and an increase in the creation of safe spaces.

References

D'Augelli, A. R., Hershberger, S. L., & Pilkington, N. W. (2010). Lesbian, gay, and bisexual youth and their families: Disclosure of sexual orientation and its consequences. *American Journal of Orthopsychiatry*, *68*(3), 361–371.

Marshal, M. P., Dietz, L. J., Friedman, M. S., Stall, R., Smith, H. A., McGinely, J., et al. (2011). Suicidality and depression disparities between sexual minority and heterosexual youth: A meta-analytic review. *Journal of Adolescent Health, 49*(2), 115–123.

## Responding to disasters

### An example of solidarity: studies of Turkish Psychiatric Association and Union of Disaster Psychosocial Services after the Van and Erciş Earthquakes

#### H. S. Kalkan^1^, F. Celik^2^ and T. Aker^2^

^1^DiyarbakIr Ergani State Hospital, Turkey; ^2^University of Kocaeli, Turkey

Studies conducted after disasters happened within the last decade have shown that the need of psychosocial interventions were much more important than it was supposed before. There is a consensus on the view that psychosocial interventions are crucial to prevent the development of mental disorders that may arise in the future. Two big earthquakes happened in Turkey consecutively: the first one was in Erciş on the 23 September 2011,and the second was in Van on the 9 November 2011, with the magnitudes of 7.2 and 5.6, respectively. Six hundred and forty-four people died, 4152 people were injured, 252 people were rescued, a vast number of buildings damaged, and approximately one million people were adversely affected by the earthquakes. The aims of this study were to mention and assess the psychosocial interventions as presented by the Turkish Psychiatric Association (TPA) along with the Union of Disaster Psychosocial Services (UDPS) after the Van and Erciş Earthquakes.

### Parenting after terror—experiences with an outreach program after the July 22 terror attack at Utøya Island

#### K. A. Glad^1^, T. K. Jensen^2^ and G. Dyb^1^

^1^Norwegian Centre for Violence and Traumatic Stress Studies, Oslo, Norway; ^2^University of Oslo, Oslo, Norway

*Background*: On 22 July 2011, Norway experienced two sequential terrorist attacks against the government, the civilian population, and an island summer camp for young members of the governing Labor Party. Questions immediately arose on how the health authorities should respond to help the directly affected. Within days, a national, proactive outreach strategy was developed and implemented in Norwegian municipalities. Little evidenced-based knowledge exists on how outreach strategies guidelines after mass trauma are implemented and how well they function. *Objective*: The aim of this study is to find out how the parents of the youth who were at Utøya experienced the outreach strategy and whether or not they felt that their family's needs had been adequately addressed. *Method*: Approximately one year after the attack on the island of Utøya, parents (N=405) were asked whether or not they had unmet needs for (1) their child who had been at Utøya, (2) themselves, (3) other family members, or (4) the family as a whole. They were also asked to give a short, written description of their unmet needs. *Result*: Descriptive analysis of the number of parents who reported unmet needs and a thematic analysis of the type of unmet needs reported will be presented. *Implications*: The parents’ experiences can contribute to our knowledge about how to successfully implement outreach programs after mass trauma. In this poster presentation, the outreach strategy will be briefly described along with results and possible implications.

### Posttraumatic growth in connection to profession-related traumatization

#### Anna Krutolevich^1,2^

^1^Gomel Engineering Institute of the Ministry for Emergency Situations of the Republic of Belarus, Gomel, Belarus; ^2^University of Muenster, Münster, Germany

*Background*: The work of emergency services is closely connected with the experience of serious injuries or death, severely disfigured dying victims as well as with emotional contacts with patients and their families. This can lead to the development of secondary traumatization. Some rescue workers see in their routine job the source of their own posttraumatic growth and increase their own appreciation of life. The carried out studies on PTSD and posttraumatic growth showed mixed results regarding to the relations between the two variables and their influence factors. *Aim*: The aim of this study is to examine how high is the percentage of those who use the traumatic experience as a source of work-related trauma positive reevaluations and their appreciation of life and how strong is the influence of posttraumatic growth on the general mental health of rescue workers. In addition, the variables included in the analysis, which have a potential influence on the development of posttraumatic growth, are studied. *Method*: The data were obtained from 53 Belarusian paramedics and 115 firefighters. *Results*: The study showed a positive relationship between the secondary traumatization and posttraumatic growth. The perceived growth processes do not involve mental health and are strongly influenced by the self-efficacy, disclosure and co-rumination. The sociodemographic factors “professional group” and “gender” correlate significantly to posttraumatic growth. It is shown that women and paramedics tend strongly to posttraumatic growth than men and firefighters. At the same time, these groups are usually more traumatized.

### Predicting coping styles in adolescence following trauma

#### D. Christiansen^1^, M. Hansen^2^ and A. Elklit^2^

^1^Institute of Psychology, Aarhus University, Aarhus, Denmark; ^2^National Centre for Psychotraumatology, Institute of Psychology, University of Southern Denmark, Odense, Denmark

Decades of research have established the importance of coping when dealing with a stressful or traumatic event. Individuals tend to use the same overall coping styles across situations, and correlational studies have demonstrated a relationship between individual characteristics and coping. However, there is a lack of research investigating the interplay between these individual characteristics and their combined effect on different coping styles. It is of special importance to identify maladaptive coping styles in adolescents because they may be prone to use these coping styles for the rest of their lives. This study used a cross-sectional design to investigate the combined effect of personality traits, attachment, locus of control, and social support on rational (problem-focused), avoidant, and emotion-focused coping in 320 students (females n=199) attending a Danish high school, where a female student was killed. Combined, the variables accounted for 19% of the variance in problem-focused coping, 21% of the variance in avoidant coping, and 49% of the variance in emotion-focused coping. The fact that the independent variables could account for a substantially larger amount of the variance in emotion-focused compared to rational and avoidant coping is likely due to a confounding of emotion-focused coping with distress, which affects many of the most commonly used coping measures, including the CSQ used in this study. This study points to the importance of conducting regression analyses rather than relying exclusively on correlational research. The results suggest that personality traits and attachment can account for some of the variance in coping styles, but that a large amount of the variance remains to be accounted for. A combination of individual and situation-specific characteristics is likely to be necessary to account for the remaining variance in the use of coping styles.

### Understanding trauma survivors with their autobiographical trauma memory profiles

#### H. Joo and H. Ahn Department of Psychology, Ewha Womans University, Seoul, Korea

Among the autobiographical memory aspects that were found to be related to trauma symptoms, the following seems to have strong empirical support; (1) coherence, (2) accessibility, (3) sensory detail, and (4) vividness. The purpose of this study was to explore what patterns of the autobiographical memory of trauma exist, and to examine how such different patterns of autobiographical memory of trauma relate to posttraumatic stress disorder (PTSD) and to trauma-related appraisals. Data from 115 Korean college students who had reported at least one traumatic event or negative stressful event within past three years were analyzed. The participants answered the items from the Memory Experiences Questionnaire (Sutin & Robins, 2007), Posttraumatic Diagnostic Scale (Foa, Cashman, Jaycox, & Perry, 1997), and Trauma Appraisal Questionnaire (DePrince, Zurbriggen, Chu, & Smart, 2010). We performed a two-step cluster analysis and specified that a four-cluster solution was valid; (1) cluster 1 (high accessibility and vividness with low coherence and sensory detail), (2) cluster 2 (low coherence, accessibility, sensory detail, and vividness), (3) cluster 3 (high coherence, accessibility, sensory detail, and vividness), and (4) cluster 4 (high coherence, accessibility, and vividness, with low sensory detail). Then, we investigated the differences among clusters on posttraumatic appraisals by using MANOVA. As a result, clusters 1 and 3 showed higher betrayal, self-blame, alienation, anger, and shame than cluster 4, and cluster 3 represented higher fear than clusters 2 and 4. Also, cluster 3 showed the most severe PTSD symptoms than other clusters. Such results indicated that while accessibility and vividness of the trauma memory were most consistently related to the primary emotional symptoms, the other memory characteristics may have more complicated contribution to trauma-related emotions. Coherence and sensory detail were inconsistent with the previous findings, and we discussed the possible reasons such as the demographic characteristics of our sample.

## The spectrum of trauma-related disorders

### Dissociation as a mediator between childhood trauma and depression among women with fibromyalgia or rheumatoid arthritis

#### V. Sar^1^, O. Kilic^2^, O. Taycan^3^, C. Aksoy-Poyraz^2^, T. C. Erol^4^, O. Tecer^5^, M. Emul^2^ and M. Ozmen^6^

^1^Department of Psychiatry, Istanbul Faculty of Medicine, Istanbul University, Istanbul; ^2^Department of Psychiatry, Cerrahpasa Medical Faculty, Istanbul University, Istanbul; ^3^Tokat State Hospital, Tokat; ^4^Cumra State Hospital, Konya; ^5^Tapdi Buca Medical Center, Izmir; ^6^Istanbul Bilgi University, Istanbul

The aim of this study was to inquire into the relationship of childhood trauma and dissociation with lifetime diagnosis of major depressive disorder among women who suffer from fibromyalgia (N=30) or rheumatoid arthritis (N=20). Childhood Trauma Questionnaire (CTQ-28), Somatoform Dissociation Questionnaire (SDQ-20), Dissociation Questionnaire (DIS-Q), Beck Depression Inventory (BDI), Spielberger State-Trait Anger Expression Inventory (STAXI), and Dissociative Disorders Interview Schedule (DDIS) were administered to all participants. Among women with fibromyalgia or rheumatoid arthritis, depressive patients had elevated scores on both somatoform and psychological dissociation but childhood trauma scores did not differ between depressive and non-depressive groups. In regression analysis, somatoform dissociation (SDQ) predicted lifetime diagnosis of major depression whereas psychological dissociation (DIS-Q) predicted current depression (BDI). Among childhood trauma types, somatoform dissociation was predicted by emotional neglect and psychological dissociation by sexual abuse. In conclusion, rather than childhood psychological trauma, dissociation is related to depressive disorder among women with fibromyalgia or rheumatoid arthritis. However, dissociation serves as a mediator in the process leading from childhood trauma to depressive disorder.

### Borderline personality disorder: the current status of the BPD diagnosis and its proposed relationship to attachment disruption and childhood trauma

#### G. Mapel New York University, Washington Square, New York, USA

The purpose of this review is to elucidate the roles of insecure attachment and childhood trauma in development of borderline personality disorder (BPD), further informing discourse concerning the validity of the BPD diagnosis and its *DSM* status as a personality disorder. The author provides evidence in support of (1) the substantial contribution of both insecure attachment and childhood trauma to BPD and (2) a model of BPD development that incorporates both factors. Evidence from empirical and theoretical works was drawn from published sources in texts and peer-reviewed journals. Findings suggest that there is reason to propose a model of BPD development in which attachment trauma creates in the individual a vulnerability to abuse, ultimately contributing to the constellation of symptoms currently referred to as “borderline personality disorder.” Further, it is argued that the aforementioned manifestation of BPD actually reflects posttraumatic sequelae, and should be referred to as such, requiring re-visitation of Herman's (1992) concept of “complex posttraumatic stress disorder” as well as multivariate, longitudinal research.

References

Main, M., Hesse, E., & Kaplan, N. (2005). Predictability of attachment behavior and representational processes at 1, 6 and 19 years of age: The Berkeley Longitudinal Study. In Grossman, Grossman & Waters (Eds.) *Attachment from Infancy to Adulthood* (pp. 245–305). New York: The Guilford Press.

Stern, A. (1938). Psychoanalytic investigation of and therapy in the border line group of neuroses. *The Psychoanalytic Quarterly, 7*, 467–489.

Van der Kolk, B. A., Perry, J. C., & Herman, J. L. (1991). Childhood origins of self-destructive behavior. *American Journal of Psychiatry, 148*(12), 1665–1671.

### Mental health sequelae of childhood sexual abuse

#### A. Muenzer, J. M. Fegert and L. Goldbeck Department of Child and Adolescent Psychiatry/Psychotherapy, University Ulm Medical Centre, Ulm, Germany

*Objectives*: Child sexual abuse (CSA) may have a devastating impact on mental health and is likely to nurture later psychological disorders. The exploration of victims who nevertheless do not develop psychopathological symptoms aims to identify resilience factors in the aftermath of CSA. There is a lack of research assessing the mental health and resilience of minor victims. Funded by the German Federal Ministry for Family Affairs, Senior Citizens, Women and Youth, this study aims to identify factors related to the mental health of minor CSA survivors. Mediators and moderators, such as abuse characteristics, perceived social support, or maladaptive cognitive appraisals, are taken into account. Additional information is collected about the process of disclosure and its possible implications for the survivors’ mental health. *Methods*: Currently, study participants between 7 and 17 years of age are being recruited in collaboration with the healthcare and child welfare systems. A structured clinical telephone or face-to-face interview assessing the experience of CSA (Juvenile Victimization Questionnaire) and lifetime psychopathological symptoms (Kiddie-SADS-PL) is conducted. In addition, the study participants as well as their non-abusive caregivers separately fill out questionnaires to assess posttraumatic stress symptoms as well as potential resilience factors. *Results*: In this contribution, we present results on the feasibility of our study, sample characteristics, and first results on the prevalence of resilience and of the most frequent mental disorders. *Conclusions*: The data will provide information about CSA survivor's psychosocial adaptation and resilience. Protective factors to be found might indicate additional strategies for prevention and intervention.

### Type of trauma, alexithymia and dissociation, and their impact on the process of adaptation after trauma

#### E. Zdankiewicz-Scigala Warsaw University of Social Sciences and Humanities, Warsaw, Poland

The aim of this study was to verify the assumptions about the impact of the type of trauma experienced on post-traumatic disorder and posttraumatic growth. As moderating variables, dissociation and alexithymia were included. To verify the assumptions, a group of 47 people of both sexes was examined. The study was conducted in a person who has experienced one of the types of trauma: trauma associated with rape or sexual violence, trauma associated with the death of a loved one, or the trauma associated with being a victim of a fire, accident, etc., in the last three years. All individuals were asked to complete the following questionnaire for the measurement of PTSD, alexithymia, dissociation, and posttraumatic growth. The analysis showed that the amount of posttraumatic disorder as a result of lived trauma is the highest in the group of people who have experienced sexual trauma. Regression analysis showed that the dissociation is very important to the development and maintenance of the symptoms associated with the criterion B and C. The alexithymia was a significant predictor for the criterion D. The high levels of alexithymia were associated with deeper problems of affective arousal and regulation of affect after trauma. However, regression analysis revealed that the increase in the level of trauma is important in traumatic disorders (according to the B, C, D symptoms criteria) and the overall level of dissociation. If the disorder is greater, then the posttraumatic growth is smaller.

### Traumatic symptomatology and cognitive distortions among male victims of physical and sexual childhood abuse and intimate partner violence

#### M. Karlsson, P. Petretic, J. Henrie and M. Calvert University of Arkansas, Fayetteville, Arkansas

Childhood abuse and intimate partner violence (IPV) victimization have been related to several negative outcomes (Black et al., 2011; Coker et al., 2002). However, few studies have investigated male victims, which limit the understanding of victimization in general. This study recruited male college students (N=95) to compare victims to non-victims on trauma symptomatology and cognitive distortions. Twenty-one males (22.1%) reported being victims of child abuse; 33.3% victims of physical abuse only, 47.6% of sexual abuse only, and 19.0% of both physical and sexual abuse. Sixty males (63.1%) reported being victims of IPV; 21.7% victims of physical IPV only, 28.3% of sexual IPV only, and 33.3% of bothphysical and sexual IPV. In contrast to research indicating significant differences, this study found few differences between male victims of childhood abuse and non-victims. Only victims of childhood sexual abuse reported significantly more trauma symptoms and cognitive distortions; surprisingly, they reported more symptoms than victims of both types of abuse. While victims of sexual and/or physical IPV endorsed more trauma symptoms and cognitive distortions compared to non-victim, the strongest effect was found for males reporting both types of IPV victimization. Cognitive distortion patterns will be discussed for all victimization groups. Implications for research and clinical work with male victims will be discussed.

References

Black, M. C., Basile, K. C., Breiding, M. J., Smith, S. G., Walters, M. L., Merrick, M. T., et al. (2011). *The National Intimate Partner and Sexual Violence Survey (NISVS): 2010 Summary Report*. Atlanta, GA: National Center for Injury Prevention and Control, Centers for Disease Control and Prevention. Retrieved from http://www.cdc.gov/ViolencePrevention/pdf/NISVS_Report2010-a.pdf

Coker, A. L., Smith, P. H., McKeown, R. E., & King, M. J. (2000). Frequency and correlates of intimate partner violence by type: Physical, sexual, and psychological battering. *American Journal of Public Health, 90*, 553–559. doi: 10.2105/AJPH.90.4.553

## Effects of trauma on families and children

### The effects of traumatic stress on parental involvement in schools

#### D. Silva^1^, C. Moreira^1^, C. Rocha^2^, J. Rocha^3^, A. Silva^1^ and L. Leal^1^

^1^Universidade Portucalense Infante D. Henrique, DPCE, Porto; ^2^Câmara Municipal de Penafiel, Penafiel; ^3^UnIPSa-CICS, InstitutoSuperior de Ciências da Saúde – Norte, CESPU, Porto

Parental involvement on school activities is a known important aspect to promote children achievement; however, there is lack of research on parent'sdifficulties that may explain why some parents fail to provide the support that children need. Several adverse conditions may contribute to limit parental involvement, specifically, traumatic stress symptoms may be important to explain school involvement variations. This study aims to verify the nature of the correlations between parental school involvement and traumatic stress symptoms. In this study, 74 parents, 54 mothers (74%) and 19 fathers (26%), aged between 24 and 50 years (M=38.32, SD=6.19), from schools at Penafiel municipality, northern Portugal, participated, who responded to the questionnaire of Parental Involvement in School—Parent Version (QEDE-VPA). This instrument measures parental involvement in school volunteering activities, parental involvement in learning activities at home, school-family communication, and participation in parents' meetings. The Impact of Event Scale—Revised (IES-R) measured traumatic stress symptoms. The main result reveals a significant negative correlation (r=−0.567) between overall parental involvement and traumatic stress. Other correlations between instruments’ subscales are also negative. These results highlight the complexity and intergenerational nature of traumatic stress effects. Also, it considers parents traumatic stress as an important target on School Involvement programs, particularly on highly traumatized communities. There is need to raise awareness of this issue on educational community. Results are discussed considering limitations, future plans, and implications to practice.

### Children's Revised Impact of Event Scale: Portuguese version, psychometric characteristics and usefulness in school context

#### C. Moreira^1^, D. Silva^1^, C. Rocha^2^ and J. Rocha^3^

^1^Universidade Portucalense Infante D. Henrique, DCEP, Porto; ^2^CâmaraMunicipal de Penafiel, Penafiel; ^3^UnIPSa-CICS, InstitutoSuperior de Ciências da Saúde – Norte, CESPU, Porto

The Children's Revised Impact of Event Scale (CRIES) is a brief child-friendly measure designed to screen children at risk for posttraumatic stress disorder (PTSD) and has been used to screen very large samples of at-risk-children following a wide range of traumatic events. In the absence of an instrument to screen at-risk children for PTSD, particularly on school context with specific adversities such as peer violence, excessive punishment, watch an act of violence, among others, we assume relevant possibilities on an earlier screening to enable prevention of future consequences. We aim to translate and to verify psychometric characteristics of the Revised Child Impact of Event Scale-Portuguese version, and to check its usefulness on school context events impact in children. To achieve these goals, we translated, blind back-translated, and achieved agreement between versions. We asked for consent of 120 children with ages between 12 and 16 years on Penafiel Municipality schools, in northern Portugal. We used a sociodemographic questionnaire, a list of stressful events, and the Portuguese version of CRIES. We used reliability analysis and factor analysis to verify main psychometric characteristics of CRIES 13-item version. Also, bivariate analysis of each stressful event exposure and CRIES results is achieved to pursue an external level of validity. The importance of traumatic stress symptoms assessment in children has been emphasized, with special applicability in schools, considering the effects of several contextual events and its relevance on children well-being, mental health, and academic achievement. The implications to practice, to guidelines for school contingency plans, and future research are discussed.

### Multiple victimization among women: symptomatology, trauma, and resilience

#### M. Matos, R. Conde and A. Maia University of Minho, Braga, Portugal

The dominant model in victimology research has focused mainly on the individual forms of victimization, neglecting their cumulative effect. Thus, this study aims to characterize women who experienced multiple victimization, describe the types and patterns of victimization suffered, and the impact of victimization on their psychological and mental health. The study integrates 30 women aged between 18 and 64 years in a deprived social and economic condition, who suffered violence in the last 12 months, as well as other forms of victimization throughout life. We used a structured interview to evaluate the violence they suffered throughout life, two scales to measure sexual and partner violence in the past year, and two questionnaires to assess symptomatology. We conclude the high prevalence of various forms of victimization accumulated, from childhood to adulthood. There was a significant increase of victimization in adulthood, where mainly the partner perpetrated the most serious forms of violence. Participants did not give evidence for significant clinical symptoms. Several hypotheses’ are discussed concerning this results, namely possibility that women try to convey an image of strength and endurance, as well as the fact that the vast majority are living in shelters that gives them a sense of current safety and welfare.

### Anxiety in children who experienced different forms of abuse

#### B. Profaca, G. Buljan Flander and A. M. Spanic Child Protection Centre of Zagreb, Zagreb, Croatia

Anxiety is one of the most common consequences of child abuse. The aim of this study was to examine different aspects of anxiety symptoms: separation anxiety, social anxiety, test anxiety, obsessive-compulsive symptoms, worry, anxiety sensitivity, and somatic symptoms in abused children of different ages. A clinical study, conducted in the Child Protection Centre of Zagreb, involved three groups of abused children aged between 9 and 18 years: (1) emotionally abused children (N=62), (2) sexually abused children (N=84), and (3) both physically and emotionally abused children (N=49). *The Fear and Anxiety Scale for Children and Adolescents* (SKAD-62; Vulic-Prtoric, 2004) was used. It is a 62-item self-report measure divided into 7 subscales/aspects of child and adolescent anxiety. Differences in various aspects of anxiety were examined for younger children (9–13 years) and older children—adolescents (14–18 years). Among younger children, physically and emotionally abused children have higher scores on the anxiety sensitivity scale compared to the other two groups of the same age. Differences within each group of abused children were tested as well. Results show that in the group of physically and emotionally abused children, younger children show more signs of separation anxiety, obsessive-compulsive actions, anxiety sensitivity, and somatic symptoms than older physically and emotionally abused children. This research also considers above-average results on each anxiety subscale in three groups of abused children with regard to age. Among younger physically and emotionally abused children, more separation anxiety is found in relation to emotionally abused children of the same age. Older sexually abused children show significantly more symptoms of social anxiety compared to emotionally abused children of the same age. Comparison of younger and older children with above-average results within each type of abuse will also be presented. All results will be discussed with respect to its treatment implications.

### Personal and social resources in the offspring of former political prisoners in East Germany (1945–1989)

#### G. Klinitzke^1^, M. Boehm^2^, E. Braehler^2^ and G. Weissflog^2^

^1^Department of Mental Health, Clinic and Policlinic of Psychosomatic Medicine and Psychotherapy, University of Leipzig, Germany; ^2^Department of Mental Health, Medical Psychology and Medical Sociology, University of Leipzig, Germany

*Purpose*: Between 1945 and 1989, more than 300,000 people were incarcerated for political reasons (e.g., the illegal crossing of the border, contact to persons in the western countries, etc.) in East Germany. Traumatic maltreatments led to physical and psychological long-term consequences. It seems that the detainees’ offspring are more vulnerable for mental disorders, but there is only little information about their personal and social resources. *Methods*: In a cross-sectional study, 43 persons whose parents were in prison for political reasons in East Germany (1945–1989) were recruited in a multimodal way (media, internet, memorials). Individuals took part in a postal survey conducted in 2010. Resources (resilience [RS-11], social support [OSS-3/ESSI]), psychopathological variables (depression [PHQ-9], somatoform disorders [PHQ-15], anxiety [GAD-7]), and imprisonment-related variables (e.g., one or both parents, one or more times, child was born before/while or after detainment) were assessed. *Results*: Offspring of former political detainees indicated less resilience compared to a representative sample. Less resilient participants reported more psychopathological symptoms than high resilient ones. The levels of received and perceived social support they reported were comparable to levels in a German representative sample. None of the imprisonment-related variables were significantly associated with one of the resources. Resilience and social support were negatively associated with psychopathological symptoms. *Conclusion*: Compared to the general population, the offspring of former political prisoners reported less resilience but social support on a similar level. Social support and resilience were inversely related to psychopathological symptoms. Hence, it seems that personal and social resources are protective factors in this population. The interaction between resources, psychopathology, and relevant confounding variables needs further investigation. The results of our study add to the sparse research on political imprisonment in East Germany.

### Crisis intervention in the acute phase after trauma: risk factors, reactions, and subjective needs

#### E. Mohr^1^, B. Juen^1^ and H. Siller^2^

^1^Department of Psychology, University of Innsbruck, Innsbruck, Austria; ^2^Women's Health Center at Innsbruck Medical University Hospital, Innsbruck, Austria

The acute team offers support for relatives after acute loss of a loved one. A team of psychologists and social workers carries out this support. In a study on 426 cases of acute losses taken from the documentation of the acute team in lower Austria, we examined the following variables: Risk factors: history (previous traumata, psychiatric illness in the past, more than one loss within a short time, etc.); personal factors (bad physical status, chronic illness, high dependency, etc.); situational factors (intensity, duration, intensity of helplessness, etc.); and social risk factors (accusation, lack of social support, etc.). The focus was set specifically on two areas: problematic acute reactions and interventions. Problematic acute reactions include extreme forms of avoidance, extreme and continuing arousal, panic attacks, continuing numbness, etc. Interventions covered safety (staying with the client, being available for the client, listening to the client, etc.), connectedness (helping to use social networks, enhancing positive social support, etc.), calm (helping to reduce arousal, psycho-education, etc.), self-efficacy (coaching through the situation, helping to stay active and take decisions, etc.), hope (helping to go on with life and referral to further support systems). The results showed a high satisfaction with the interventions as well as highly significant correlations between certain risk factors and certain problematic reactions like, for example, the tendency to react with panic attacks and a psychiatric illness in the past. Regression analysis showed that the situational risk factors can best predict problematic acute reactions. A highly significant result could also be found between the interventions that promote “calm” (mainly psychoeducation) as predictor and the client′s satisfaction with the intervention. Implications for practical work are deduced from the results.

### Subjective assessment of childhood abuse and neglect in the course of psychotherapy

#### J. Schellong, A. Symmank, I. Croy and K. Weidner Department of Psychosomatic Medicine and Psychotherapy, Medical Faculty Technical University of Dresden, Dresden, Germany

*Background*: Childhood abuse and neglect is supposed to be a strong risk factor in the development of physical and mental health problems in later life periods. Among those reporting maltreatment, multicategory abuse seems to be the norm rather than the exception. The exposure to multiple types of abuse and the number of different types of maltreatment are critically for the outcome in later life. Apart from the immediate harassment of physical injuries, the additional experience of neglect and emotional humiliation is thought to be the pivotal element of weak self esteem, severe relational disturbances, and decrementing mental health scores in adulthood. But, how do those who are affected see their own difficult childhood and does reflection change in the course of psychotherapy? *Methods*: Thirty two female inpatients (age mean 39±10.9) were assessed with the Childhood Trauma Questionnaire (CTQ) and questionnaires for psychosomatic disorders before and after treatment. Twenty nine healthy women (age mean 36±10.4) served as control. *Results*: After therapy, the patients exhibited significantly reduced derealisation, reduced symptoms of posttraumatic stress disorder as well as reduced anxiety and depression. However, awareness of childhood maltreatment increased, indicated by enhanced rating in the CTQ subscales emotional abuse and emotional neglect and reduced trivialization. No significant change was observed in any of the questionnaire results in the healthy controls indicating that the effects found in psychosomatic patients are due to psychotherapy. *Conclusion*: Psychotherapy goes along with higher reflection. A more critical interpretation of early relationships does not contrast to improvement. Instead, the individual identification of emotional maltreatment in addition to physical abuse may play an important role in the course of psychotherapy.

### Polyvictimization and trauma symptoms in a sample of catalan youth

#### L. Soler, M. Forns, T. Kirchner and A. Segura
University of Barcelona, Spain

Several studies have noticed that focusing merely on one type of the large spectrum of victimizations adolescents can suffer has several important limitations. First, it is likely to underestimate the full burden of victimization adolescents are actually exposed to. Second, a narrow focus on specific types of victimization hampers the identification of the most highly victimized children (and thus, those at greatest risk for serious mental health problems). Third, this fragmented approach can lead to a serious overestimation of the impact of individual victimization experiences (since outcomes may be related to other victimizations or their co-occurrence rather than individual victimization events). This study aims at studying the strength of the associations between different kinds of victimization (e.g., sexual victimization) and total trauma symptoms taking into account the full range of victimizations adolescents suffer. The final aim is to clarify those kinds of victimization whose role in explaining trauma symptoms may have been overestimated by studies that do not take into account other kinds of victimization. A total of 804 victimized adolescents (*M*=15.74 years; *SD*=1.19) were recruited from eight different secondary schools in Catalonia. The Juvenile Victimization Questionnaire (JVQ) and the Youth Self Report (YSR) were employed to assess victimization and posttraumatic stress symptoms, respectively. Results indicated that, in girls, Peer and Sibling Victimization, Sexual Victimization, and Indirect Victimization lost weight at explaining trauma symptoms when the whole range of victimization were taken into account. In boys, the same happened with Conventional Crime, Sexual Victimization, and Indirect Victimization. These results are in line with prior research and highlight the importance of taking into account all the kinds of victimization adolescents can suffer when studying its association with mental health issues.

### Do children of veterans with chronic posttraumatic stress disorder have more emotional and behavioral disturbances?

#### A. Kastelan^1^, J. Grkovic^2^, I. Roncevic Grzeta^2^, Z. Sukovic^2^ and T. Franciskovic^1^

^1^Department of Psychiatry, University School of Medicine, Rijeka, Croatia; ^2^Clinical Hospital Center, Rijeka, Croatia

The objective of this study was to determine if the children of veterans suffering from chronic posttraumatic stress disorder (PTSD) have more emotional and behavioral disturbances than those of veterans without PTSD. The research involved 70 veterans with PTSD and 70 without PTSD and their spouses. The PTSD symptoms in father were assessed by the Clinical-Administrated PTSD Scales (CAPS). The mothers assessed the disturbances in their school-aged children through the Child Behavior Check List (CBCL). Both groups of children did not reach clinically significant cutoff scores on CBCL. However, the children of veterans with PTSD had significantly higher scores on scales of withdrawal, anxiety, and depression.

### Polydrug use typologies and childhood maltreatment in a nationally representative survey of Danish young adults

#### C. Armour^1^, G. Shorter^2^, J. Elhai^3^, A. Elklit^1^ and M. Christoffersen^4^

^1^University of Southern Denmark, Odense, Denmark; ^2^University of Ulster, Londonderry, UK; ^3^University of Toledo, Ohio, USA; ^4^National Centre of Social Research, Denmark

Childhood maltreatment is known to associate with substance use during adolescence and early adulthood. Variations may be explained by sex of the subject. Three latent class analyses were performed on eight types of illicit drugs for the total random sample of the population of young Danes (*n*=2,980) and males (*n*=1,555) and females (*n*=1,425) separately. Logistic regression was performed to assess associations between patterns of polydrug use, sociodemographic characteristics, and four types of childhood maltreatment. A three-class solution best described patterns of polydrug use in all samples. A differential pattern of associations between latent classes, sociodemographics, and maltreatment variables was demonstrated across samples. Albeit males and females have similar drug use patterns; these patterns have differential relationships with external correlates, most notably childhood maltreatment experiences.

### Intergenerational transmission of dysfunctional relationship: The case study of a mother–daughter dyad with violent partner

#### A. Merenda, P. Miano, R. Damiano and A. Salerno Department of Psychology, University of Palermo, Italy

The effects of trauma are cumulative and related to mental functioning and various dimension of personality and involve different variables such as attachment, behaviour, dissociation, as well as the level of adjustment (Fonagy, Moran & Target, 1993). Parenting skills are also negatively influenced by traumatic experiences that deteriorate symbolic competence and secure relationships. Specifically, parents’ failure in elaborating traumatic experiences leads to increased vulnerability in children who tend to develop dysfunctional relational pattern and emotional dysregulation (Tronick, 1989). According to this framework, the aim of our research was to investigate early traumatic experiences and attachment styles in a sample of five mother–child dyads, who were living in a safe house for women and children escaped from domestic violence. *Methods*: Intergenerational transmission of trauma has been evaluated by direct observation of behavioural indicators, cognitive competence, physiological responses to stress, and depressive and anxiety symptoms. *Measures*: Adult Attachment Interview—administered to mothers, aged 16–44 years; Attachment Q-Sort or Separation Anxiety Test—administered to children, aged 5–15 years. *Results*: One of the participants was a mother of 16 years old, whose mother (44 years) and daughter (1 year) lived in the same safe house. Their experiences showed the transmission of dysfunctional relationship pattern. The results of the daughter at the AQS were −1.02, both grandmother and mother were classified as “Dismissing” at the AAI and they both described their partner as physically and psychologically violent.

References

Fonagy, P., Moran, G. S., & Target, M. (1993). Aggression and the psychological self. *International Journal of Psycho-Analysis*, *74*, 471–485.

Tronick, E. Z. (1989). Emotions and emotional communication in infants. *American Psychologist*, *44*(2), 112–119.

### Operation of selective notes at the victims and perpetrators of traffic accidents

#### D. Scigala
Warsaw University of Social Sciences and Humanities, Warsaw, Poland

The study was intended to find differences in the perceived level of anxiety and the perception of words related to a car accident, between people involved in a road accident, in which month has passed since the event and the control group of drivers. To verify the above problem, testing regime was constructed consisting of a test version of the modified emotional Stroop test and the questionnaire. In the study, 93 people attended, 43 people after an accident and 50 in the control group. As expected, those with a research group achieved significantly longer reaction times to name the ink color of which are written words evocative of the accident, who survived F (1,94)=7.173, p<0.01 despite the fact that the average age of the study group was lower and the reaction time is known to deteriorate with age.

## Impact of trauma on communities

### Relationship between traumatic stress and appraisal of social context in Lithuania: pilot study

#### P. Zelviene and E. Kazlauskas Department of Clinical and Organizational Psychology, Vilnius University, Lithuania

*Introduction*: Social context can be significantly related with traumatic stress not only on individual, but also on community level. Lithuania restored its independency only 20 years ago and as well as other Eastern Europe states started to build new social structures and develop new social rules. All population had to adapt to the new changing social context. The aim of this study was to evaluate relationship between traumatic stress and appraisal of social contextual factors in a sample of Lithuanians from general population who were exposed to traumatic events. Research was funded by a grant (No. SIN-01/2012) from the Research Council of Lithuania. *Methods*: A sample of 59 participants, mean age 34.21 (SD=14.68) range from 19 to 76 years, 67.8% women, 32.2% men participated in a pilot study. Appraisal of social context was measured by self-report Social Changes Inventory (SOCHI) developed by the authors of this study (Kazlauskas, Zelviene, 2012). Measures also included perceived lifetime trauma exposure, traumatic stress reactions were measured using Lithuanian version of Impactof Event Scale—Revised (IES-R). *Results*: Pilot study results supported our prediction that traumatic stress reactions are related with appraisals of social context. Traumatic stress reactions significantly correlated with insecurity related with perceived constant threats for the country from outside, uncertainty about the future of the country, hopelessness that people in country were never happy, insecurity that you cannot trust anybody and could be betrayed by somebody, and that one cannot share his own ideas and thoughts with others. According to this finding, we conclude that negative appraisal of social context in Lithuania is significantly related with traumatic stress.

### Prevalence rate of post-traumatic stress disorders (PTSD) and other psychological disorders among Saudi firefighters

#### Mohammed Alghamd^1^, Nigel Hunt^2^ and Shirley Thomas^3^ ^1^Clinical Psychology, Division of Psychiatry and Applied Psychology, School of Medicine, University of Nottingham, Nottingham, UK; ^2^Division of Psychiatry and Applied Psychology, School of Medicine, University of Nottingham, Nottingham, UK; ^3^Rehabilitation Psychology, Division of Rehabilitation & Ageing, School of Medicine, University of Nottingham, Nottingham, UK

*Background*: Firefighters have a high probability of being exposed to a variety of traumatic events. Potentially traumatic events can occur during a single rescue such as: providing aid to seriously injured or helpless victims. Moreover, firefighters who are injured in the line of duty may have to retire as a consequence of their injury. The psychological cost of this exposure may increase the risk of long-term problems, such as post-traumatic stress disorder (PTSD) symptoms, depression, and anxiety. *Objective*: The purpose of this study was to investigate the prevalence of PTSD symptoms, depression, anxiety, and assess related variables such as coping strategies and social support among Saudi firefighters. *Method*: Two hundred firefighters completed the Fire-fighter Trauma History Screen (FTHS) to measure the number of traumatic events, Screen for Post-traumatic Stress Symptoms (SPTSS) scale to assess the prevalence of PTSD symptoms, Hospital Anxiety and Depression Scales (HADS) to assess depression and anxiety, Brief Cope (BC) scale to measure coping strategies used, and Social Support scale was used to evaluate the firefighter’s support received. *Results*: The results showed that 84% (169/200) of firefighters were exposed to at least one traumatic event. The result presented that 57% (96/169) of exposure firefighters fully met the DSM-IV criteria for PTSD with high levels of depression and anxiety; 39% (66/169) partially met the PTSD criteria. However, only 4% participants have not met the PTSD criteria. The results also revealed that adaptive coping strategies and higher perceived social support was associated with lower levels of PTSD. *Conclusion*: The high prevalence rate of PTSD related to the type and severity of the traumatic events and years of experience in the job. Accordingly, many firefighters were severely affected by their experiences, and we should be developing methods to help them.

Poster added after first publication of the supplement.

## Evidence-based practice on trauma

### Early intervention for psychological trauma: facilitating evidence-informed practice within a provincial health system

#### L. Hawkins, S. Rawlings and K. Corrigan Alberta Health Services, Canada

Evidence indicates that early responses to individuals following a potentially psychological traumatizing event would best align with a practical approach to providing information and supportive care, with referral to mental health services only when indicated or requested. This approach, which recognizes both the literature on human resilience posttrauma, as well as the potential negative impact associated with psychological debriefing techniques, represents for many service providers a significant change in practice. A need was identified within a large-scale Canadian provincial health care system (Alberta Health Services) to identify current evidence-informed best practice with respect to early interventions posttrauma and support service providers to align practice with this current standard of care. In order to achieve these outcomes, a working group was struck involving leaders and service providers from each of the five regional zones, with representation including EMS and a representative from the organization's contracted Employee and Family Assistance Program. Following a comprehensive literature review, international benchmarking, and an internal environmental scan of current practice throughout the provincial health care system, a Practice Guideline was developed, and recommendations were put forth to facilitate the uptake of the Practice Guideline within programs and by staff. An online Psychological Trauma Toolkit was developed, containing links to the practice guideline, as well as sections with information specific to managers, service providers, and all staff. The Toolkit provides basic information about psychological trauma, how to assist someone who has been recently exposed to a potentially traumatic event, and recovery and building resilience. In addition, multiple resources and links are provided, both to facilitate a trauma-informed perspective as well as to highlight opportunities for training in Psychological First Aid (PFA), an approach that most closely aligns with the developed Practice Guideline.

### The German CANMANAGE consortium: implementation of managed mental healthcare for children and adolescents after abuse and neglect

#### H. G. Ganser^1^, S. Von Jahn^2^, J. M. Fegert^1^, A. Muenzer^1^, P. Plener^1^, R. Rosner^2^, A. Witt^1^ and L. Goldbeck^1^

^1^Department of Child and Adolescent Psychiatry/Psychotherapy, University Hospital Ulm, Ulm, Germany; ^2^Catholic University Eichstätt-Ingolstadt, Eichstätt, Germany

Victims of child abuse and neglect (CAN) have a high risk to develop mental disorders. Some survivors however are resilient despite exposure to CAN. The CANMANAGE consortium, funded by the Federal Ministry of Education and Research, addresses the need to improve dissemination of evidence-based treatments for CAN survivors with clinically relevant trauma sequelae (study 1) as well as the need for longitudinal studies helping to further understand risk and resilience factors (study 2). Further associations of migration background with long-term mental health outcomes of CAN survivors are examined (study 3). Study 1, a randomized controlled intervention study, investigates the effectiveness of a structured CAN-specific case management at the interface between the child welfare and the healthcare system to improve utilization of evidence-based treatments for CAN-survivors. Specific efforts to engage CAN-survivors with migration background in clinical management and treatment will be tested for their effectiveness in study 3. Study 2 examines underlying processes of resilient functioning and aims to identify distinct latent classes of symptom trajectories in survivors of CAN. The consortium will recruit 500 participants aged 4–14 years with substantiated CAN and non-offending caregivers across five sites in Germany. Participants are assessed regarding trauma history, mental health, and risk and resilience factors and then followed up three times, i.e., 6, 12, and 24 months after inclusion. Participants with clinically relevant mental disorders are included in the randomized controlled intervention study. An additional intervention program will be implemented to overcome specific barriers to treatment for families with migration background. Participants without clinically relevant mental health problems are followed up in the resilience study. The studies will gain new insights in developmental-specific mechanisms of vulnerability and resiliency of children in the aftermath of CAN and provide conclusive results on the effectiveness of a method of managed mental healthcare for so far underserved CAN victims.

### PTSD and primary health care: a qualitative approach to explore General Practitioners perspectives and experiences

#### M. Sa^1^ and J. Rocha^2^

^1^InstitutoSuperior de CiênciasBiomédicas Abel Salazar—Universidade do Porto, Portugal; ^2^InstitutoSuperior de Ciências da Saúde- Norte, Cespu—Gandra, Portugal

The Portuguese survey included on the National Study of Mental Health indicates a prevalence rate for mental disorders of 22.9%. The majority of mental health cases are detected through primary health care system, and 10–20% of people use the primary health care because of complaints related to psychological disturbance. Posttraumatic stress disorder (PTSD) prevalence rate was 7.87% in the Portuguese population. PTSD is a potentially debilitating anxiety disorder triggered by exposure to a traumatic experience with high negative effects on general health and on quality of life, long-term impacts implicating high economic cost to health system. Because of the complexities associated with assessment and treatment PTSD, several doubts arise on the role of General Practitioners (GPs) to identify and decide the best care approach. A qualitative research surges as a powerful resource to describe how PTSD cases are processed in primary health care by GPs, focusing on how they conduct assessment, diagnosis, intervention/treatment, follow-up and refers. This study uses a qualitative design to explore GPs beliefs underlying decision-making and their practices. Semi-structured interviews are conducted with 20 GPs. The semi-structured interview is designed to be flexible and broad, yet effective to generate meaningful results concerning four specific dimensions: assessment procedures, diagnosis, interventions, follow-up and refers. Results from interviews are analyzed with a thematic referrals to determine common ideas and themes on each dimension. Reported difficulties are discussed to provide solutions for best care to patients considering current guidelines to manage PTSD patients.

### Vilnius study on effects of brief eclectic psychotherapy for posttraumatic stress disorder: pilot study results

#### E. Kazlauskas, E. Mazulyte, P. Zelviene, P. Skruibis and M. Dovydaitiene
Department of Clinical and Organizational Psychology, Vilnius University, Lithuania

*Introduction*: The brief eclectic psychotherapy for posttraumatic stress disorder (BEPP) is a promising approach for treatment of PTSD developed by Berthold Gersons (Gersons et al., 2011). Several studies confirmed the effectiveness of BEPP (Gersons & Carlier, 2004; Lindauer et al., 2005; Olff et al., 2007). Recent study in Amsterdam supported its efficacy in comparison with EMDR (Nijdam et al., 2012). Vilnius study was started to evaluate the efficacy of BEPP and the effects of BEPP in Lithuanian sample of patients with PTSD. This research was funded by a grant (No. MIP-011/2012) from the Research Council ofLithuania.The main goal of this presentation is to present results of subjectively perceived effects of BEPP in a pilot study. *Methods*: Small sample from a general population with various traumatic experiences participated in a pilot study. All participants were included into the study based on CAPS results and meeting the criteria for PTSD. BEPP was delivered by three experienced PhD level clinical psychologists, trained in BEPP by B.Gersons. Continuous supervisions were organized to ensure validity of BEPP. Impact of Event Scale—Revised (IES-R) and CORE-OMwere used for therapeutic changes assessment, and Working Alliance Inventory was used to measure the quality of therapeutic alliance. Also, qualitative data from clients during intermediate assessments about attributions of therapeutic changes and satisfaction with treatment were collected. *Results*: Pilot study supported the effectiveness of BEPP and the significant therapeutic changes in PTSD levels, well-being, and functioning. In comparison to IES-R measurements, clients reported subjectively more optimistic changes. Clients attributed number of factors contributing to the therapeutic change, including psychoeducation, role of the therapist, and personal involvement.

### The protective role of perceived social support for intrusion and numbing - data in survivors of institutional abuse

#### A. Butollo, Y. Moy, R. Jagsch, V. Kantor, D. Weindl and B. Lueger-Schuster University of Vienna, Vienna, Austria

*Background*: Perceived social support (PSS) is a protective factor against psychological problems and disorders. Survivors of institutional abuse in the Catholic Church in Austria participated in this study.They recalled their PSS in three dimensions: perceived emotional support (PES), perceived practical support (PPrS), and perceived social integration (PSI). *Method*: We developed the Recalled Perceived Social SupportQuestionnaire (RPSSQ) to measure PSS in this specific sample. Additionally survivors answered the PTSD Checklist-Civilian Version (PCL-C). A one-way ANOVA with PSS as factor and a correlation between PSS and the *Diagnostic and Statistical Manual of Mental Disorders*, Fourth Edition (DSM-IV) criteria B to D was computed. In this study, we focused on the impact of different types of PSS and their relationship to the PTSD criteria B (intrusions), C (avoidance/numbing), and D (hyperarousal). *Results*: One-hundred and eighty five persons filled in the questionnaires. The theoretical three-factor structure of the RPSSQ was supported by the results of an exploratory factor analysis. 48.4% of the sample fulfilled the DSM-IV criteria for PTSD. There were two surprising results: (1) There was a significant influence of the level of PSS on criteria B (d=0.48) and C (d=0.45), but there was no significant connection between PSS and criterion D. (2) There was a significant negative correlation between PES and the severity of PTSD. Moreover, there was a significant correlation between PSI and the severity of symptoms of PTSD. However, there was no significant relationship between PPrS and symptoms of PTSD, r=−0.088, p>0.05. *Conclusion*: There is no general protective effect of PSS on psychopathological symptoms. However, PES and PSI have a positive influence on intrusion and avoidance/numbing. Furthermore, PPrS seems not to beprotective against negative consequences of institutional abuse. The specific circumstances and clinical implications will be discussed.

### A qualitative study on recovering from shame in complex PTSD patients

#### C. Park, H. Joo and H. Ahn Department of Psychology, Ewha Womans University, Seoul, Korea

Although posttraumatic stress disorder (PTSD) is classified as an anxiety disorder and effective treatments have been developed aimed at reducing fear, this formulation seems accurate for single traumas but not for complex traumas that are repeated over a prolonged period of time. In the latter cases, shame is central to the victim's experience. The purpose of this study was to explore how complex trauma survivors that have had psychotherapy overcome from significant shame experiences. Semi-structured interviews composed of open-ended questions were conducted with a purposive sampling of 10 adults who had had traumatic events that elicited intense feelings of shame. Shame was conceptualized as an assault on the self. Grounded theory was used in the collection and analysis of the data. Results suggest that shame strikes at the core of the individual's being, with the most positive aspects of the self bearing the brunt of attack. Specifically, shame undermines the individual's positive self-concept and damages the individual's connection to others. Our participants overcame from their trauma experiences through the process of self-reconstruction. Rebuilding of the self emerged as the core category that represents the process of recovering from the shame. With rebuilding, individuals restore and expand their positive self-concept, and repair and strengthen their connections to the outside world. This process of rebuilding of the self was achieved through the therapeutic relationship with their therapists and by such processes as self-acceptance, self-empathy, contextualizing of self, reconnection with the others, and proactively reaching out. Although feelings of shame may not entirely disappear, they became marginalized from the core self and faded into the larger landscape of the individual's identity and experience. Implications for psychotherapy and directions for further research are discussed.

